# In-situ structure and catalytic mechanism of NiFe and CoFe layered double hydroxides during oxygen evolution

**DOI:** 10.1038/s41467-020-16237-1

**Published:** 2020-05-20

**Authors:** Fabio Dionigi, Zhenhua Zeng, Ilya Sinev, Thomas Merzdorf, Siddharth Deshpande, Miguel Bernal Lopez, Sebastian Kunze, Ioannis Zegkinoglou, Hannes Sarodnik, Dingxin Fan, Arno Bergmann, Jakub Drnec, Jorge Ferreira de Araujo, Manuel Gliech, Detre Teschner, Jing Zhu, Wei-Xue Li, Jeffrey Greeley, Beatriz Roldan Cuenya, Peter Strasser

**Affiliations:** 1 The Electrochemical Energy, Catalysis, and Materials Science Laboratory, Department of Chemistry, Chemical Engineering Division, Technical University Berlin, Strasse des 17. Juni 124, 10623 Berlin, Germany; 2 Davidson School of Chemical Engineering, Purdue University, West Lafayette, Indiana 47907 USA; 3 Department of Physics, Ruhr-University Bochum, Universitaetsstrasse 150, 44801 Bochum, Germany; 4 Department of Interface Science, Fritz-Haber-Institut der Max-Planck-Gesellschaft, Faradayweg 4 – 6, 14195 Berlin, Germany; 5 European Synchrotron Radiation Facility, ID 31 Beamline, BP 220, F-38043 Grenoble, France; 6 Department of Inorganic Chemistry, Fritz-Haber-Institut der Max-Planck-Gesellschaft, Faradayweg 4–6, 14195 Berlin, Germany; 7 Max Planck Institute for Chemical Energy Conversion, Stiftstrasse 34-36, 45470 Mülheim an der Ruhr, Germany; 8 CAS Excellence Center for Nanoscience, Hefei National Laboratory for Physical Sciences at Microscale, School of Chemistry and Materials Science, University of Science and Technology of China, Hefei, Anhui 230026 China

**Keywords:** Electrocatalysis, Energy, Density functional theory, Electrocatalysis

## Abstract

NiFe and CoFe (MFe) layered double hydroxides (LDHs) are among the most active electrocatalysts for the alkaline oxygen evolution reaction (OER). Herein, we combine electrochemical measurements, *operando* X-ray scattering and absorption spectroscopy, and density functional theory (DFT) calculations to elucidate the catalytically active phase, reaction center and the OER mechanism. We provide the first direct atomic-scale evidence that, under applied anodic potentials, MFe LDHs oxidize from as-prepared α-phases to activated γ-phases. The OER-active γ-phases are characterized by about 8% contraction of the lattice spacing and switching of the intercalated ions. DFT calculations reveal that the OER proceeds via a Mars van Krevelen mechanism. The flexible electronic structure of the surface Fe sites, and their synergy with nearest-neighbor M sites through formation of O-bridged Fe-M reaction centers, stabilize OER intermediates that are unfavorable on pure M-M centers and single Fe sites, fundamentally accounting for the high catalytic activity of MFe LDHs.

## Abstract (web summary)

NiFe and CoFe layered double hydroxides are among the most active electrocatalysts for the alkaline oxygen evolution reaction. Here, by combining *operando* experiments and rigorous DFT calculations, the authors unravel their active phase, the reaction center and the catalytic mechanism.

## Introduction

Water splitting to generate O_2_ and H_2_ has been a major focus of (photo)electrochemical energy storage and conversion research, but fundamental and practical challenges remain. In this process, O_2_ generation at the anode through the oxygen evolution reaction (OER), which is inherently slower by over four orders of magnitude compared with H_2_ generation, accounts for the majority of energy losses^1^. NiFe-based layered hydroxides are the most active OER catalysts in base and are the catalysts of choice for industrial water electrolysis^2–10^, whereas CoFe-based layered hydroxides have comparable performance^7,8,10–12^. Very recently, it has been found that NiFe and CoFe (MFe) layered (oxy)hydroxides are also the common active phases of other highly active OER catalysts, including perovskite oxides^13,14^, spinel oxides^15^, phosphides^16^, and potentially other Co- and Ni-based OER catalysts with Fe incorporated intentionally or accidentally, such as carbides^17^, nitrides^18^, sulfides^19^, and selenides^20^, which are prone to hydrolysis and oxidation under OER conditions^13,15,16,21,22^. Thus, studying the reactive structures of the MFe layered double hydroxides (LDHs) under in-situ conditions and the catalytic mechanism can provide a thorough understanding of the structure–property relationships of many related catalysts and potentially lead to the design of new catalysts with further improved performance.

In spite of previous reports on the ex-situ crystal structure of the as-synthesized precursors of MFe LDH catalysts^23–28^ and in-situ local structure based on X-ray absorption spectroscopy (XAS) measurements^3,4,12,29–32^, little is known about the long-range crystal structures of the catalytically active phase under OER conditions. As a result, most proposals regarding the in-situ crystal structures of NiFe and CoFe LDHs under OER conditions are indirectly inferred from the crystal structures of the host Ni and Co oxyhydroxides, respectively. More specifically, for NiFe LDH, a γ-NiOOH-type phase, in which water and cations are intercalated between layers^28^, has long been speculated^4,5,24,25,33^. However, no direct evidence has been observed to confirm this hypothesis, as previous in-situ structural studies could not provide the characteristic interlayer spacing that can be used to differentiate between the γ-NiOOH-type phase and other common phases, such as the anhydrous β-NiOOH-type phase^28^. For CoFe LDH, in analogy to NiFe LDH, a transformation to a γ-NiOOH-type phase can be hypothesized under OER conditions. However, there is no analogous γ-CoOOH phase with species intercalated between layers; the other two known β-CoOOH and CoO_2_ phases show no intercalation^34^. As a consequence, a Fe-doped β-CoOOH has been proposed as the active phase of CoFe LDH under OER conditions^11,12^.

Density functional theory (DFT) calculations allow us to examine all of the above hypotheses and to extract atomic-scale details by screening suitable candidate phases and comparing their relative stability with that of known phases. Although significant efforts have been made, particularly on the modeling of the electronic structure effects and catalytic mechanism of Ni-based catalysts for OER^4,10–12,33,35–40^, such a screening and comparison has not yet been rigorously carried out because of the structural complexity of the active phases. Indeed, even the atomic-scale structure of the γ-NiOOH phase itself is still unclear^4,33,38^. The lack of these atomic-scale details has, in turn, made it highly challenging to choose appropriate models for DFT-based mechanistic studies^4,38,41^. Hence, a variety of structures have been employed in the modeling, including those that resemble as-synthesized precursor phases^37^, NiO^38^, two-dimensional single layer (oxy)hydroxides^35,39^, β-MOOH analogs^4,10–12,42^, and γ-NiOOH analogs^33,40,43–45^ with or without Fe dopants. Although significant efforts have been made to explain the high activity of MFe LDHs, the diversity of studies suggests that large uncertainties exist concerning the relationship between the active site structure and the catalytic mechanism. This is because the predicted activity of the catalysts is highly sensitive to, and is an ensemble of, the geometrical structure^46,47^ and electronic structure (oxidation state)^48,49^ of the active site, as well as non-covalent interactions originating from bulk crystal structure^50,51^, the steady state of the surface configuration^52,53^, and the electronic structure methods used in the calculations^54–56^. These uncertainties, resulting from an incomplete consideration of this ensemble of factors, have hindered the mechanistic understanding of the high activity of NiFe and CoFe LDHs for the OER, which further hampers the prediction of new catalysts with improved performance.

Herein, we combine electrochemical measurements with *operando* wide-angle X-ray scattering (WAXS) and XAS data, as well as ab initio molecular dynamic simulations and a synergistic DFT approach that was benchmarked specifically for the strongly correlated Fe, Co, and Ni oxides and (oxy)hydroxides^55^, to unravel and contrast the crystal structures and electrocatalytic OER mechanisms of the active phases of NiFe and CoFe LDH catalysts. We provide the first direct atomic-scale evidence that, under OER conditions, both NiFe and CoFe LDHs transform from the as-prepared α-phase to a deprotonated γ-phase. The oxidative phase transitions are characterized by ~8% contractions in both the in-plane lattice constant and the interlayer distance, which are induced by the oxidation of Fe and M (Ni, Co), and by the anion-to-cation switching of intercalated ions, respectively. We then adopt the in-situ identified γ-phases to study the OER mechanism through DFT-based calculations. The calculated surface phase diagrams indicate that surface O sites are saturated with H by forming bridge OH, and undercoordinated metal sites are saturated with atop OH under OER conditions. These structures, and the associated reaction free energies, suggest that the OER proceeds via a Mars van Krevelen mechanism, starting with the oxidation of bridge OH at the Fe-M reaction centers (M = Ni or Co) to form O-bridged Fe-M moieties. The flexible electronic structure of the Fe site and its synergy with the nearest-neighbor M sites through the formation of the O-bridged Fe-M reaction centers fundamentally accounts for the high OER activity of MFe oxyhydroxides due to the stabilization of OER intermediates that are unfavorable on pure M-M centers and single Fe sites. Our combined *operando* experimental and DFT computational approach thus provides a consistent atomic-scale explanation for the high OER activity of the MFe LDHs.

## Results

### Electrochemical oxygen evolution and surface redox chemistry

**Fig. 1:**
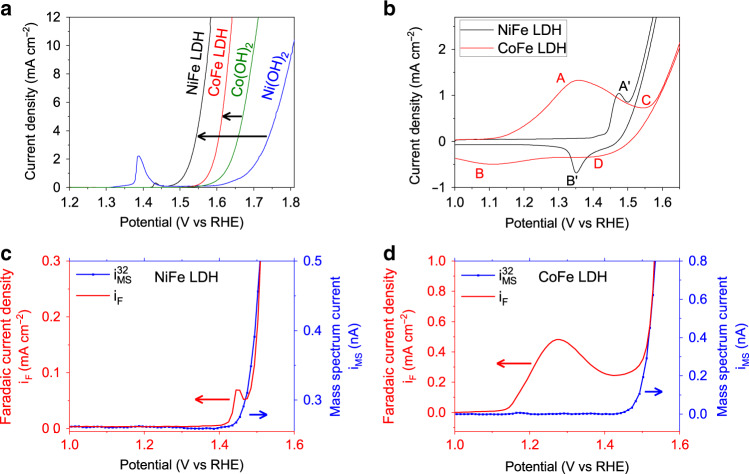
Surface chemistry and OER of NiFe and CoFe LDHs. **a** Linear sweep voltammetry of NiFe LDH (black), CoFe LDH (red), β-Ni(OH)_2_ (blue), and β-Co(OH)_2_ (green) at a scan rate of 1 mV s^-1^ in purified 0.1 M KOH by RDE (1600 r.p.m.). Catalyst loading on GC electrodes: 0.1 mg cm^−2^. **b** Stable curves obtained in cyclic voltammetry of NiFe LDH (black) and CoFe LDH (red) in 0.1 M KOH in the grazing incident cell. Redox features are indicated with capital letters. **c**, **d** Differential electrochemical mass spectrometry (DEMS) of NiFe LDH (**c**) and CoFe LDH (**d**) during a linear sweep voltammetry (LSV) in 0.1 M KOH. The faradaic current normalized by the geometric area is shown in red, whereas the mass spectrum current related to mass *m*/*z* = 32 is shown in blue.

We studied the redox chemistry of NiFe LDH and CoFe LDH (M:Fe = ~3:1) using cyclic and linear sweep voltammetry (CV and LSV) and compared their OER performance with that of their Fe-free hydroxide analogs, including β-Ni(OH)_2_ and β-Co(OH)_2_. LSV curves (Fig. 1a) indicated that OER overpotentials at 10 mA cm^−2^ are +348 mV and +404 mV for NiFe LDH and CoFe LDH, respectively, which makes them among the most active electrocatalysts in alkaline conditions. NiFe and CoFe LDHs also exhibited substantially higher catalytic activity than the hydroxides containing only Ni and Co. For NiFe LDH, the overpotential is 225 mV lower than that of NiOOH, whereas for CoFe LDH, the corresponding overpotential is 64 mV lower than that of CoOOH. We note that, although it is not an intrinsic metric, the overpotential measured at 10 mA cm^−2^ from LSV is a valid practical parameter to compare the activity trends of the catalysts^57^. This is confirmed by the good agreement with the trends of the intrinsic activity extracted with two distinct methods (see discussion in Supplementary Methods and Supplementary Figs. 1 and 2). Also, the trend of our measurement is consistent with what was reported for electrodeposited films of similar composition^7^.

The CV curves in Fig. 1b indicate that both NiFe LDH and CoFe LDH undergo redox transitions prior to (or slightly overlapping with) the onset of the OER, as confirmed by differential electrochemical mass spectrometry (DEMS) by comparing the Faraday current and the mass spectrum current related to mass *m*/*z* = 32 (Fig. 1c, d). For NiFe LDH, the Ni(II) oxidation peak at +1.47 *V*_RHE_ (A′) overlaps with the OER onset and with the corresponding reduction wave peaks at +1.35 *V*_RHE_ (B′). For CoFe LDH, the main oxidation peak at +1.35 *V*_RHE_ (A) occurs clearly prior to any OER onset. More anodically, a second and small oxidation shoulder at around +1.55 *V*_RHE_ (C) overlaps with the OER. The broad peaks B at +1.1 *V*_RHE_ and D at +1.4 *V*_RHE_ constitute the corresponding reduction waves on the cathodic scan, respectively. These redox features, in turn, provide strong evidence that the active phases for OER are not the as-synthesized phases (characterized in the Supplementary Methods and Supplementary Fig. 3).

### Tracking structural transformations during activation

**Fig. 2:**
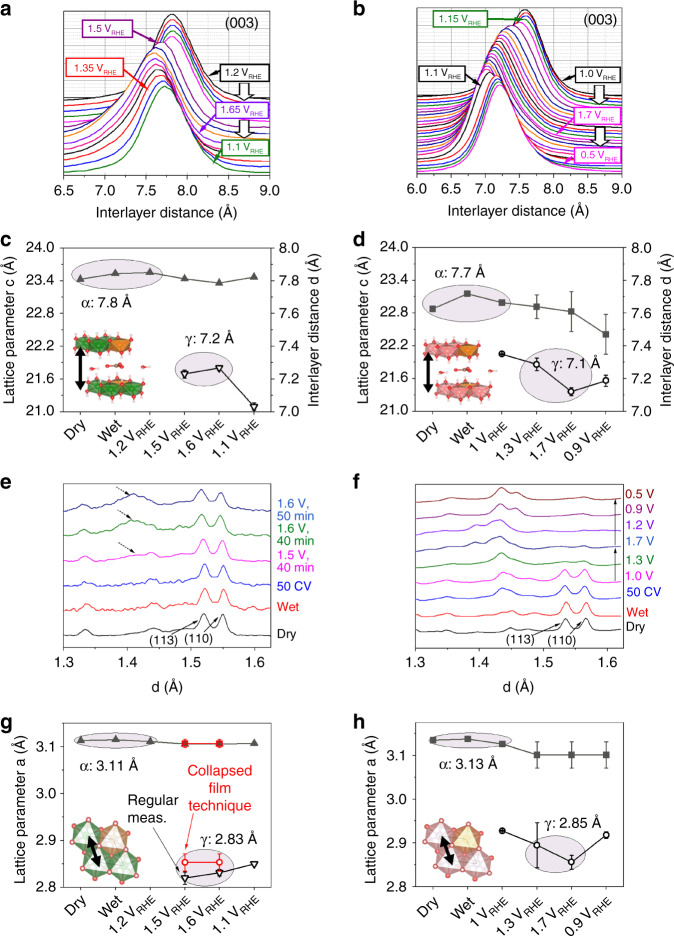
The evolution of the interlayer spacing and the intralayer metal–metal distances of NiFe and CoFe LDHs from WAXS measurement. **a**, **b** Waterfall plot of normalized and background-subtracted (003) peak obtained during in-situ WAXS in 0.1 M KOH and potential steps for NiFe LDH (**a**) and CoFe LDH (**b**). **c**, **d** Interlayer distances for NiFe LDH (**c**) and CoFe LDH (**d**) obtained by by Rietveld refinement. Full and open symbols are used for different phases. The error bars represent the SE provided by Topas. **e**, **f** In-situ WAXS patterns for *d*-values close to the (110) peak of NiFe LDH (**e**) and CoFe LDH (**f**) under various conditions. For NiFe LDH, the WAXS patterns at the reported potentials have been obtained by the collapsed film technique. In **e**, the dashed arrows point to the feature associated to the γ-phase. **g**, **h** Lattice parameter a, corresponding to the intralayer metal–metal distance in NiFe LDH (**g**) and CoFe LDH (**h**) obtained by Rietveld refinement. Full and open symbols are used for different phases. Error bars represent SD provided by Topas for the refined parameters.

To follow the phase transition of the catalysts from their as-synthesized precursor state into the catalytically active states, synchrotron-based *operando* WAXS analysis was employed. In-situ WAXS measurements were taken in 0.1 M KOH, starting from the resting state (+1 *V*_RHE_) of the catalysts, followed by stepping the applied potential up to +1.7 *V*_RHE_ and then back down to the resting state or even lower potentials. The scattering pattern was measured at the end of each step (i.e., Supplementary Fig. 4). The potential window ranges from values closely prior to the anodic wave of M(II) oxidation, reaching into the OER region and then reverting to low values to ensure the reduction to M(II). We will initially focus on the evolution of the (003) diffraction peak of the LDHs (Fig. 2a, b), which provides the characteristic interlayer distance that is absent from XAS measurements and that is central to differentiating the phases with and without intercalation of water molecules and ions. For both MFe LDHs, the evolution of the (003) peak indicates a contraction of the interlayer distance in the anodic scan and a re-expansion in the cathodic scan. The detailed interlayer distances obtained by Rietveld refinement are shown in Fig. 2c, d (additional details in Supplementary Figs. 5–9).

At the resting state and the potential before M(II) oxidation, the measured interlayer distances are 7.8 Å and 7.7 Å for NiFe and CoFe LDHs, respectively, which are typical for LDHs with intercalated water molecules and carbonate anions between the layers^23–28^. As these interlayer distances resemble that of α-Ni(OH)_2_ (~8 Å, as proposed in the Bode’s diagram^28,58^), we named this phase the α-MFe LDH. As soon as the potential increased above the M(II) oxidation potential, the (003) reflections shifted to shorter interlayer distances and a shoulder (Supplementary Fig. 9) started to develop at the interlayer distance of 7.2 Å and 7.1 Å for NiFe and CoFe LDHs, respectively. These interlayer distances are much larger than those of the anhydrous β-NiOOH (~4.8 Å)^59^ and β-CoOOH (~4.4 Å)^60^ phases but are close to that of the hydrous γ-NiOOH phase (i.e., ~7 Å)^28,59^. In analogy to γ-NiOOH and previous literature^4,24,61^, we refer to these new phases as γ-MFe LDHs.

During the cathodic scan, the interlayer distances started to re-expand as the reduction to M(II) occurred. However, the processes depended sensitively on the nature of M. For NiFe LDH, the shoulder at the interlayer distance of 7.2 Å (γ-phase) disappeared at the resting state, and the peak restored to the original value of 7.8 Å (α-phase), which indicates the reversibility of the α-to-γ transformation. Differently, for the CoFe LDH (Fig. 2b), the re-expansion to the original value (7.7 Å) is very limited under the resting state (1 V_RHE_) and is still incomplete at lower potentials (0.5 *V*_RHE_). The limited reversibility occurring in CoFe LDH has also been observed during electrochemical activation treatments (Supplementary Fig. 10) and verified by ex-situ soft X-ray XAS (sXAS) (Supplementary Figs. 11 and 12). In addition, Co-based hydroxides also have shown irreversible behavior in the literature^32^.

After clarifying the catalytically active phases under OER condition via the (003) reflection, we now turn to the (110) reflection representing the in-plane lattice constants (Fig. 2e, f and Supplementary Fig. 13). As the (110) reflection is much weaker than the (003), and broadened under OER conditions, extracting exact lattice parameters is non-trivial. Nonetheless, our Rietveld refinement revealed an unambiguous trend toward shorter metal–metal distances, from ~3.1 Å to ~2.85 Å upon α-to-γ phase transitions for both NiFe and CoFe LDHs (Fig. 2g, h). This trend agrees well with the contraction of the local metal-O and metal–metal distances in previous in-situ EXAFS measurements^3,4,12,29–32^. Thus, there are contractions on both interlayer distances and in-plane bonds upon the α-to-γ phase transition.

We note that, similar to what has been observed in previous measurements with in-situ XAS^31,62^ and Mössbauer spectroscopy on NiFe-based oxyhydroxides^63^, only a fraction of MFe LDHs in our *operando* WAXS measurements undergo phase transitions under OER potentials, although the fraction is higher for CoFe than NiFe LDH (Supplementary Fig. 9). The incomplete phase transition is likely because some nanoplates in the catalyst film are not electrochemically accessible, e.g., not in contact with the electrolyte or with the external electrical circuit (see Supplementary Information for detailed discussion and Supplementary Figs. 14 and 15). This incompleteness makes the quantitative interpretation of XAS data challenging, as the measured local structures and electronic structures are weighted averages of the two phases. Thus, the ensemble-averaged structural parameters and the electronic structure do not necessarily reflect the actual crystal structure parameters and the electronic structure of a specific phase, but strongly depend on the ratio of the α-to-γ phase transition. This issue is known for unsupported NiFe (oxy)hydroxide nanocataylsts^31^, and confirmed by our *operando* XAS (see Supplementary Methods, Supplementary Figs. 16–25, and Supplementary Tables 1–4). Therefore, what sets the present *operando* WAXS measurements apart from other ensemble-averaging approaches is their ability to probe both intrinsic local and longer-range geometric effects of specific phases, providing essential information for the identification of the active phase under OER which cannot be achieved by the experimental techniques that solely provide average local structure information. This intrinsic structural information can serve as the reference for DFT calculations to study atomic-scale geometric structures and the intrinsic electronic structure of γ-MFe LDHs, which can in turn be further employed to study the catalytic mechanism for OER. We note that, while the γ phase is the focus of the study, a consistent measurement of the α phase is important for establishing a general picture regarding the completeness and the reversibility of the phase transition.

### Geometric and electronic structures from DFT calculations

**Fig. 3:**
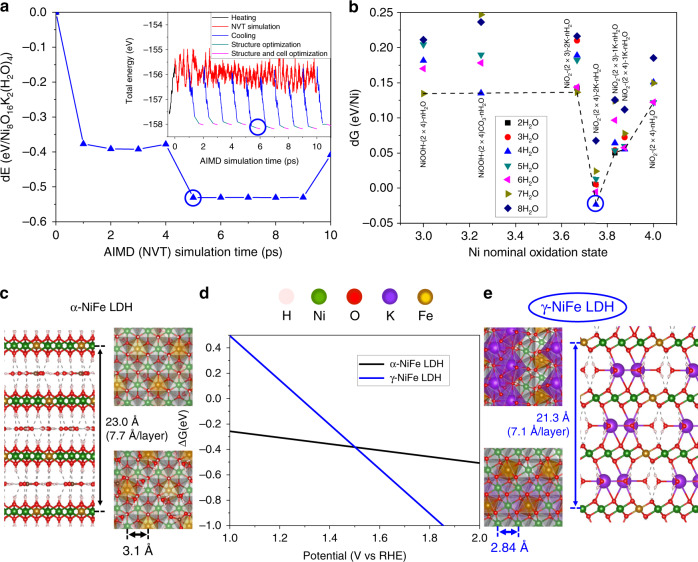
Screening process, structures, and stability (phase diagram) of NiFe LDH. **a** The relative energy of γ-NiOOH (Ni_8_O_16_K_2_·4H_2_O) at each picosecond of the AIMD simulation, which is used to screen the most stable configuration (at the 5th ps) of this specific stoichiometry. The inset is the energy evolution during the entire AIMD simulation. **b** Free energy of formation of a series of possible γ-NiOOH structures with various amounts of water and ions intercalated between the NiOOH or NiO_2_ layers. Each point is based on the most stable configuration of an AIMD simulation. For example, NiO_2_-(2 × 4)−2K-4H_2_O is from the 5th ps simulation of Ni_8_O_16_K_2_·4H_2_O in A, which is then used to study the possible configuration of γ-NiFe LDH. **c** Side, top, and bottom views of the α-NiFe LDH; **d** stability of α- and γ-NiFe LDH; **e** side, top, and bottom views of the γ-NiFe LDH. The structural parameters of α- and γ-NiFe LDH are also given.

Following the order in the above experimental section, we begin by discussing the as-prepared MFe phases (M:Fe = 3:1, α-MFe LDHs). DFT calculations indicate that α-MFe LDHs adopt the structure of hydrotalcite (Mg_6_Al_2_CO_3_(OH)_16_·4H_2_O), which is the archetypical LDH material with its characteristic three layer rhombohedral structure. Hydrotalcite formation is favorable from the component (oxy)hydroxides (FeOOH, M(OH)_2_), water (in electrolyte), and CO_2_ (in atmosphere) (Supplementary Figs. 26–28 and Supplementary Table 5), which highlights the reliability of the present calculations. In these M_6_Fe_2_CO_3_(OH)_16_·4H_2_O structures, Fe^3+^ ions are separated by M^2+^ cations within the layer, and the H_2_O and CO_3_^2−^ ions are intercalated between layers in a flat configuration, interconnected through hydrogen bonds. The intercalated species are further connected with the M_0.75_Fe_0.25_(OH)_2_ sheets by accepting hydrogen bonds from the OH terminations of the sheets (see Fig. 3). The calculated interlayer distances are 7.7 Å for both NiFe and CoFe LDHs, which is fully consistent with the measured distances of 7.7 Å−7.8 Å. The calculated in-plane lattice constants are 3.10 Å and 3.15 Å for NiFe and CoFe LDHs, respectively, which also fully agree with the measured WAXS values (3.11 Å and 3.13 Å, respectively; Fig. 2).

To identify the catalytically active phases of MFe LDHs under OER conditions, we first calculated a series of structures and configurations of γ-NiOOH with seven possible nominal oxidation states of Ni, varying from 3+ to 4+, and various amounts of water molecules and ions intercalated through ab-initio molecular dynamics (AIMD) simulations (see Fig. 3a, b). We then used the most plausible γ-NiOOH as the basis to study the possible configuration of γ-MFe LDHs. Among the structures considered, a phase with 4 water molecules and 2K^+^ cations intercalated between M_6_Fe_2_O_16_ layers is the most plausible phase under OER conditions. This conclusion is suggested by the favorable formation energies from its components ((hydroxy)oxides, water, and cations in the electrolyte, see Supplementary Table 5), and by the stability under OER conditions (see Fig. 3). The interlayer distances and the in-plane lattice constants are 7.18 Å and 2.84 Å, respectively, for both γ-type NiFe and CoFe LDH phases. These values are in excellent agreement with the measured values during OER of the MFe γ-phases: 7.1-7.2 Å and ~2.85 Å, respectively. We note that the anhydrous phases with similar overall oxidation state (M_0.75_Fe_0.25_OOH_0.25_ and M_0.75_Fe_0.25_O_2_; see Supplementary Table 6) exhibit a similar in-plane lattice constant, yet the interlayer distance is ~4.6 Å. The similarity in the in-plane lattice constants of these two distinct phases strongly suggests that local metal–metal distance alone is insufficient to accurately identify the crystal phase present under OER condition. This fact underscores that for the present catalyst systems, the *operando* scattering analysis is the best technique for identifying the 3D structure of the catalytically active phases.

As described above, DFT calculations indicate that under OER conditions, MFe LDHs transform from the as-synthesized phase with the stoichiometry M_6_Fe_2_CO_3_(OH)_16_·4H_2_O to the γ-phase with the stoichiometry M_6_Fe_2_K_2_O_16_·4H_2_O. We note that, consistent with previous measurement with Raman spectroscopy^64^, there are no hydroxyl groups in M_0.75_Fe_0.25_O_2_ layers in the γ-phase. The deprotonation of the hydroxyls of the α-phase, in turn, breaks the hydrogen bonds that exist between them and the carbonate anions and makes the intercalation of the latter highly unfavorable. Thus, CO_3_^2−^ ions are expelled and K^+^ ions are intercalated from the electrolyte during the α-γ phase transition. K^+^ ions connect M_0.75_Fe_0.25_O_2_ sheets by forming O-K-O ionic bonds in the form of zigzag chains. The channels between the zigzag K^+^ chains are filled with water molecules to fully saturate the remaining oxygen atoms in the M_0.75_Fe_0.25_O_2_ layers through the formation of O-HOH^-^O hydrogen bonds (see Fig. 3). Based on the intrinsic magnetic moment^39^, M cations are in mixed 3+ and 4+ oxidation states (see Supplementary Figs. 29 and 30, and Supplementary Table 5), which is consistent with the average oxidation states in the range of 3.0–3.7 that have been reported in the literature based on XAS measurements^4,12,31,32,61,65^. It is worth noting that, for the cases of incomplete phase transition, the measured oxidation state is a weighted average of 2+, 3+, and 4+. For γ-NiFe LDH, consistent with a previous assignment based on *operando* Mössbauer spectroscopy studies^25,63,66^, Fe cations are in a 4+ oxidation state (see Supplementary Fig. 29 and Supplementary Table 5). We note that, in addition to Fe^4+^, higher Fe oxidation states have already been reported in the literature^67–69^. We will show in the reaction mechanism study below that the flexible oxidation state of the Fe site, and its synergy with M sites, are responsible for the high catalytic activity of MFe LDHs. Based on the energetics (see Fig. 3), the formation probability of the γ-phase that we screened, K_1/4_(H_2_O)_1/2_MO_2_, is over three orders of magnitude higher than the γ-NiOOH analog K_1/3_(H_2_O)_2/3_MO_2_ used in previous studies^70^. As the activity is sensitive to non-covalent interactions induced by the bulk structure and the electronic structure, in addition to the geometric structure and electronic structure of the active site, the γ-MFe phase is used in the study of the OER mechanism below. We note that, because of the characteristic stoichiometry of the layer and atomic-scale details of the intercalated species, the γ phase cannot be obtained by simply introducing various amounts of water molecules and cations into the interlayer space of the β-MOOH analogs used in the literature. Further, as we demonstrate below, correct determination of the OER mechanism requires not only an accurate treatment of the bulk catalyst structure, but also a complete consideration of all key factors that have been missed in previous models, including the geometry, oxidation states, and adsorbate coverages on the catalyst surface.

### The catalytic oxygen evolution reaction mechanism

**Fig. 4:**
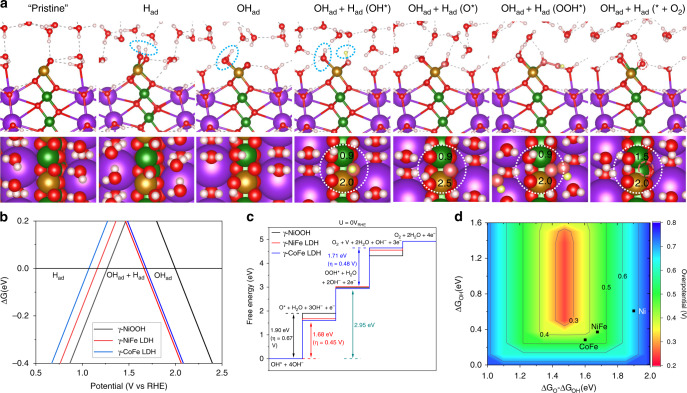
OER mechanism on the γ-phase of MFe LDHs. **a** Structures of different surface phases and OER intermediates; adsorbates of surface phases are highlighted by blue circles on the sides views, and OER intermediates are differentiated by colors (yellow instead of white for hydrogen and rose instead of red for oxygen, respectively). A dashed rose circle indicates the formation of a surface O vacancy. The reaction centers are highlighted by large white circles. The magnetic moments of Ni and Fe during OER are also given on the top views. **b** Surface phase diagram of of γ-NiOOH, γ-NiFe LDH, and γ-CoFe LDH. The representative surface phases are given in **a**. **c** Reaction free-energy diagrams for OER on γ-NiOOH, γ-NiFe LDH, and γ-CoFe LDH; the potential limiting steps and the overpotentials are also given. **d** Volcano plot of the OER overpotential as a function of Gibbs free energies of the reaction intermediates.

Beginning with the elucidated bulk structures described above, we evaluated the steady state of the (01–10) surface of γ-NiOOH, γ-NiFe LDH, and γ-CoFe LDH through surface phase diagrams, then calculated the reaction free energy diagram of oxygen redox (4OH^−^ + *←→3OH^−^ + OH* + e^−^←→2OH^−^ + O* + H_2_O + 2e^−^→OH^−^ + OOH* + H_2_O + 3e^−^→O_2_ + 2H_2_O + 4e^−^+*) (see Fig. 4, Supplementary Figs. 31–41, and Supplementary Tables 7–11). We have focused on the reaction of the surface oxygen species and neglected the potential involvement of lattice oxygen, because recent isotope experiments suggest that the latter is not favorable for these specific systems^2^. We selected the (01–10) surface, because it belongs to the family of surfaces that are exposed at the edge of catalyst sheets, and thus widely used to study the catalytic activity of layered materials^4,11,33,40,71^.

The calculated surface phase diagrams indicate that, under OER conditions, undercoordinated surface O sites are saturated with H by forming bridge OH species, and undercoordinated metal sites are saturated with atop OH when the surface is in equilibrium with the electrolyte and in steady state (see Fig. 4). Thus, we analyze the reaction free energy with a Mars van Krevelen-type mechanism, for which the reactions start from the deprotonation of the surface OH of the in-situ surface phase, instead of starting from OH adsorption, as has generally been assumed in many previous studies (to further motivate this choice, see the comparison with the reaction free energies of the conventional mechanism on two artificial surface models in the Supplementary Materials). For the Mars van Krevelen mechanism, we have found that the oxidation of two-metal coordinated bridge OH moieties is more favorable than that of one-metal coordinated atop OH due to the synergy of the two nearest-neighbor metal sites in stabilizing the potential limiting OER reaction intermediates (O* radials) by forming an O-bridged reaction center (see Fig. 4 and Supplementary Methods for details). Thus, we have focused our discussion below on the synergistic bridge OH oxidation pathway.

The highest reaction free energy barriers (Δ*G*_a_) on γ-NiOOH, γ-CoFe, and γ-NiFe oxyhydroxide surfaces are 1.90 eV, 1.71 eV, and 1.68 eV (see Fig. 4c and Supplementary Figs. 31, 33, and 38), respectively, which implies overpotentials (*η*) of 0.67 V, 0.48 V, and 0.45 V (*η* = (Δ*G*_a_ − 1.23 eV)/e). The calculated overpotentials are semi-quantitatively consistent with the present measurements at 10 mA cm^−2^, 0.57 V, 0.40 V, and 0.35 V, respectively, and with general trends in the literature. For γ-NiOOH and γ-NiFe LDH, OH* deprotonation during the OER cycle has the highest free energy barrier, which forms the potential limiting step, followed by OOH* deprotonation. On the γ-NiOOH surface, bridge OH (Ni^3+^-OH-Ni^4+^) deprotonation at 1.90 V is accompanied by Ni^3+^ oxidation to Ni^4+^, as characterized by the change of Ni magnetic moment from 1 *μ*_B_ to 0 *μ*_B_. On the other hand, on the γ-NiFe LDH surface, bridge OH (Fe^4+^-OH-Ni^3+^) deprotonation at 1.68 V is accompanied by Fe^4+^ oxidation (as characterized by the change of Fe magnetic moment, see Fig. 4), whereas the oxidation state of Ni is constant. Clearly, it is more feasible for Fe than Ni to be oxidized to a higher oxidation state, which stabilizes O* intermediates at the Fe-Ni reaction center compared with that at the Ni-Ni reaction center, and consequently lowers the free energy barrier of OH* oxidation to O*, the potential limiting step. Our calculations indicate that it is also the case for the other γ-NiFe configurations with comparable energies that could co-exist under the reaction conditions (see Supplementary Table 9).

It is worth noting that this stabilization effect is also valid and even stronger on single Fe sites as compared with single Ni sites (0.73 eV for the stabilization on single site vs. 0.22 eV for the stabilization at the Fe-Ni center), as also observed in previous studies (0.4–0.5 eV)^33,43^. However, there is a fundamental difference between the synergistic stabilization through the Fe-Ni reaction center and the stabilization through the single Fe site, with the former being over five orders of magnitude more active than the latter toward OER on NiFe LDH (see Supplementary Figs. 33 and 34). Similar synergy of two nearest-neighbor metal sites (reaction center) and flexibility of Fe site oxidation are also found on γ-CoFe LDH, for which the stabilization effect is so significant that bridge OH (Fe^4+^-OH-Co^4+^) deprotonation and the accompanied Fe^4+^ oxidation is not the potential limiting step anymore. Instead, OOH* deprotonation to O_2_(g) + vacancy (with a 1.7 eV free-energy barrier) becomes the potential limiting step. On pure Co sites of γ-CoFe LDH, OOH* deprotonation is also the potential limiting step but with higher overpotential (at 1.83 V) because of the more unfavorable O vacancy formation. Therefore, in addition to the O* intermediate, the reaction center also can stabilize O vacancies in CoFe LDH through synergy and the flexible electronic structure of Fe. However, the stabilization effect on the O vacancy formation does not seem large enough to make γ-CoFe LDH more active than γ-NiFe LDH, whereas the stabilization of O* by the introduction of Fe is beneficial in both catalysts, resulting in a small difference in activity. As a consequence, the overpotentials on NiFe and CoFe LDH are only modestly (0.14–0.22 V) higher than the optimal overpotential that is constrained by the scaling relationship (the scaling relationship of OOH* intermediate and OH* intermediate, which is 2.95 eV in the present work, leads to an optimal overpotential of 0.25 V)^72^. Such a constraint also implies that the OER overpotential of LDHs can be modestly improved by further stabilizing O* intermediates and surface O vacancies at the reaction centers simultaneously, perhaps with a more redox-flexible metal than Fe, or significantly improved by breaking the OOH* vs. OH* scaling relationship.

## Discussion

NiFe and CoFe LDHs are the archetypes of high-performing electrocatalysts for oxygen evolution in alkaline conditions. In the current work, we have identified the crystal structures of the active phase and the reaction mechanism by combining *operando* experiments, rigorous DFT calculations, and self-consistent mechanistic studies. We have found that, under applied anodic potentials, both NiFe and CoFe LDHs transform from the as-prepared α-phase to the active γ-phase. In comparison with the as-prepared phase, with an interlayer distance of 7.7 Å and an in-plane lattice constant of 3.1 Å, the catalytically active phases are characterized by a compression of both lattice spacings to 7.1 Å and 2.8 Å, respectively. These values were extracted from *operando* WAXS measurements and are also supported by DFT calculations. Although the latter is induced by the oxidation of both Fe(III) and M(II), the former is related to the swapping of intercalated ions with K^+^, which is essential in identifying the crystal structure of the active phases and cannot be accessed experimentally with other local structure-based techniques. Thus, the combination of DFT and *operando* WAXS confirms a long speculated hypothesis regarding the crystal structure of NiFe LDH under OER conditions and disprove previous assumptions of the crystal structure of CoFe LDH, while, more importantly, providing key atomic-scale details of the in-situ phases for the study of the catalytic mechanism through DFT calculations. Our calculations demonstrate that OER proceeds with a Mars van Krevelen-type mechanism on these surfaces. The flexible electronic structure of the Fe sites and their synergy with the nearest-neighbor M sites (M = Ni or Co) through forming O-bridged Fe-M reaction centers stabilize OER intermediates that are unfavorable on M-M centers and pure Fe sites. This synergistic reaction center fundamentally accounts for the experimentally observed low overpotentials of MFe for OER. The present study suggests that doping oxides with additional redox-flexible metals to form active reaction centers through the synergy with nearest-neighbor metal sites constitutes a general design principle for the synthesis of new OER catalysts design with improved catalytic performance.

## Methods

### Synthesis

NiFe LDH (Ni : Fe = 3.55:1) was synthesized by a previously reported solvothermal route in an autoclave^73^. CoFe LDH (Co:Fe = 3.33:1) was synthesized by using co-precipitation followed by a solvothermal treatment in an autoclave. Ni(OH)_2_ was synthesized using a two-step synthesis consisting of a precipitation step and a subsequent hydrothermal treatment. β-Co(OH)_2_ was synthesized by a similar process as that described by Ma et al.^74^ based on homogeneous precipitation. Further details are available in the Supplementary Information.

### RDE measurements and DEMS

RDE electrochemical experiments were performed in a three-compartment glass cell with a rotating disk electrode (RDE, 5 mm in diameter of GC, Pine Instrument) and a potentiostat (Gamry) at room temperature. A Pt-mesh and a Hydroflex reversible hydrogen electrode (RHE, Gaskatel) were used as counter electrode and reference electrode, respectively. The electrolytes were prepared with KOH pellets (semiconductor grade, 99.99% trace metals basis, Aldrich) and MilliQ water, and were further purified^75,76^. The catalyst was deposited on the GC by drop casting from an ink based on isopropanol/water solution with Nafion as a binder. The catalyst loading was 0.1 mg cm^−2^. The detailed protocol is provided in Supplementary Methods.

DEMS measurements were performed using dual thin-layer electrochemical flow cell (see Supplementary Methods for details) with nitrogen-saturated electrolyte 0.1 M KOH.

### In-situ WAXS and Rietveld refinement

The electrodes used for in-situ WAXS were prepared similarly as for the RDE measurements. A home-made grazing incident cell (Supplementary Fig. 4) based on a thin-layer concept was used with a polyether ether ketone  foil covering the top part of the cell as X-Ray window^77^. KOH (0.1 M) was used as electrolyte. In-situ WAXS experiments have been conducted at the ID31 beamline of the European Synchrotron Radiation Facility (Grenoble, France), using hard X-rays with a monochromatized beam (60–77 KeV). The electrochemical protocol consisted in keeping the sample first at the potential of 1 *V*_RHE_ after electrolyte injection (wet condition), recording electrochemical impedance spectroscopy, conducting an activation procedure by CV, and potential steps of ~10 min for the regular measurements (40 min for collapsed film technique explained in the in-situ WAXS section in the Supplementary Methods) from resting state, before the M(II) oxidation (M = Ni or Co), to OER potentials and back in the cathodic direction well below the reduction potential to M(II). The (003) and (110) peaks were fitted by Pseudo-Voigt functions in the preliminary analysis, after background subtraction. Rietveld refinement was performed on selected potentials. The hydrotalcite structure with space group R-3m was used as a model for both the LDH materials and for both the as-prepared and oxidized phases. For full details, see the Supplementary Information.

### *Operando* XAS

*Operando* XAS measurements were performed at the BL22 CLAESS beamline at ALBA light source (Barcelona, Spain) in fluorescence mode using a silicon drift diode detector. A home-made electrochemical cell was employed. A platinum mesh and leak-free Ag/AgCl electrode were used as counter and reference electrode, respectively. The powder samples were deposited on graphite paper discs (Toray Carbon Paper TP-060, Quintech) by filtration from a slurry of the sample in ethanol containing Nafion (0.1 v/v %) as a binding agent. The paper discs were mounted in the *operando* cell so that the unmodified side was facing out, whereas the side containing the catalyst layer was in contact with the electrolyte. The electrochemical conditions were identical to those described for in-situ WAXS measurements.

### DFT calculation parameters

Self-consistent, periodic DFT calculations were performed with the projected augmented wave method, as implemented in the Vienna Ab-initio Simulation Package. To generate highly accurate electrochemical stability diagrams, we employ a recently developed approach^55^, which includes the use of a Hubbard U term, a van der Waals functional (optPBE)^78^, and the use of a water-based reference state for the calculations. *U*-values, which are applied to *d*-orbitals of Fe, Co, and Ni are taken as 2.56, 3.50, and 5.20 eV, respectively. For cell shape and volume relaxations of (hydroxy)oxide compounds, a cutoff energy of 500 eV is used for the planewave expansion. For the calculations that do not involve cell optimization, a cutoff energy of 400 eV is employed. Monkhorst–Pack **k**-point grids are used for Brillouin zone integration. A (2 × 4 × 1) and a (2 × 4 × 3) **k**-point grid are employed for α- and γ-phase of LDH with R3 and R1 symmetry, respectively. For the other bulk and surface calculations, equivalent or denser **k**-point grids are utilized. An orthorhombic box (14 × 15 × 16) Å^3^ and a single **k**-point (0.25, 0.25, 0.25) for the Brillouin zone sampling are used for gas phase species. The equilibrium geometries are obtained when the maximum atomic forces are smaller than 0.01 eV/Å and when a total energy convergence of 10^−5^ eV is achieved for the electronic self-consistent field loop. AIMD simulations are performed at 400 K and quenched down to 0 K every 1 ps with a total simulation time of 10 ps. To evaluate the solvation energy of OER intermediates (see Supplementary Table 11), vacuum between the slab and the images is filled with liquid water with a thickness that is equivalent to five water bilayers. Then AIMD simulations are performed with the same protocols and time scales as that described above.

## Supplementary information

Supplementary Information

Supplementary CIF files

## Data Availability

The data supporting the findings of this study are available within this Article and its Supplementary Information files, or from the corresponding author upon reasonable request. The Supplementary Information contains descriptions of methods, discussions on physicochemical characterization of as-prepared MFe LDH, intermediate phases, size of coherently scattering domains, *operando* XAS, ex-situ sXAS, and DFT calculation. It also includes Supplementary Figs. 1–41 and Supplementary Tables 1–11.
