# Supplementary material for: In-situ structure and catalytic mechanism of NiFe and CoFe layered double hydroxides during oxygen evolution
Source: Nat Commun. 2020 May 20;11:2522. doi: 10.1038/s41467-020-16237-1 (PMC7239861; doi:10.1038/s41467-020-16237-1)
Supplement: Supplementary file 1 — Supplementary Information [file 41467_2020_16237_MOESM1_ESM.pdf]

Supplementary Information

In-situ Structure and Catalytic Mechanism of NiFe and CoFe Layered Double Hydroxides during Oxygen Evolution

Dionigi et al.

Supplementary Figures

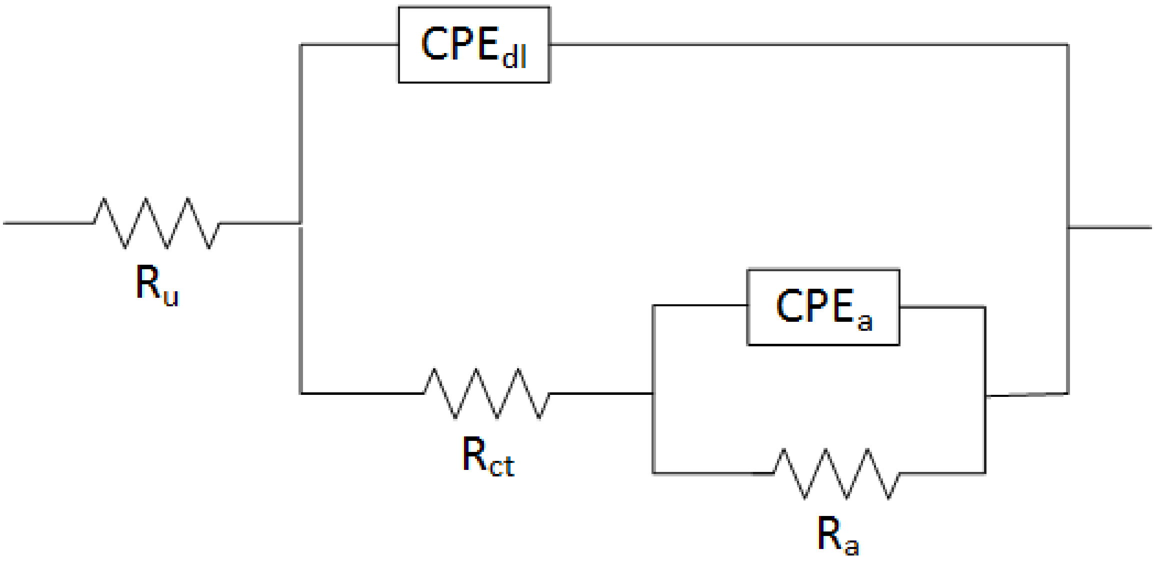

Supplementary Figure 1: Equivalent circuit scheme used to fit the impedance data. The indexes of the resistances R and the constant phase elements CPE stand for: u: uncompensated, ct: charge transfer (faradaic process), dl: diffusion layer and a: adsorbates (OER intermediates).

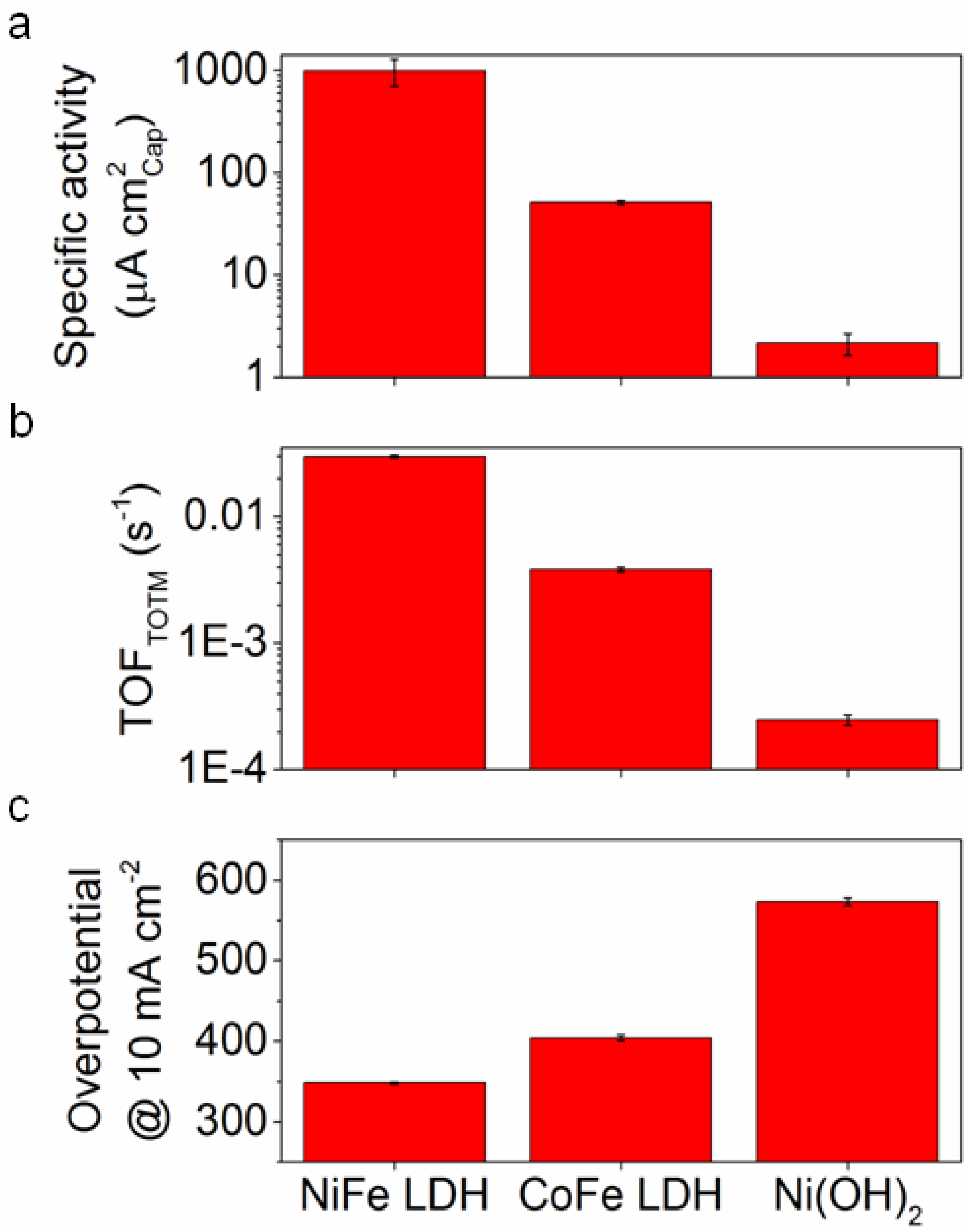

Supplementary Figure 2: Activity metrics comparison for NiFe LDH, CoFe LDH and Ni(OH)$_2$. (a) Specific activity obtained by normalizing the current at η = 350 mV by the ECSA, which in turn is calculated from the CPE$_a$ after dividing by the specific area capacitance (C$_s$) of 0.3 mF cm^{-2}. (b) Turn over frequency calculated by normalizing the current at η = 350 mV by the total number of metal sites (TOTM). (c) Overpotential at 10 mA cm^{-2}. A similar activity trend is obtained with the three different metrics.

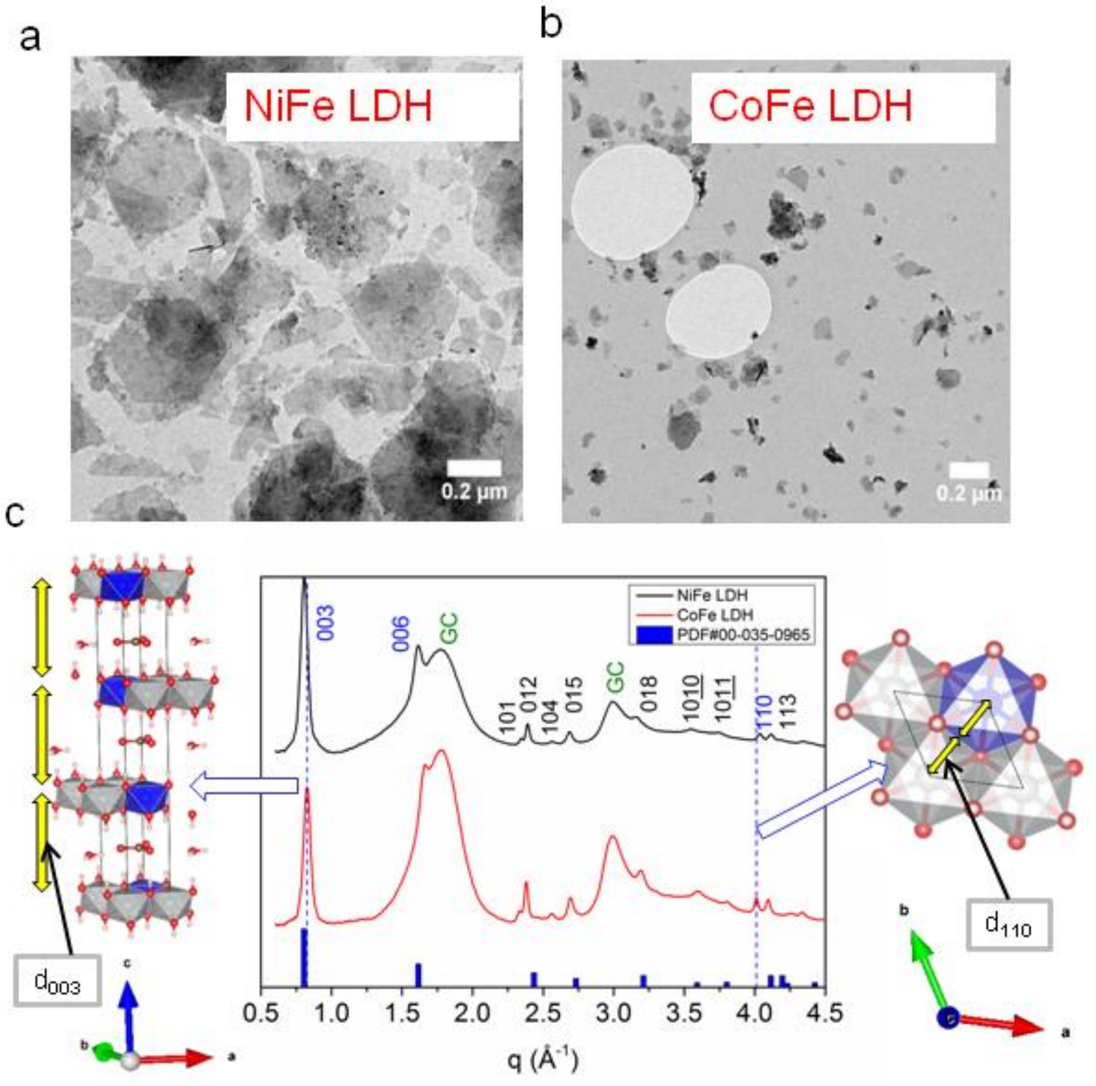

Supplementary Figure 3. Morphology and crystalline structure of as prepared NiFe and CoFe LDHs nanoplates. a, b, TEM images of as prepared NiFe LDH (a) and CoFe LDH (b) showing the nanoplatelet morphology. c, ex situ WAXS of NiFe LDH (black) and CoFe LDH (red) in dry state including 3D structural models. The hydrotalcite pattern (PDF 00-035-0965) is shown as reference. In the models, Ni and Co atoms are shown in gray, Fe in blue, oxygen in red, hydrogen in white, carbon in the carbonate anions in bronze color.

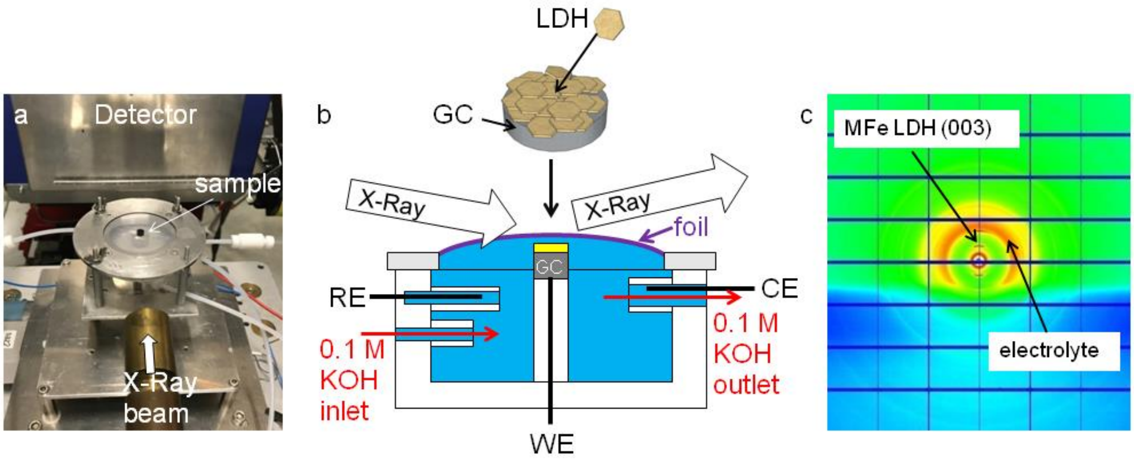

Supplementary Figure 4 In situ grazing incident cell (GID) setup. a, Photography of the GID cell mounted at ID31 at ESRF. b, Drawings showing the GID cell and its components. WE, CE and RE stand for working, counter and reference electrodes. c, Example of 2D WAXS pattern showing that the stacking of the LDH nanoplatelets (b, top) gives rise to an arc for the (003) reflection (i.e. NiFe LDH in 0.1 M KOH).

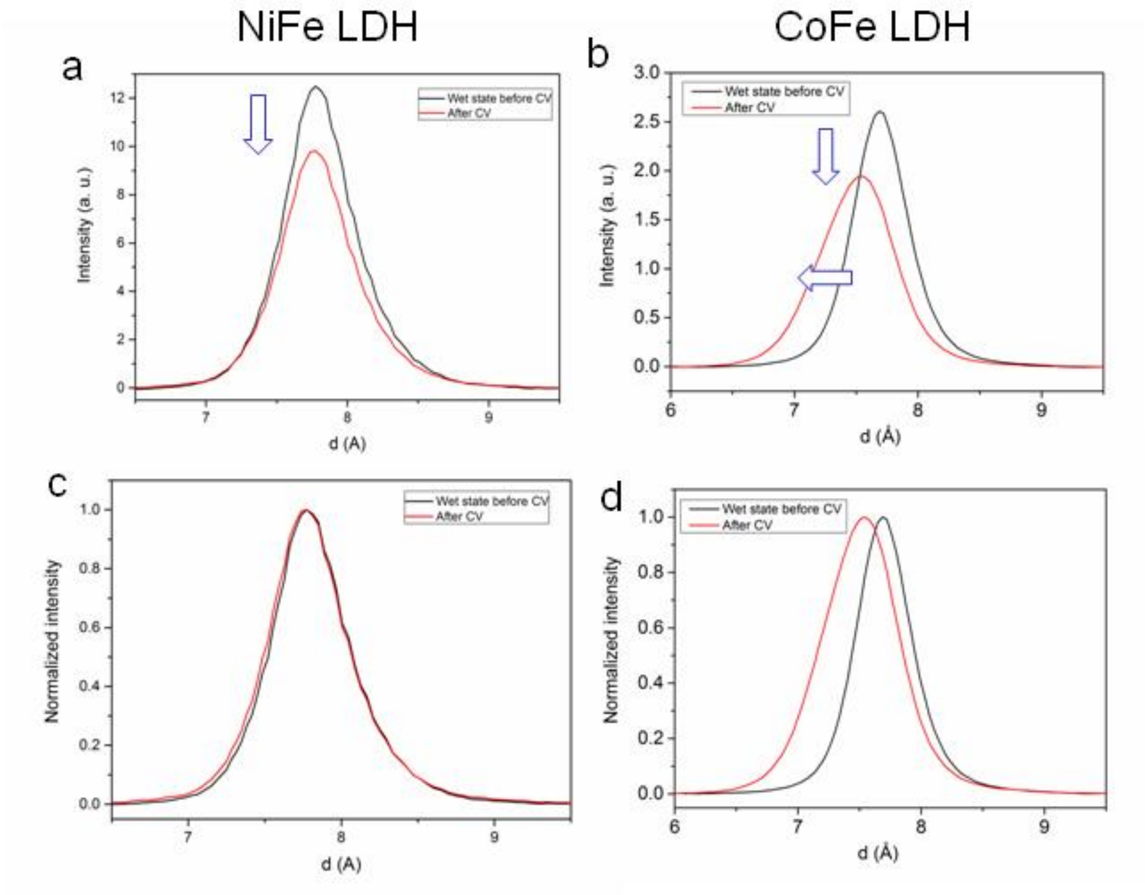

Supplementary Figure 5. in situ WAXS After Activation (Cycling Voltammetry, 50 cycles) at 1 V_{RHE}. The (003) peak of NiFe LDH (a) and CoFe LDH (b) obtained in WAXS measurements in 0.1 M KOH in wet state (black) and at 1 V_{RHE} after activation (red) consisting in 50 cycles. The loading is ~ 33 µg cm^{-2} for both catalysts. c, d, corresponding normalized peaks for the two catalysts, c for NiFe LDH and d for CoFe LDH.

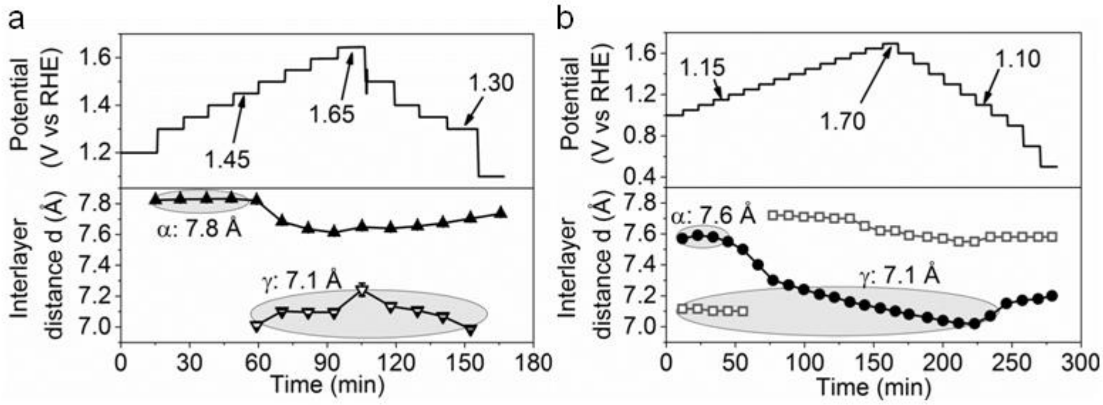

Supplementary Figure 6. Evolution of the interlayer distances for NiFe LDH and CoFe LDH in the time domain. Interlayer distances for NiFe LDH (a) and CoFe LDH (b) obtained by fitting with Pseudo-Voigt functions during potential steps experiment, after cyclic voltammetry activation. Full and open symbols are used for different fit contribution. Loadings: 20 µg cm^{-2} for NiFe LDH and 33 µg cm^{-2} for CoFe LDH. Gray shaded areas indicate potential regions associated to a particular LDH phase.

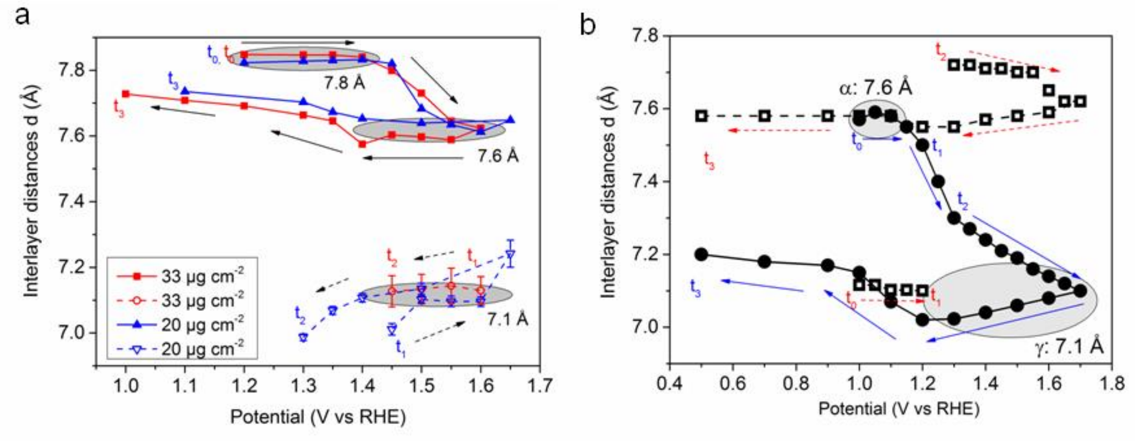

Supplementary Figure 7. Interlayer distances for NiFe LDH and CoFe LDH in the potential domain. Interlayer distances for NiFe LDH (a) and CoFe LDH (b) obtained by fitting with Pseudo-Voigt functions. Full and open symbols are used for different fit contribution. The arrows and the labels t_x indicate the directions and the time during the potential steps experiment. The error bars represent the standard error provided by the fit. Loadings: 20 µg cm^{-2} (blue) and 33 µg cm^{-2} (red) for NiFe LDH, 33 µg cm^{-2} for CoFe LDH. Gray shaded areas indicate potential regions associated to a particular LDH phase. For NiFe LDH (a), the contribution at high interlayer distance for the two loadings overlaps well, showing good reproducibility. The one at low interlayer distance associated with the γ phase also overlaps well but is more pronounced for the lower loading, so more points can be fit.

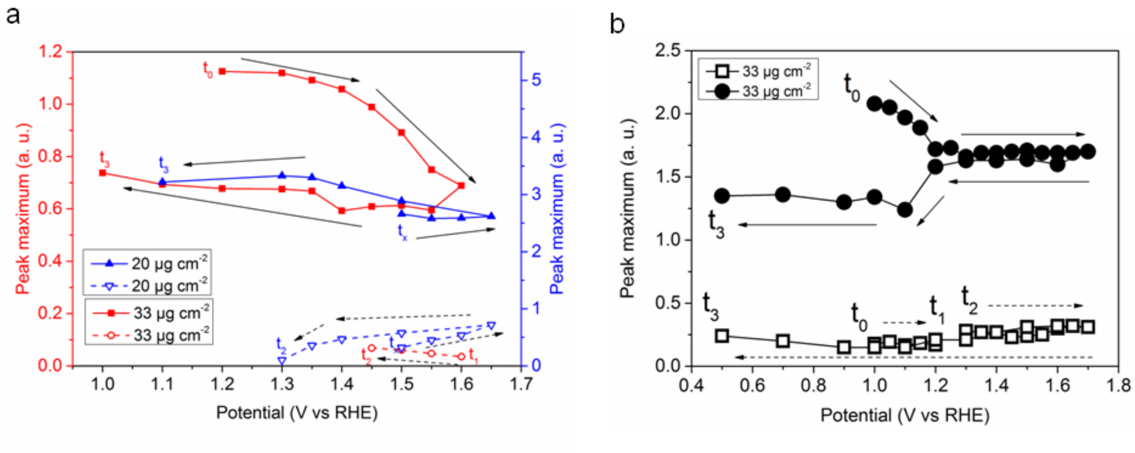

Supplementary Figure 8. In situ WAXS (003) peak maximum during potential steps in 0.1 M KOH. Peak maximum for NiFe LDH (a) and CoFe LDH (b) corresponding to the experiments shown in Figure 2 and Supplementary Figure 7. For the low loading of NiFe LDH (blue) only the data obtained for the potential steps after recontacting the sample are shown (that is after 1.5 V_{RHE}, see experimental methods for details).

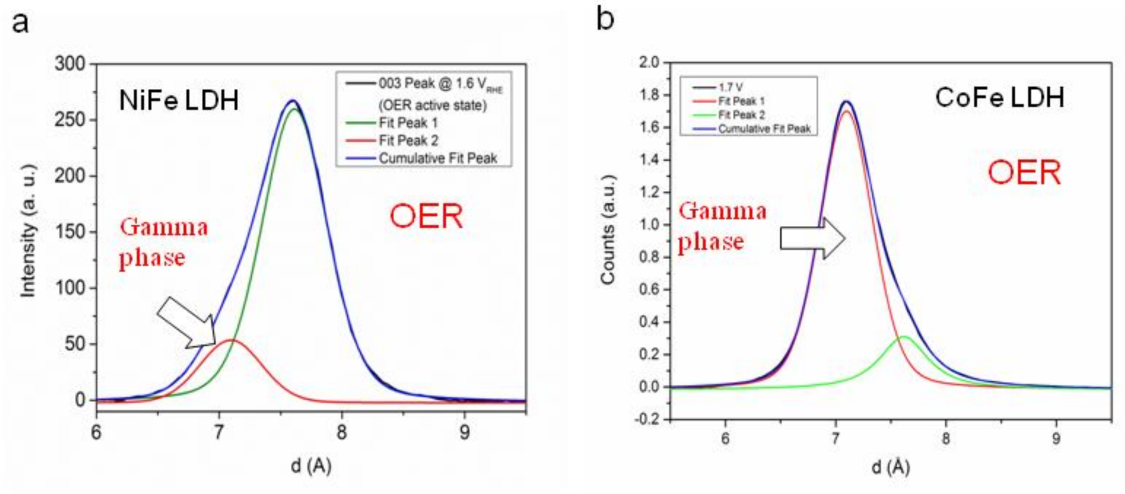

Supplementary Figure 9. OER state and Pseudo-Voigt fit. (003) peaks for NiFe LDH (a) and CoFe LDH (b) obtained by in situ WAXS in 0.1 M KOH for potentials where the OER occurs and Pseudo-Voigt fit. The contribution corresponding to the γ phase, proposed for the OER active phase, is shown in red and highlighted with an arrow.

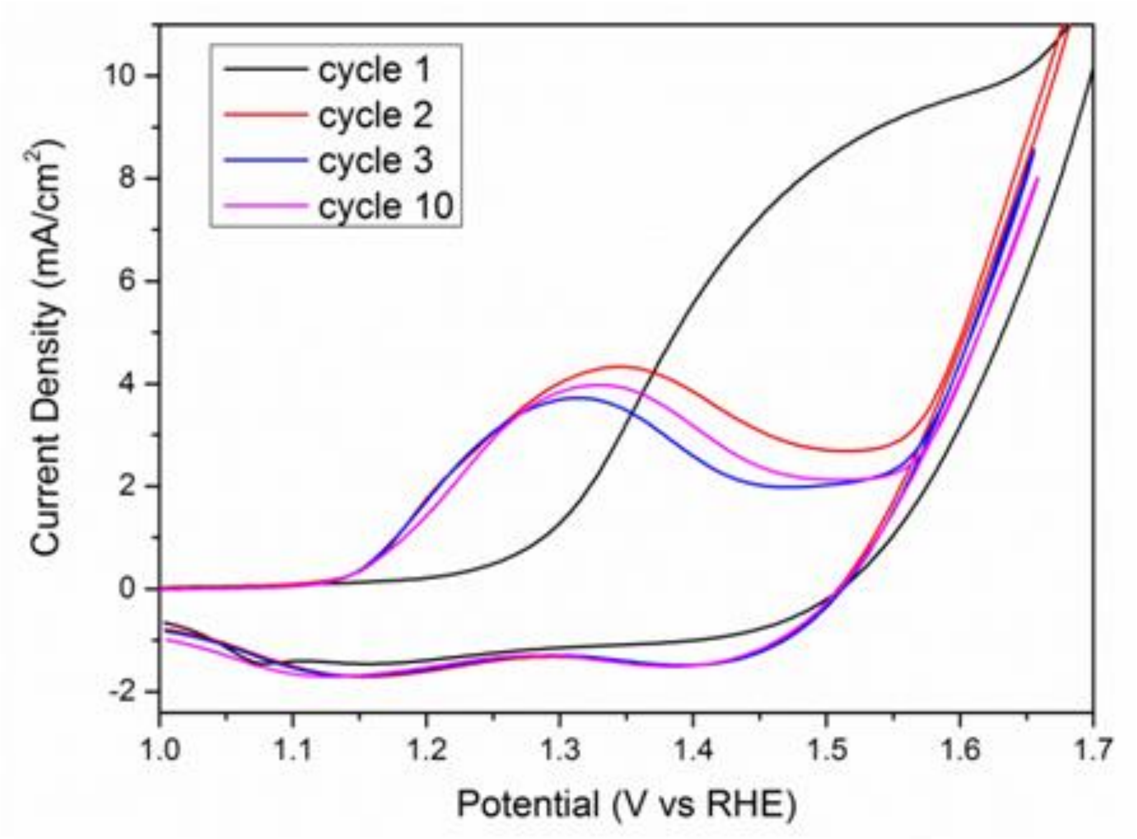

Supplementary Figure 10. First CV cycle of CoFe LDH showing irreversible oxidation. CV cycles of CoFe LDH in 0.1 M KOH obtained in the GID cell. The first cycle (black) shows a large oxidation feature. The 2nd (red), 3rd (blue) and 10th (pink), where a reasonably stable curve is obtained, are also shown for comparison.

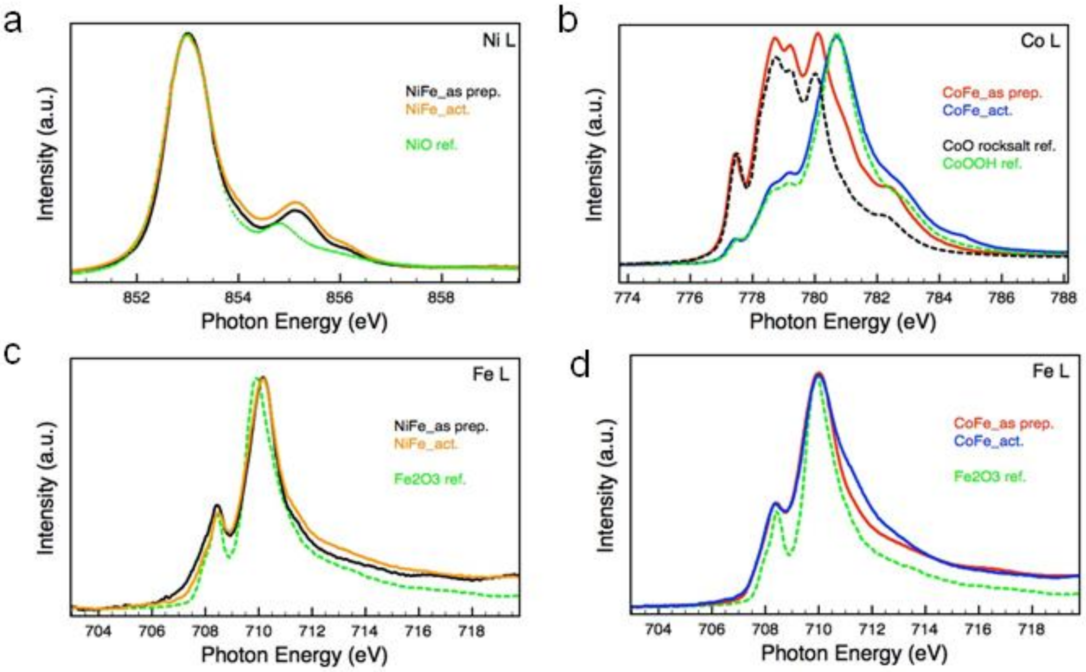

Supplementary Figure 11. *Ex situ* sXAS: Ni, Co, Fe L-edge. Ex situ soft X-ray absorption spectra of Ni L-edge (a), Co L-edge (b) and Fe L-edge (c, d) for NiFe LDH (a, c) and CoFe LDH (b, d) for as prepared conditions (black for NiFe LDH and red for CoFe LDH) and measurements after activation by cyclic voltammetry (orange for NiFe LDH and blue for CoFe LDH). Spectra obtained with NiO, CoO, CoOOH and Fe_2O_3 are shown as references (dashed lines) for Ni in +2, Co in +2, Co in +3 and Fe in +3 oxidation state, respectively.

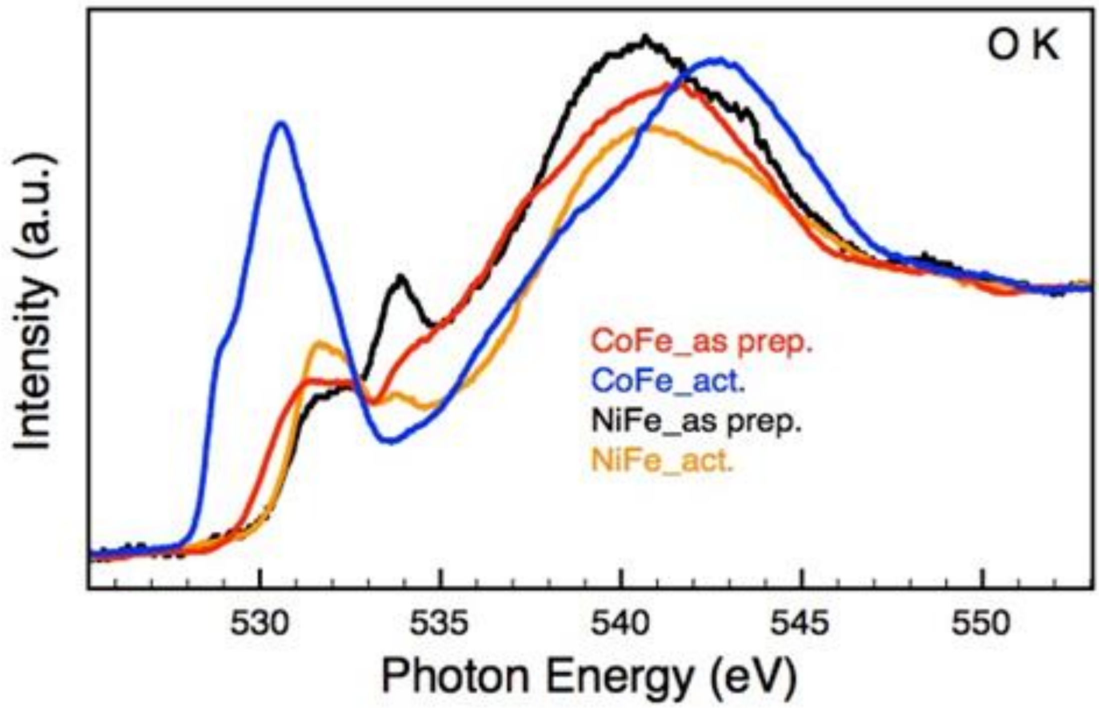

Supplementary Figure 12. *Ex situ* sXAS: O K-edge. Ex situ soft X-ray absorption spectra of O K-edge of NiFe LDH and CoFe LDH for as prepared conditions (black for NiFe LDH and red for CoFe LDH) and measurements after activation by cyclic voltammetry (orange for NiFe LDH and blue for CoFe LDH).

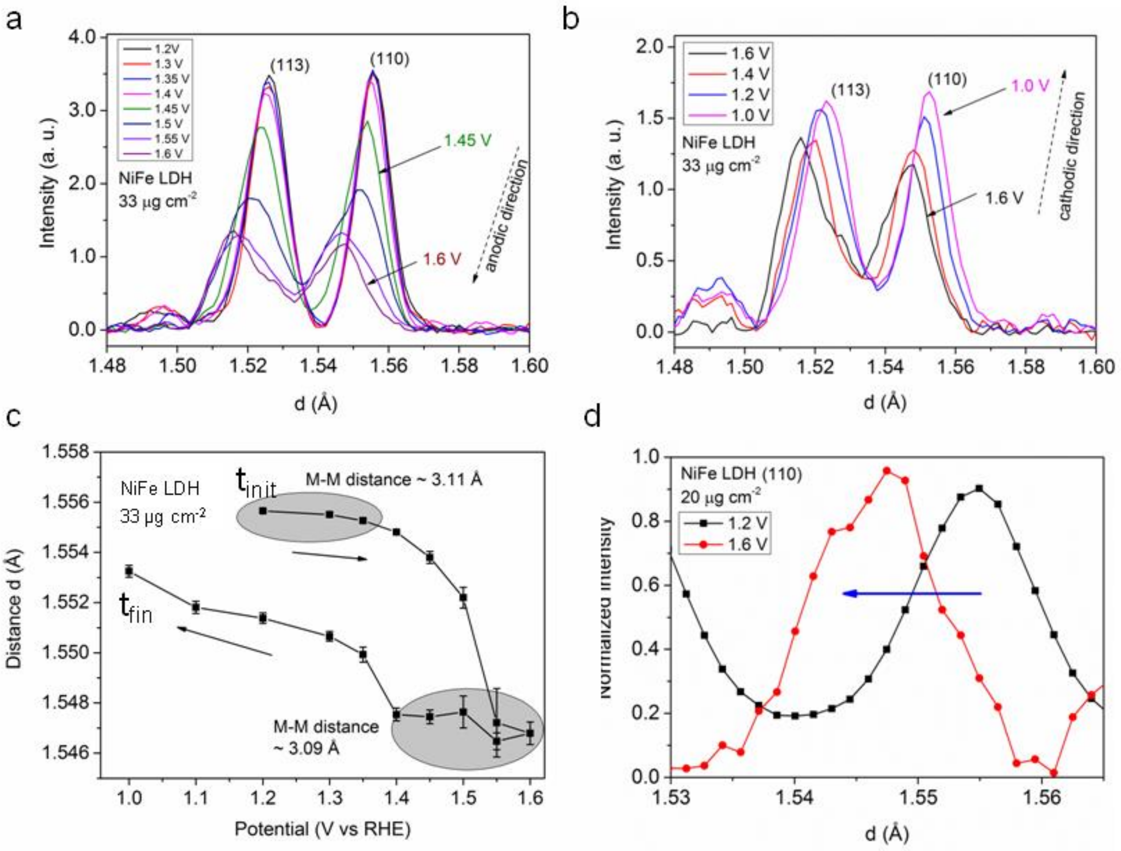

Supplementary Figure 13. (110) from in situ WAXS of NiFe LDH: metal-metal distance. a, b, *in situ* WAXS in 0.1 M KOH obtained during potential steps in the anodic (a) and cathodic (b) direction for NiFe LDH with loading of 33 µg cm^{-2}. The range of d between 1.48 and 1.60 is shown, where the (110) and (113) peaks are shown. A shift to shorter distance and following reversible re-expansion is observed and reported by the fitting analysis using a single Pseudo-Voigt function in c. This shift follows well the shift observed for the main component of the (003) peak and occur similarly at potentials corresponding to oxidation and reduction of Ni centers. The error bars represent the standard error provided by the fit. Due to this similarity, the metal-metal distances obtained of 3.11 Å and 3.09 Å are attributed to the α-NiFe LDH and α'-NiFe LDH respectively. A similar behavior was also observed for the loading of 33 µg cm^{-2} (d). The corresponding peak for γ-NiFe LDH should emerge at much shorter distances (not shown here) but it is not clearly visible without reducing the electrolyte thickness by "collapsing" the PEEK foil.

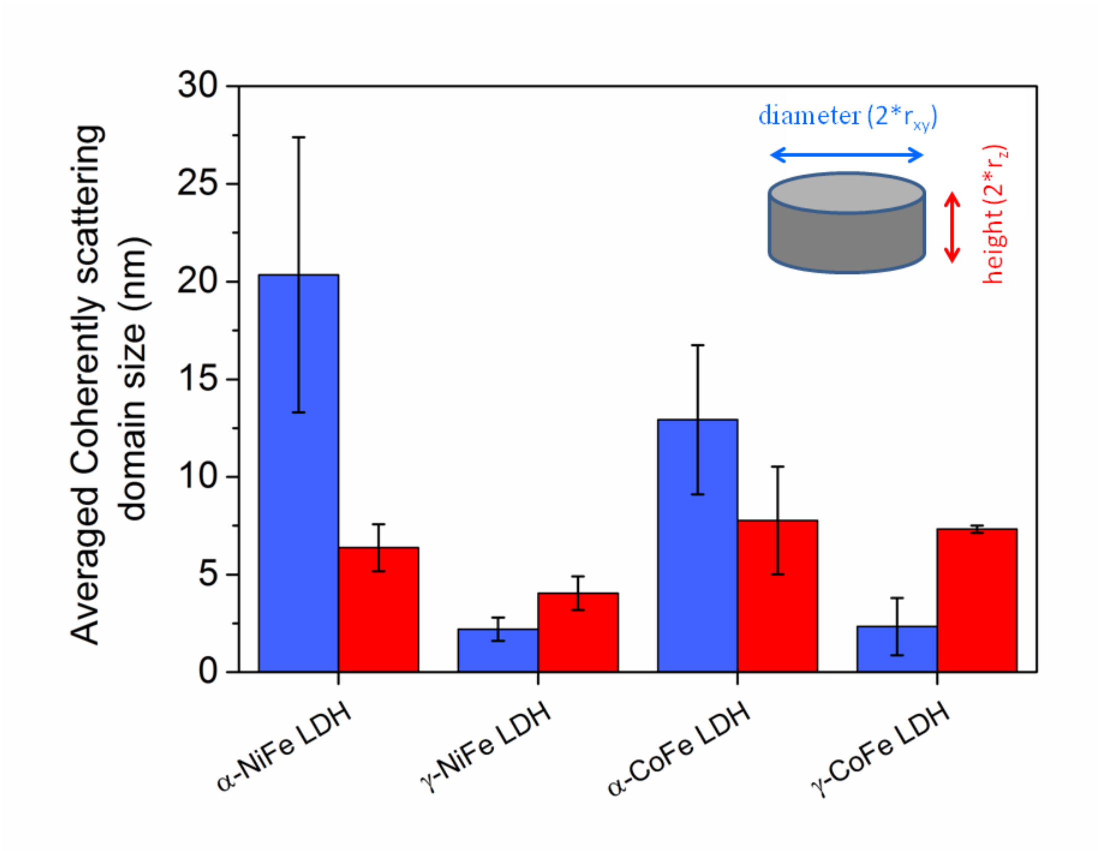

Supplementary Figure 14. Averaged coherently scattering domain size. Averaged coherently scattering domain size of NiFe LDH and CoFe LD obtained over several potentials, for the α and γ phases. In the insert the cylindrical model for the domain is shown where the size in the xy plane, the diameter (blue), and the size along the z axis, the height (red), is indicated. The error bars represent standard deviations computed in the average.

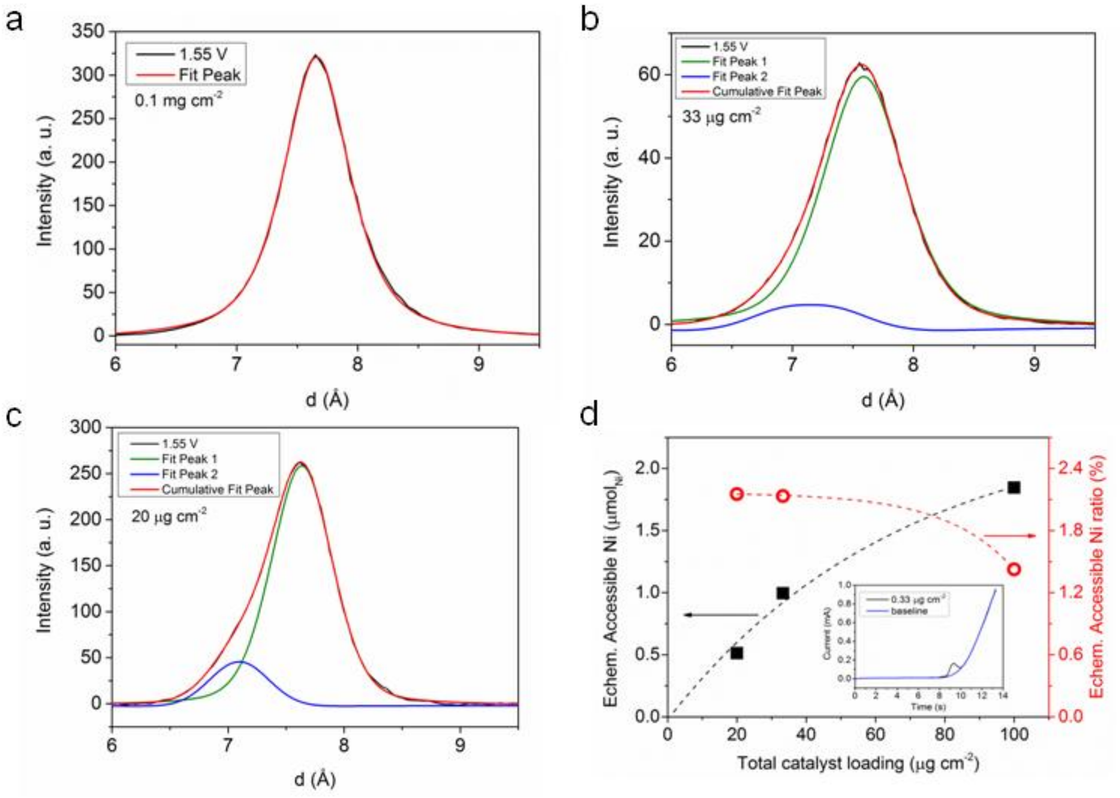

Supplementary Figure 15. Loading study with NiFe LDH: in situ WAXS during OER. a, b, c, (003) peak of NiFe LDH obtained with in situ WAXS in 0.1 M KOH during OER conditions for the catalyst loadings of 100 µg cm^{-2}(a), 33 µg cm^{-2} (b) and 20 µg cm^{-2}(c). The fit with Pseudo-Voigt functions is shown. d, Integration of the Ni(II) oxidation peak in the cyclic voltammetry (black, solid square) for the three loadings of NiFe LDH and corresponding percentage of electrochemical accessible Ni atoms (open red circle). A spline function has been used to create a baseline for the Ni(II) ox peak for the integration (example shown in blue in the insert). The percentage of electrochemical accessible Ni atoms has been obtained by calculating the Ni atoms that oxidize from the integration of the oxidation peak by assuming 1 e$^-$ per Ni atom and by normalizing this by the total Ni atom in the catalyst layer based on ICP. Dashed lines are just guides for the eye.

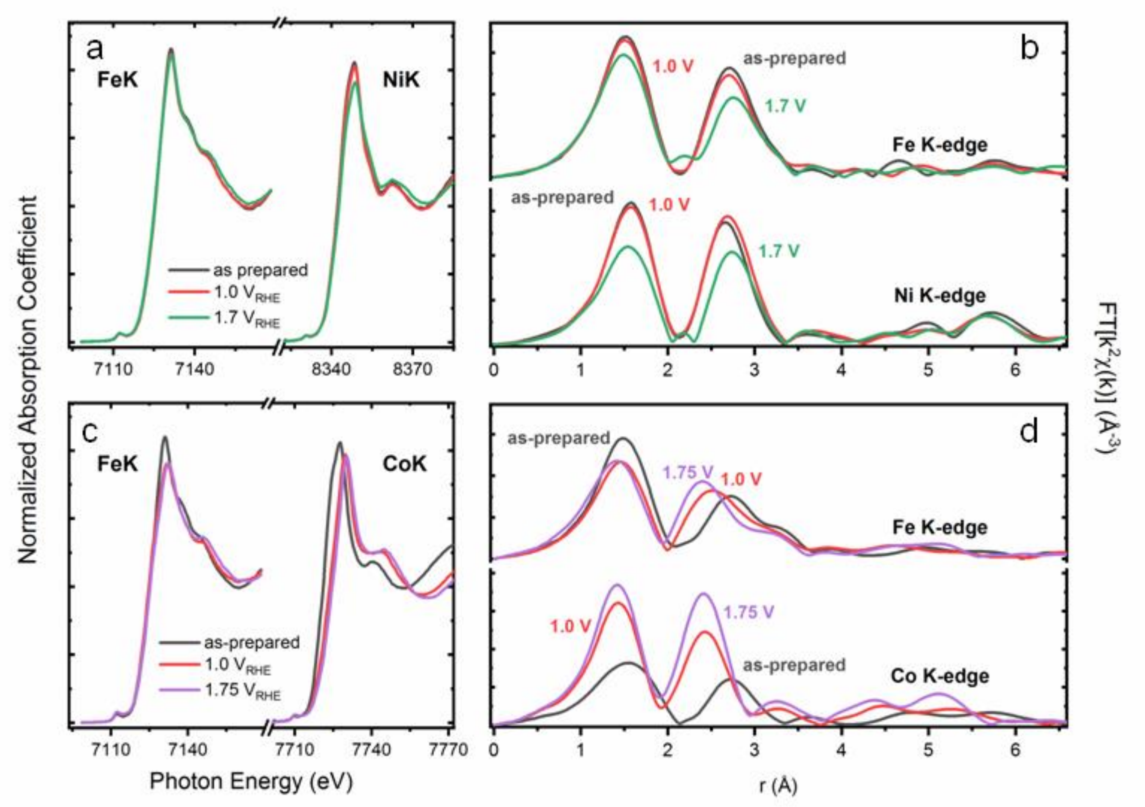

Supplementary Figure 16. XAS spectra of NiFe LDH (a, b) and CoFe LDH (c, d) samples measured under reaction conditions. (a) and (c) display XANES spectra of the Fe K-edge (a, c), Ni K-edge (a) and Co K-edge (c). (b) and (d) present EXAFS spectra at the Fe K-edge (b, d), Ni K-edge (b) and Co K-edge (d). Both XANES and EXAFS measurements indicate the larger changes (oxidative phase transition) of CoFe LDH than NiFe LDHs. Consistent with WAXS measurement, XAS spectra indicate that CoFe LDHs have a higher fraction of α-to-γ phase transition than NiFe LDHs under OER conditions.

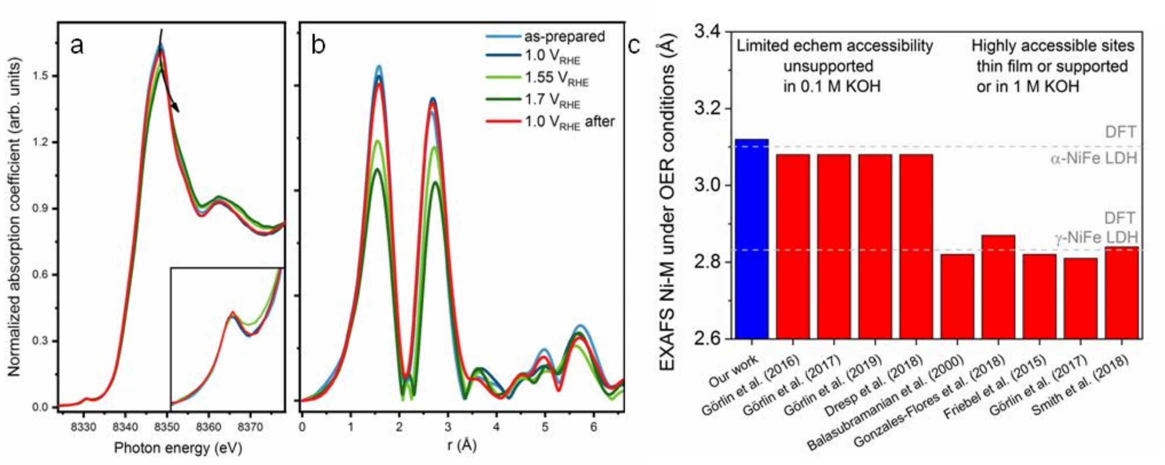

Supplementary Figure 17. Ni K-edge XAS (a-XANES, b-EXAFS) spectra of NiFe LDH as-prepared and at different potentials. C) Literature comparison of Ni-M distances under OER conditions obtained by fitting EXAFS data for NiFe-based (oxy)hydroxide catalysts. The horizontal dashed lines indicate the values expected from DFT calculations (our work) for the pure α-NiFe LDH and pure γ-NiFe LDH. The figure shows that for limited electrochemical accessibility, as with an unsupported catalyst in 0.1 M KOH, the ensemble-averaged values are not far from the pure α-NiFe LDH,[1-4] which indicated very limited phase transition, while in the cases of highly accessible sites, i.e. thin film or supported catalyst or 1 M KOH,[2,5-8] the extracted values are in agreement with the value of pure γ-NiFe LDH. It is worth to note that Görlin et al. studied both the cases in the same work.[2]

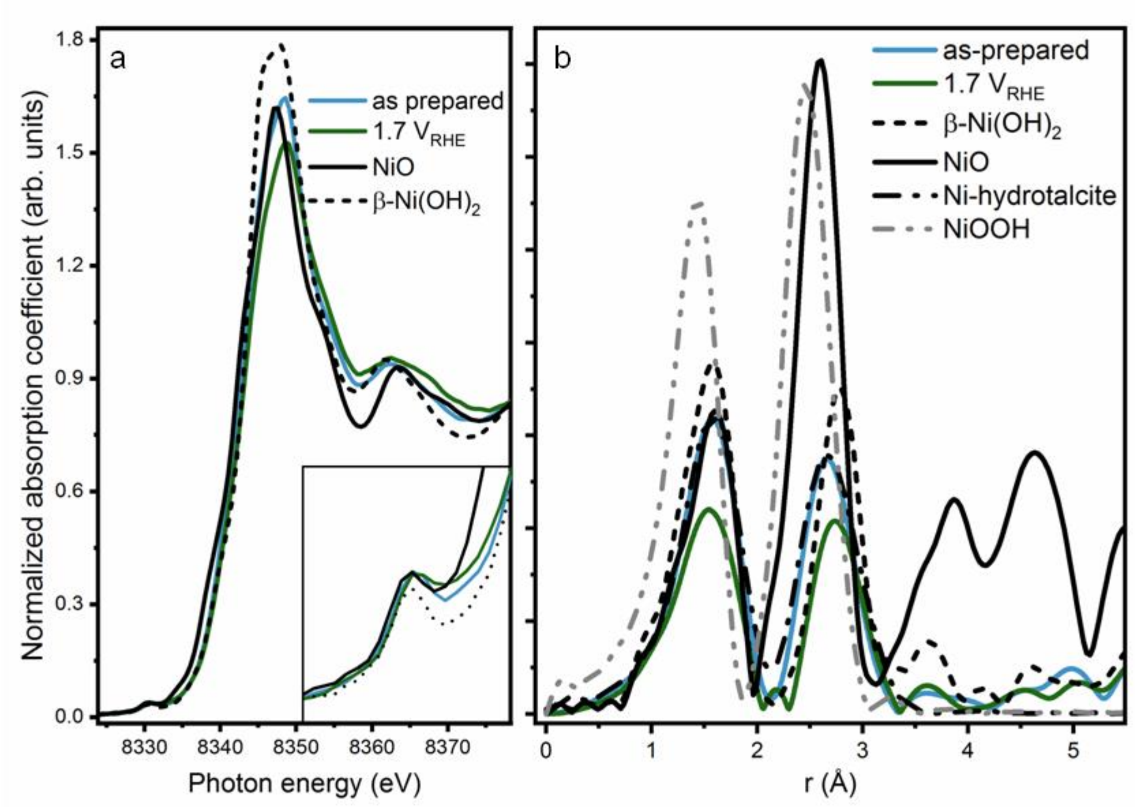

Supplementary Figure 18. Ni K-edge XAS spectra of NiFe LDH and reference compounds. a) NiK XANES spectra of NiFe LDH as-prepared and at OER potential along with experimentally measured NiO and β-$Ni(OH)_2$. b) NiK EXAFS spectra of NiFe LDH as-prepared and at OER potential compared with experimentally measured NiO and β-$Ni(OH)_2$ as well as feff modelled spectra of NiOOH and Ni occupying cationic positions in hydrotalcite structure.

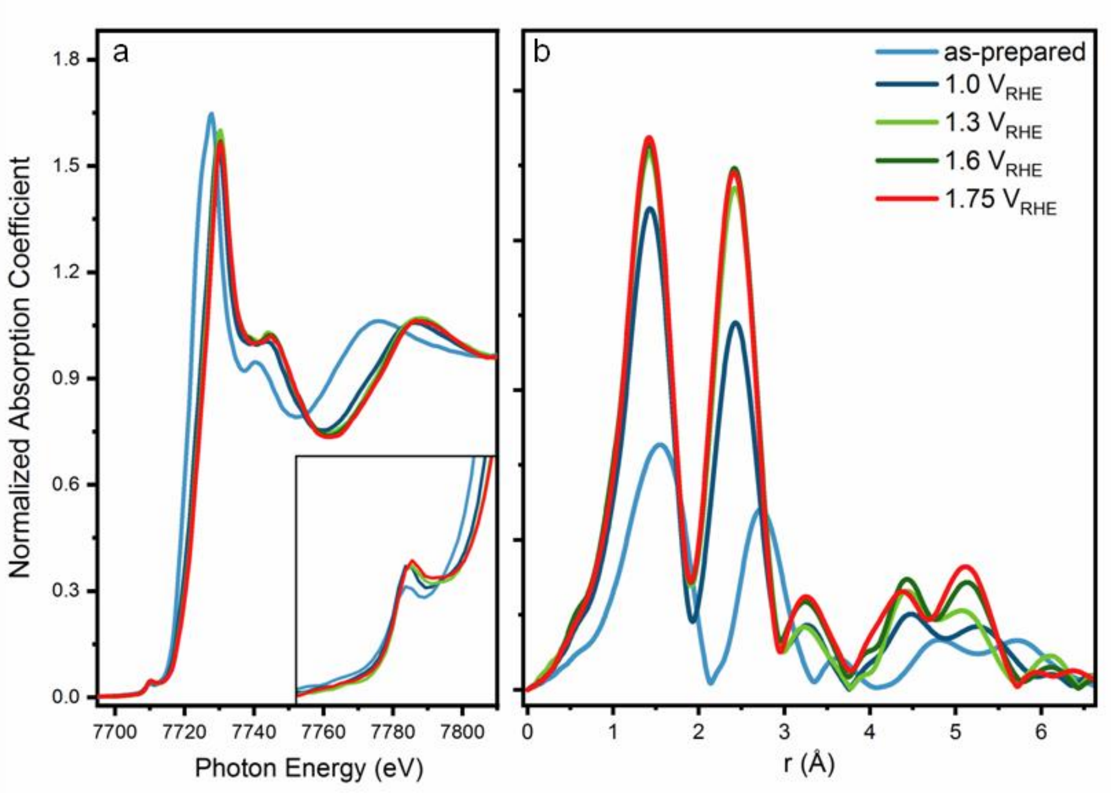

Supplementary Figure 19. Co K-edge XAS (a-XANES, b-EXAFS) spectra of CoFe LDH as-prepared and at different potentials.

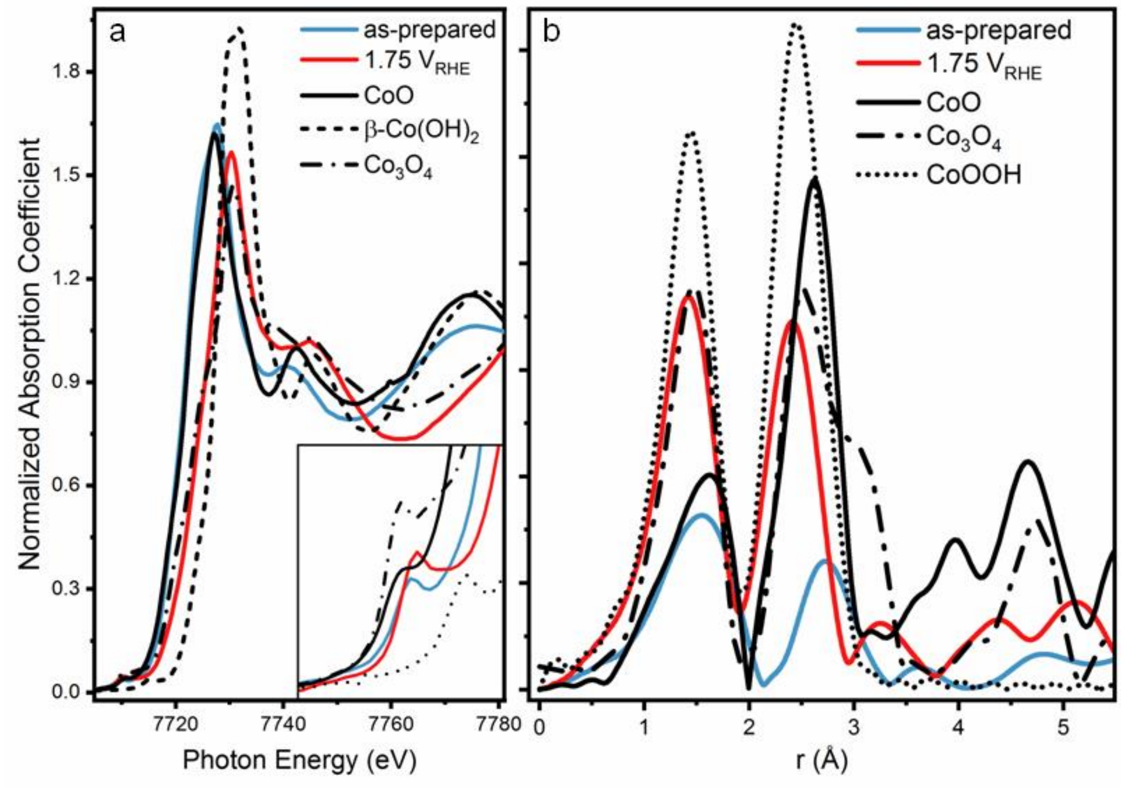

Supplementary Figure 20. Co K-edge XAS spectra of CoFe LDH and reference compounds. a) CoK XANES spectra of CoFe LDH as-prepared and at OER potential along with experimentally measured CoO, β-Co(OH)$_2$, and Co_3O_4. b) CoK EXAFS spectra of CoFe LDH as-prepared and at OER potential compared with experimentally measured CoO and Co_3O_4 as well as feff modelled spectra of CoOOH.

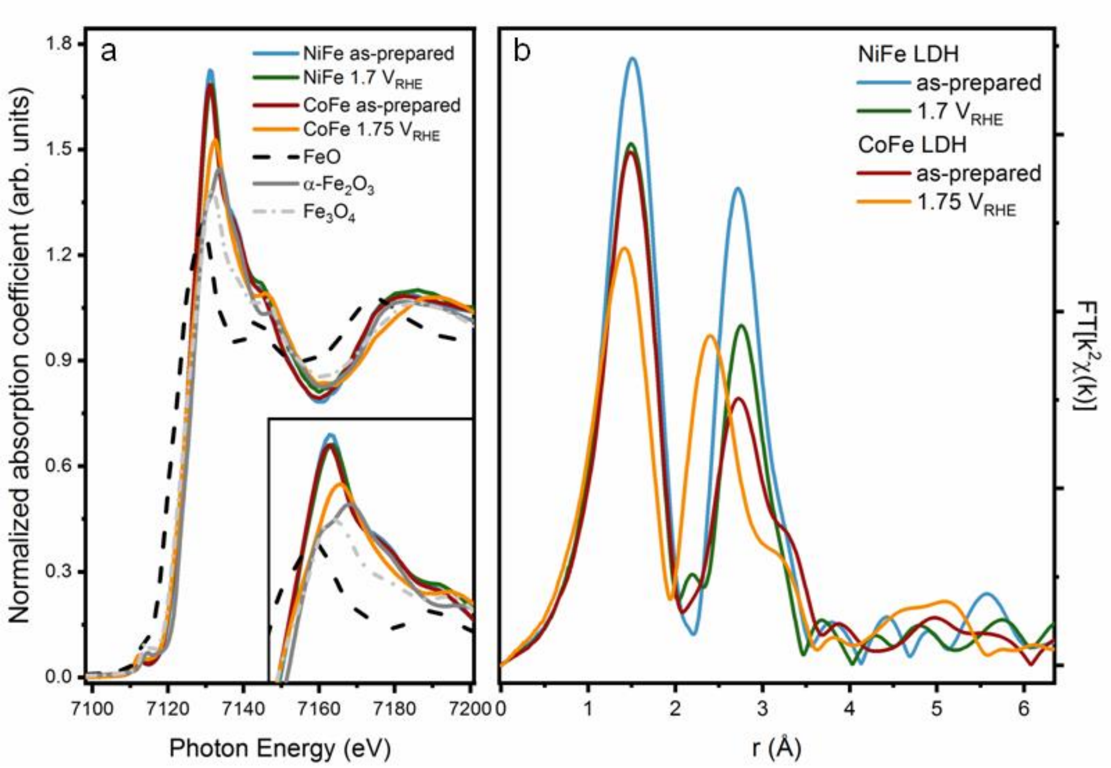

Supplementary Figure 21. Comparison of Fe K-edge XAS spectra of NiFe, CoFe LDH, and reference compounds. a) FeK XANES spectra of NiFe and CoFe LDH as-prepared and at OER potentials along with experimentally measured FeO, α-Fe_2O_3, and Fe_3O_4. b) FeK EXAFS spectra of NiFe and CoFe LDH as-prepared and at OER potentials.

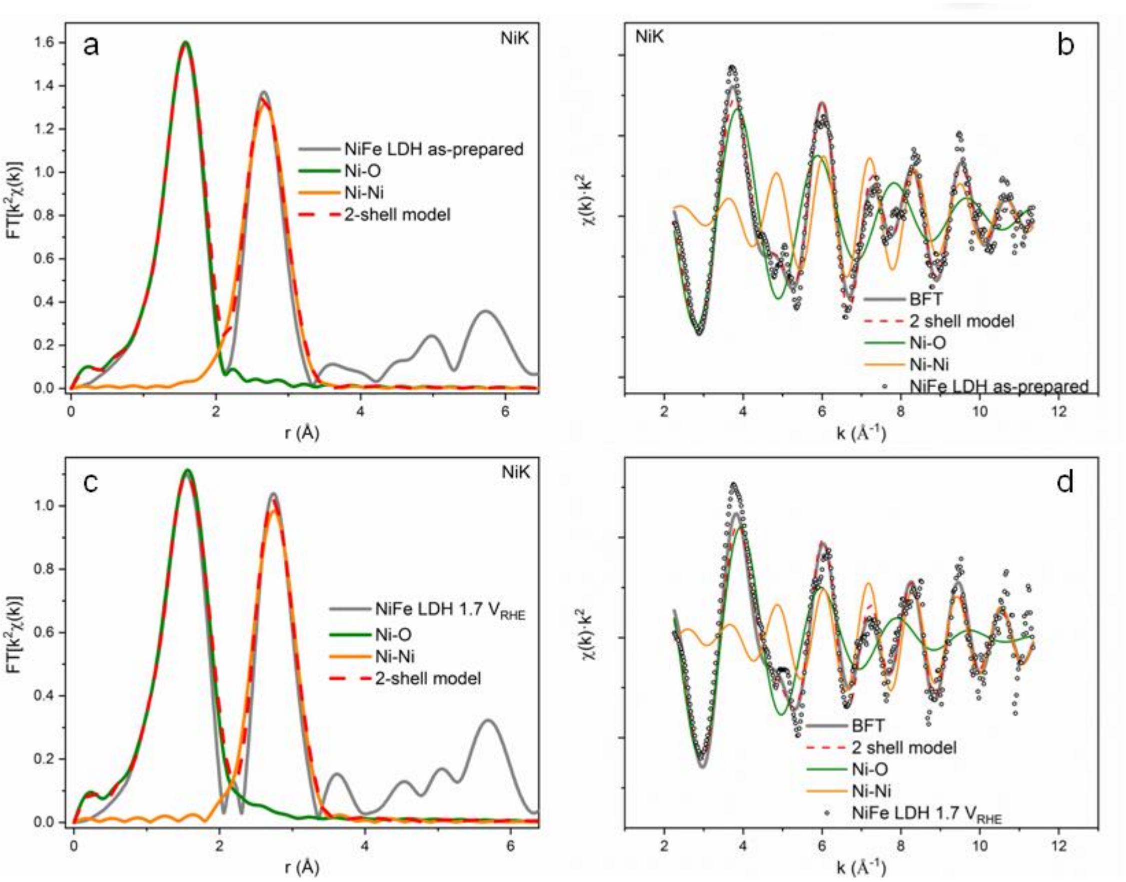

Supplementary Figure 22. Fitting of Ni K-edge EXAFS spectra of NiFe LDH as prepared and under OER conditions at 1.7 V_{RHE}. Spectra in R-space (a and c) and k-space (b and d) are shown along with the corresponding subspectral components.

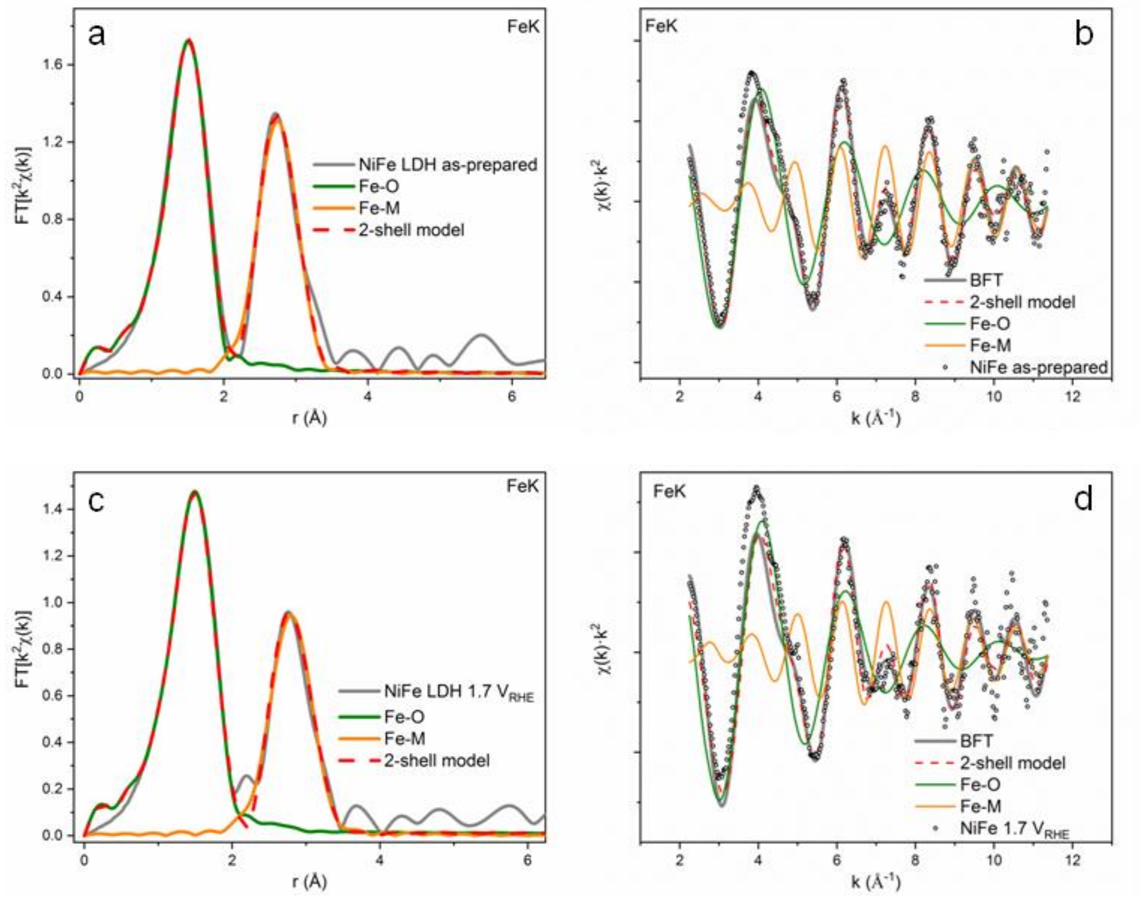

Supplementary Figure 23. Fitting of Fe K-edge EXAFS spectra of NiFe LDH as prepared and under OER conditions at 1.7 V_{RHE}. Spectra in R-space (a and c) and k-space (b and d) are shown along with the corresponding subspectral components.

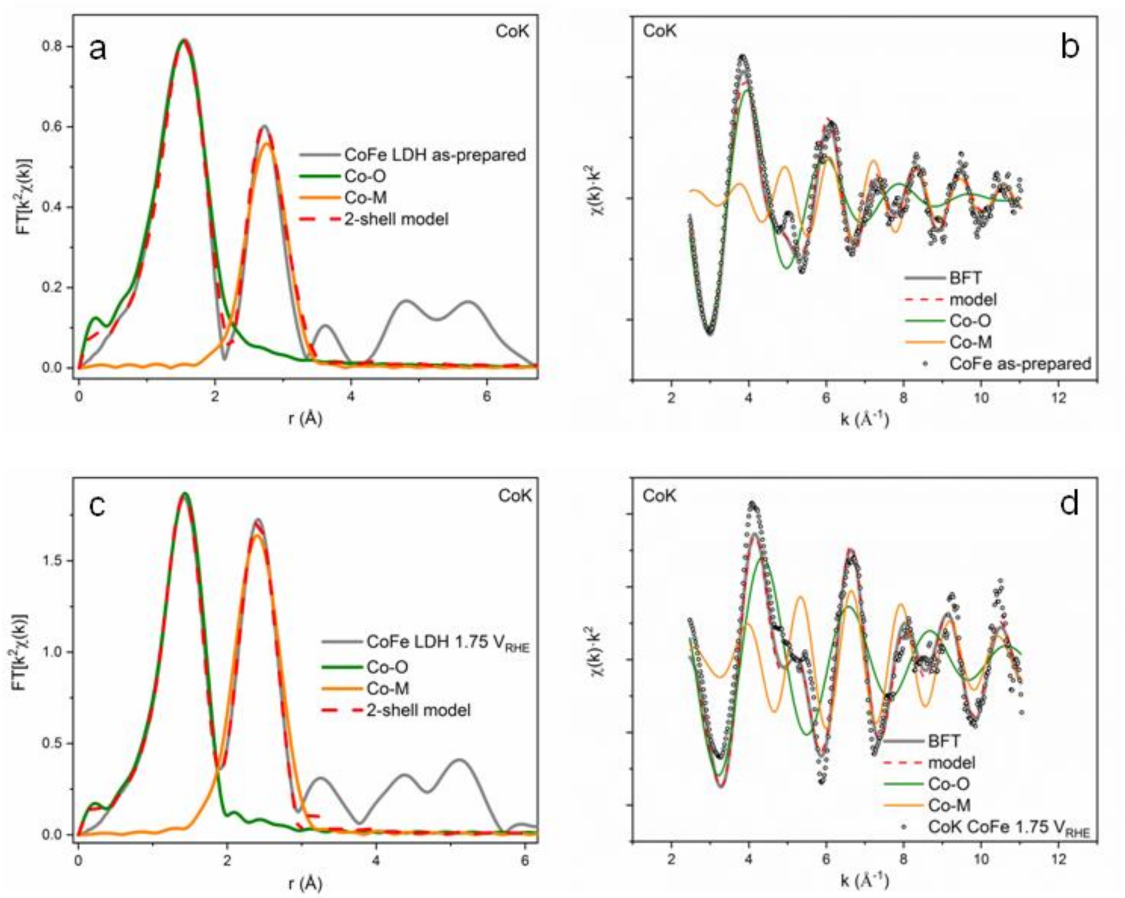

Supplementary Figure 24. Fitting of Co K-edge EXAFS spectra of CoFe LDH as prepared and under OER conditions at 1.75 V_{RHE}. Spectra in R-space (a and c) and k-space (b and d) are shown along with the corresponding subspectral components.

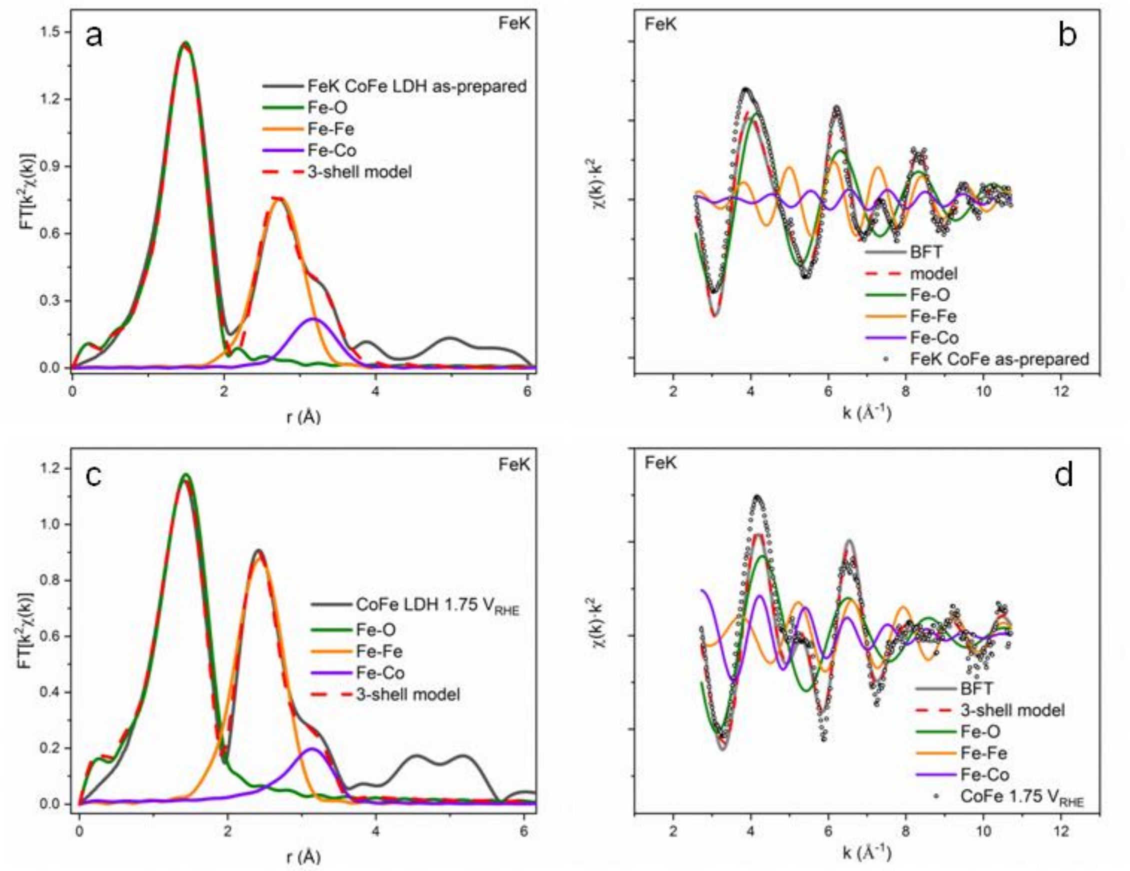

Supplementary Figure 25. Fitting of Fe K-edge EXAFS spectra of CoFe LDH as prepared and under OER conditions at 1.75 V_{RHE}. Spectra in R-space (a and c) and k-space (b and d) are shown along with the corresponding subspectral components.

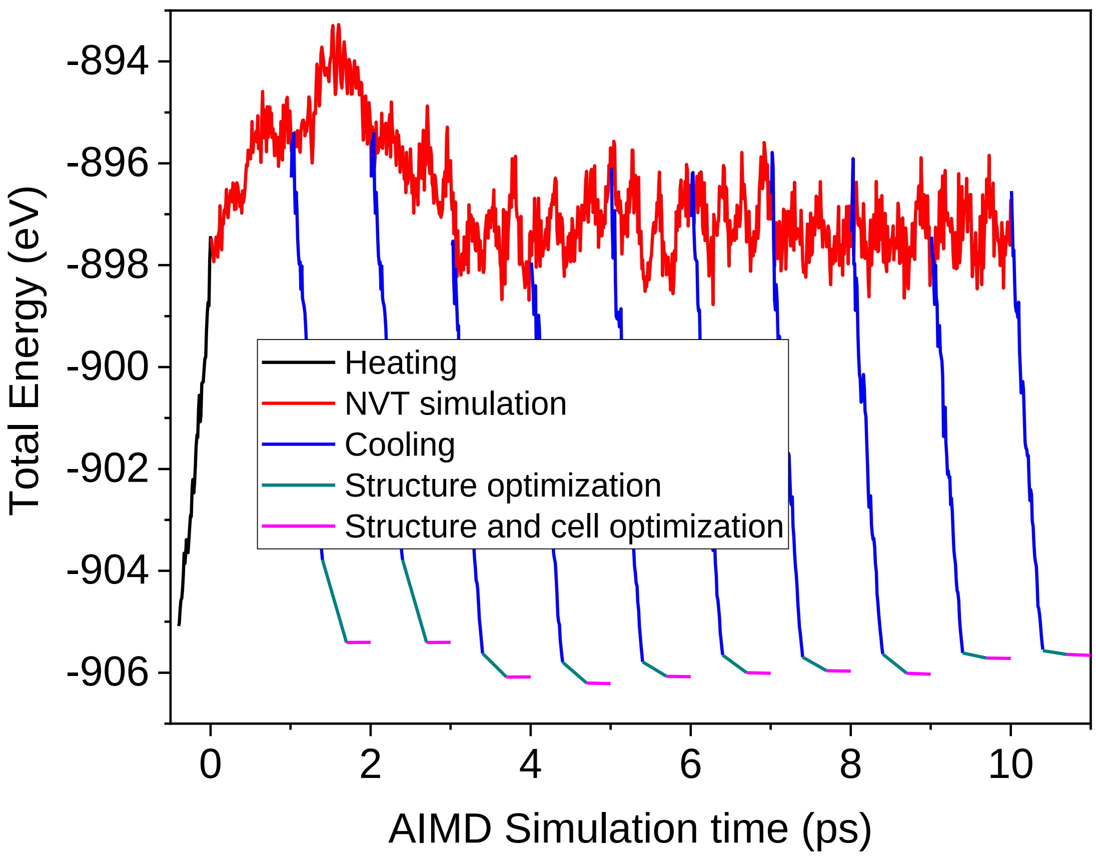

Supplementary Figure 26. The scheme of AIMD simulation used in the present work.

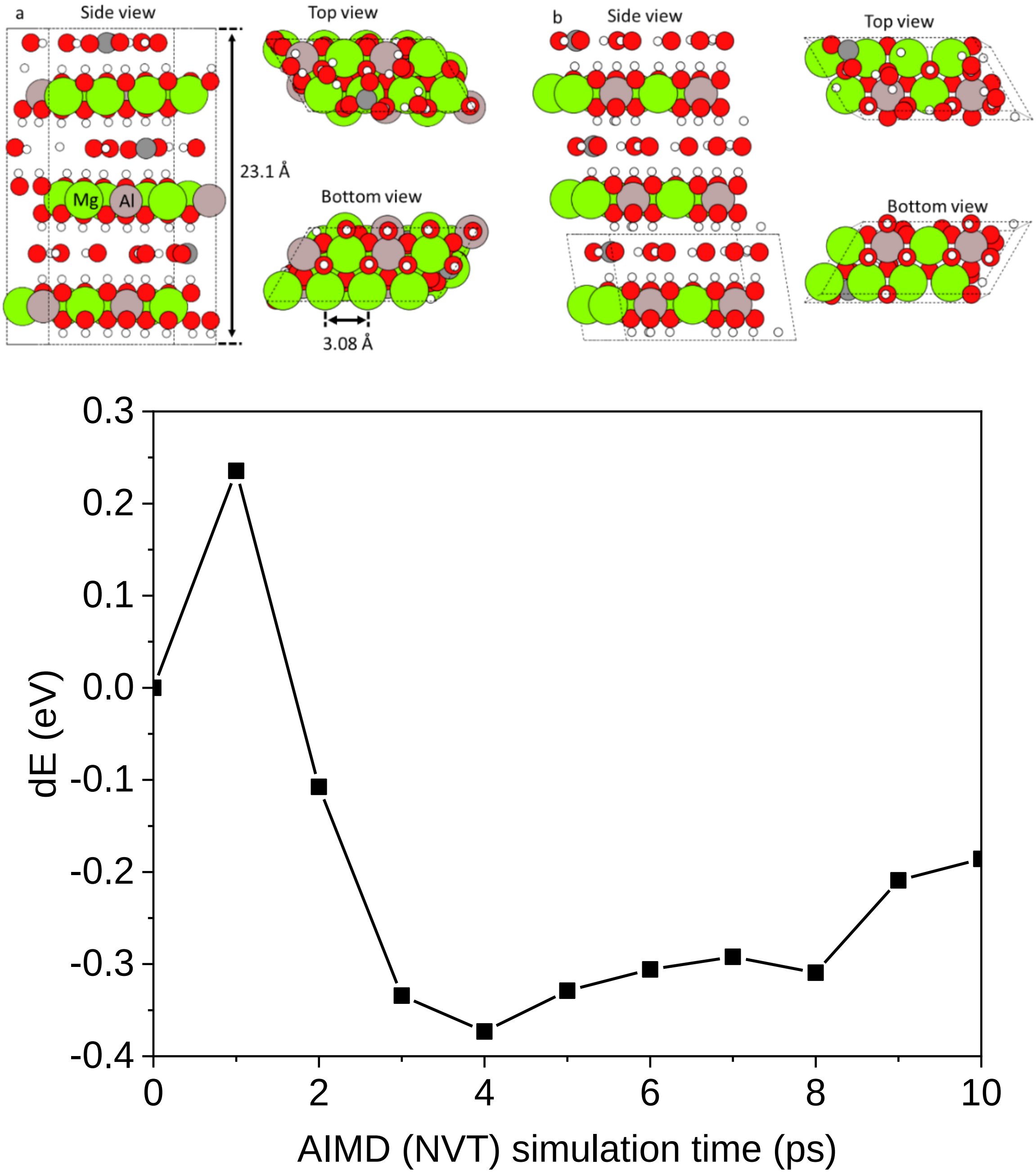

Supplementary Figure 27. Relative stability of various configurations of 3R MgAl hydrotalcite ($Mg_6Al_2CO_3(OH)_{16}\cdot 4H_2O$) from AIMD simulations. The intercalated water and ions are randomly introduced in the initial configuration. The 3R structure at the 4th ps (a) and the 1R equivalence (b) are also given. The energy difference between 3R structure and 1R equivalence is a few meV in general. The cells are indicated with dashed lines. Mg, Al, C, O and H are presented by green, brown, gray, red and while balls, respectively. The cells are indicated by dashed lines.

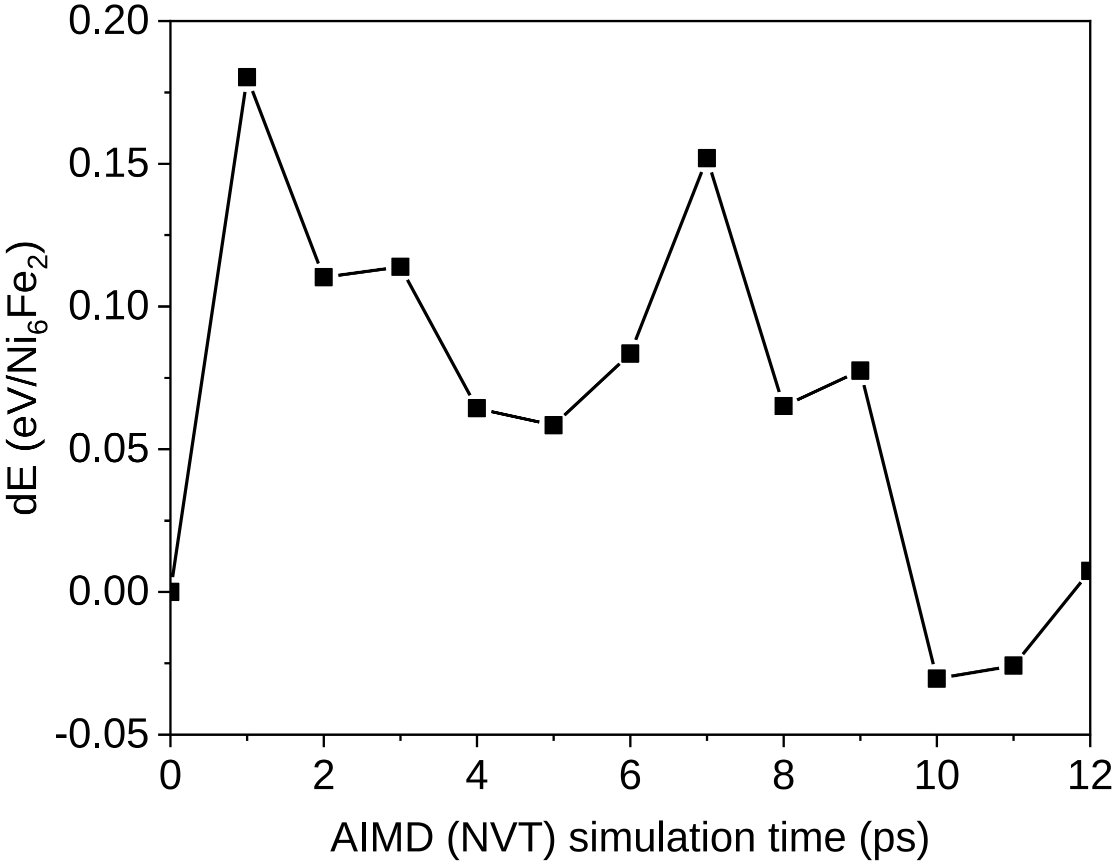

Supplementary Figure 28. Relative stability of α-NiFe LDH ($Ni_6Fe_2CO_3(OH)_{16}·4H_2O$) from AIMD simulations. The initial structure is constructed from the most stable configuration of MgAl hydrotalcite by changing Mg to Ni and Al to Fe.

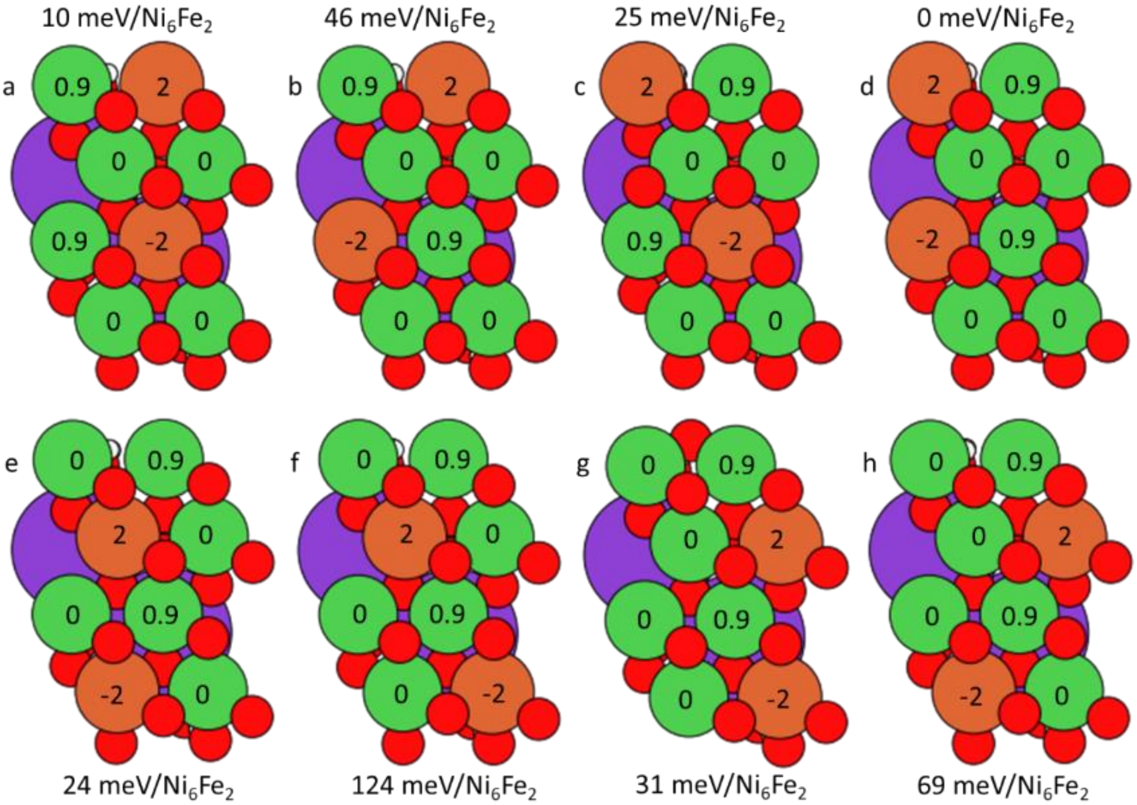

Supplementary Figure 29. Eight possible configurations of γ-NiFe LDH by replacing two non-nearest-neighbor Ni in γ-NiOOH with 2 Fe. The relative energies (per Ni_6Fe_2) and the magnetic moment of each cation are also given. Based on the characteristic magnetic moments, two Ni cations are in 3+ oxidation state with magnetic moment 0.9 μ_B, and four Ni cations are in 4+ oxidation state with magnetic moment 0 μ_B. Based on the charge balance, Fe cations are in 4+ oxidation state. Ni, Fe, K, O and H are represented by green, orange, purple, red and white balls, respectively.

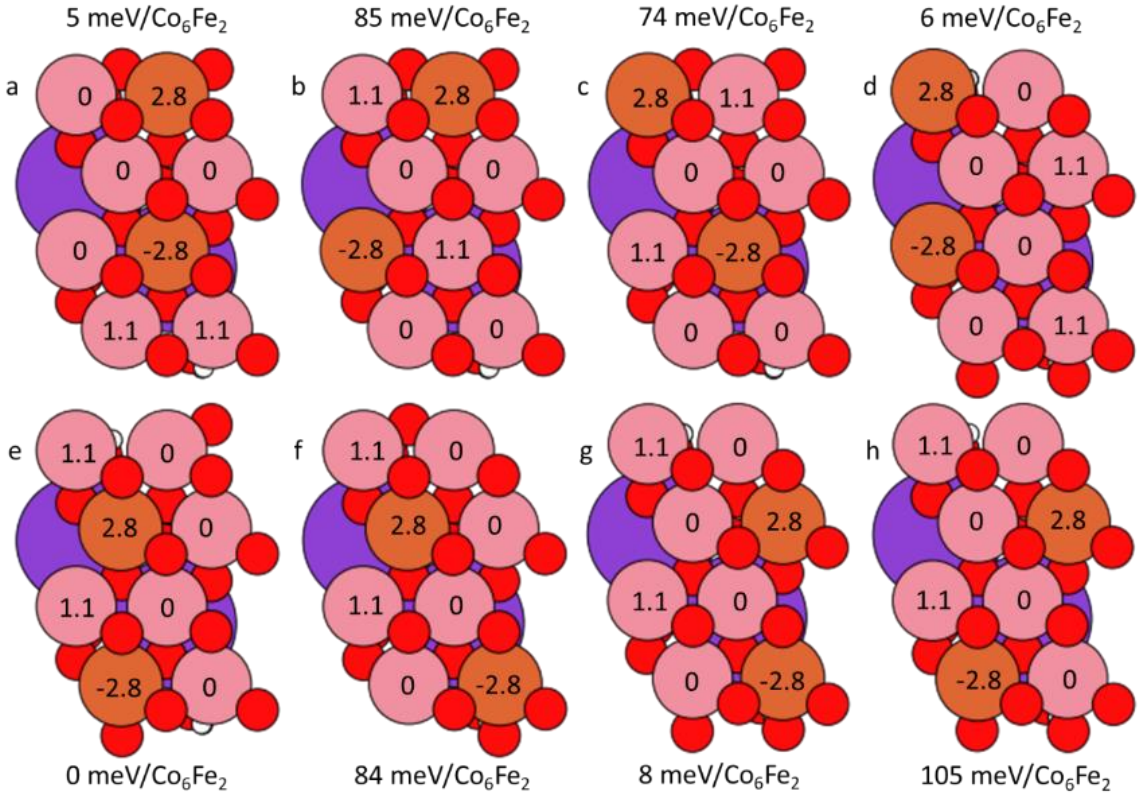

Supplementary Figure 30. Eight possible configurations of γ-CoFe LDH by replacing Ni in γ-NiFe LDH with Co. The relative energies (per Co_6Fe_2) and magnetic moment of the most stable magnetic configuration are also given for each geometric configuration. Based on the characteristic magnetic moments, four Co cations are in 3+ oxidation state with magnetic moment 0 μ_B, and two Co cations are in 4+ oxidation state with magnetic moment 1.1 μ_B. Based on the charge balance, Fe cations are in 5+ oxidation state. Co, Fe, K, O and H are represented by rose, orange, purple, red and white balls, respectively.

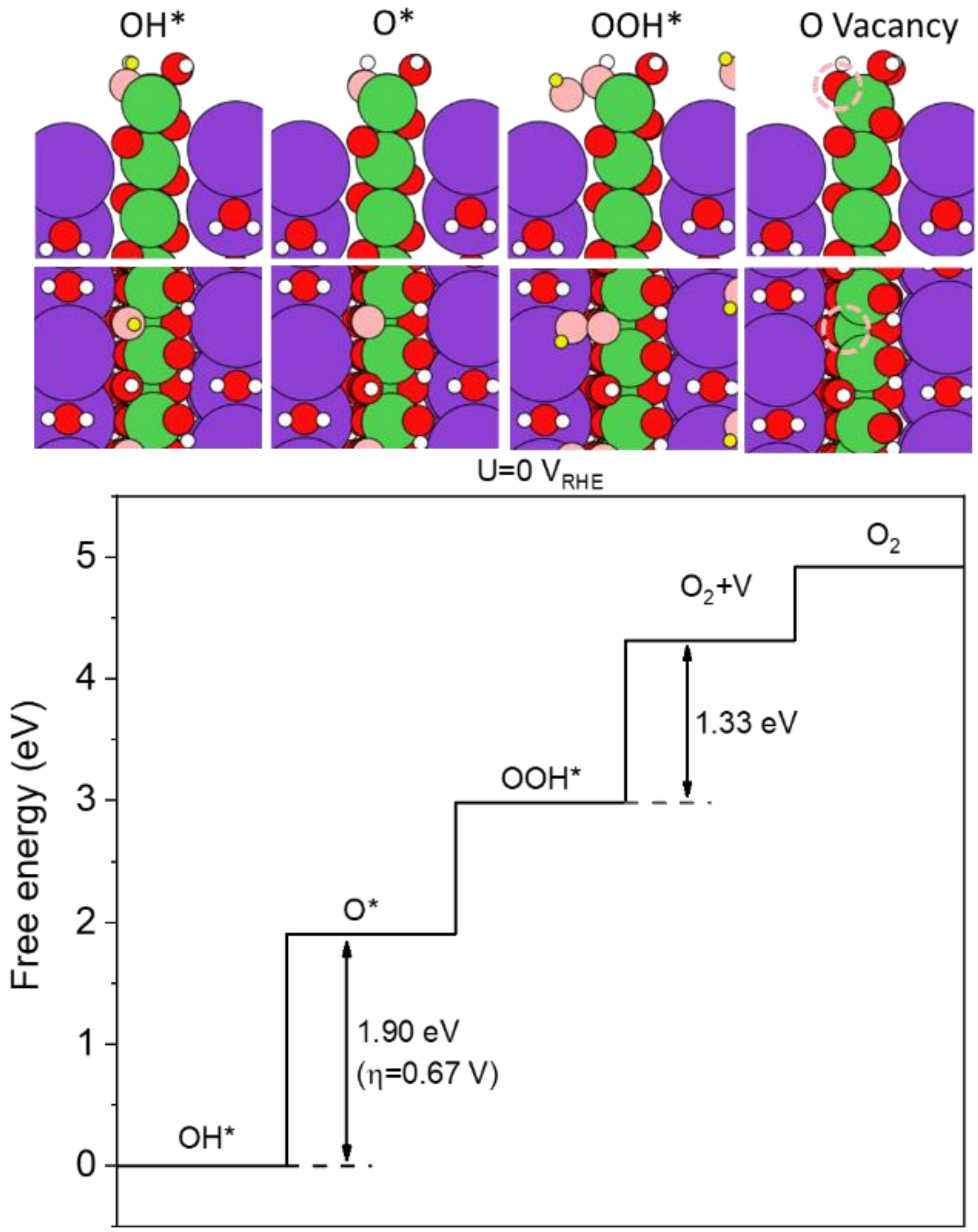

Supplementary Figure 31. OER Reaction free energies on bridge O site (Ni-O-Ni) of γ-NiOOH steady-state surface. The potential limiting steps and the overpotentials are given. The second highest free energy barrier is also given. Ni, K, O and H are represented by green, orange, purple, red and white balls, respectively. OER intermediates are differentiated by colors, yellow instead of white for H and rose instead of red for O, respectively. A dashed rose circle indicates the formation of a lattice vacancy on the surface.

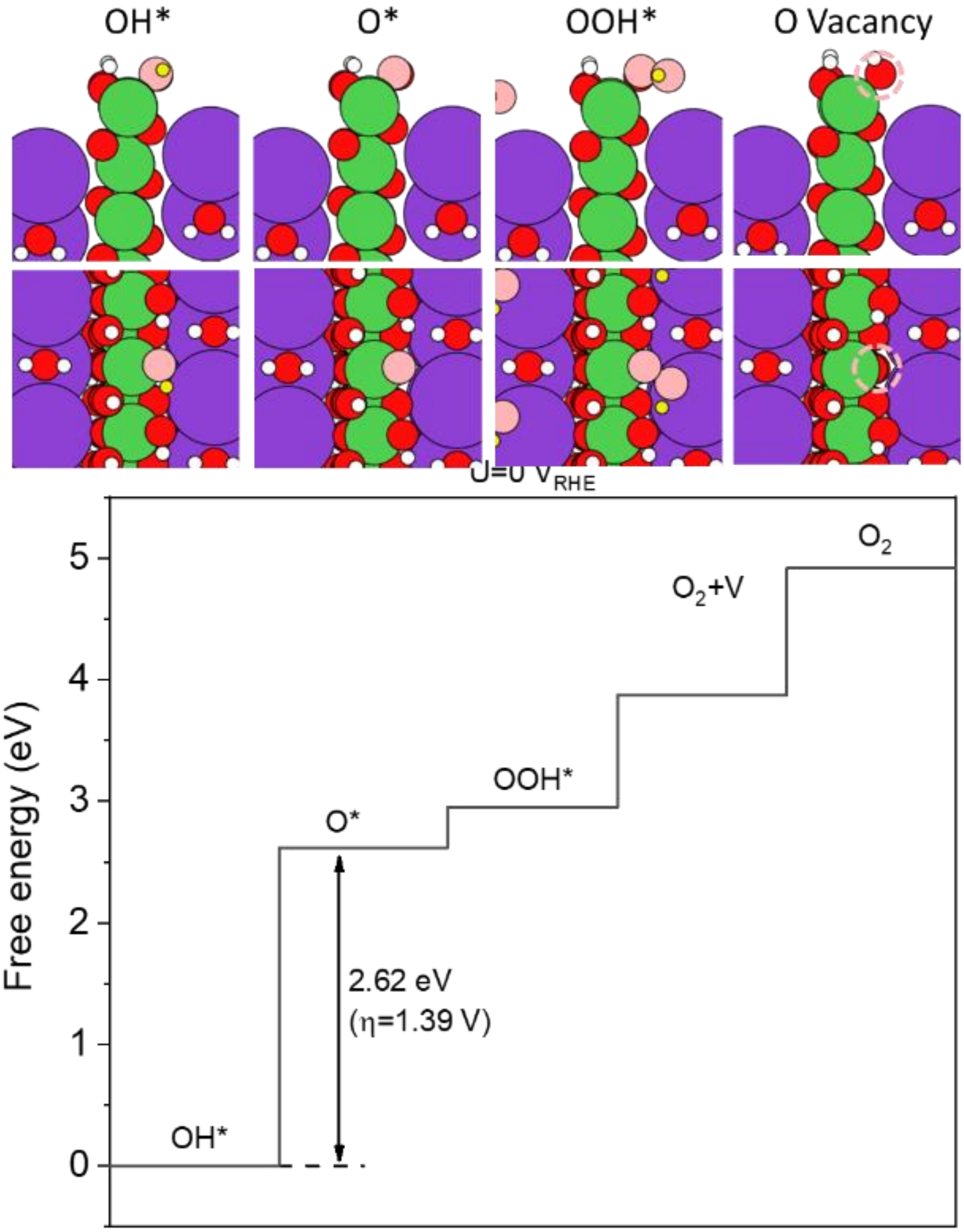

Supplementary Figure 32. OER Reaction free energies on Ni site of γ-NiOOH steady-state surface. The potential limiting steps and the overpotentials are given. Ni, K, O and H are represented by green, purple, red and white balls, respectively. OER intermediates are differentiated by colors, yellow instead of white for H and rose instead of red for O, respectively. A dashed rose circle indicates the formation of a lattice vacancy on the surface.

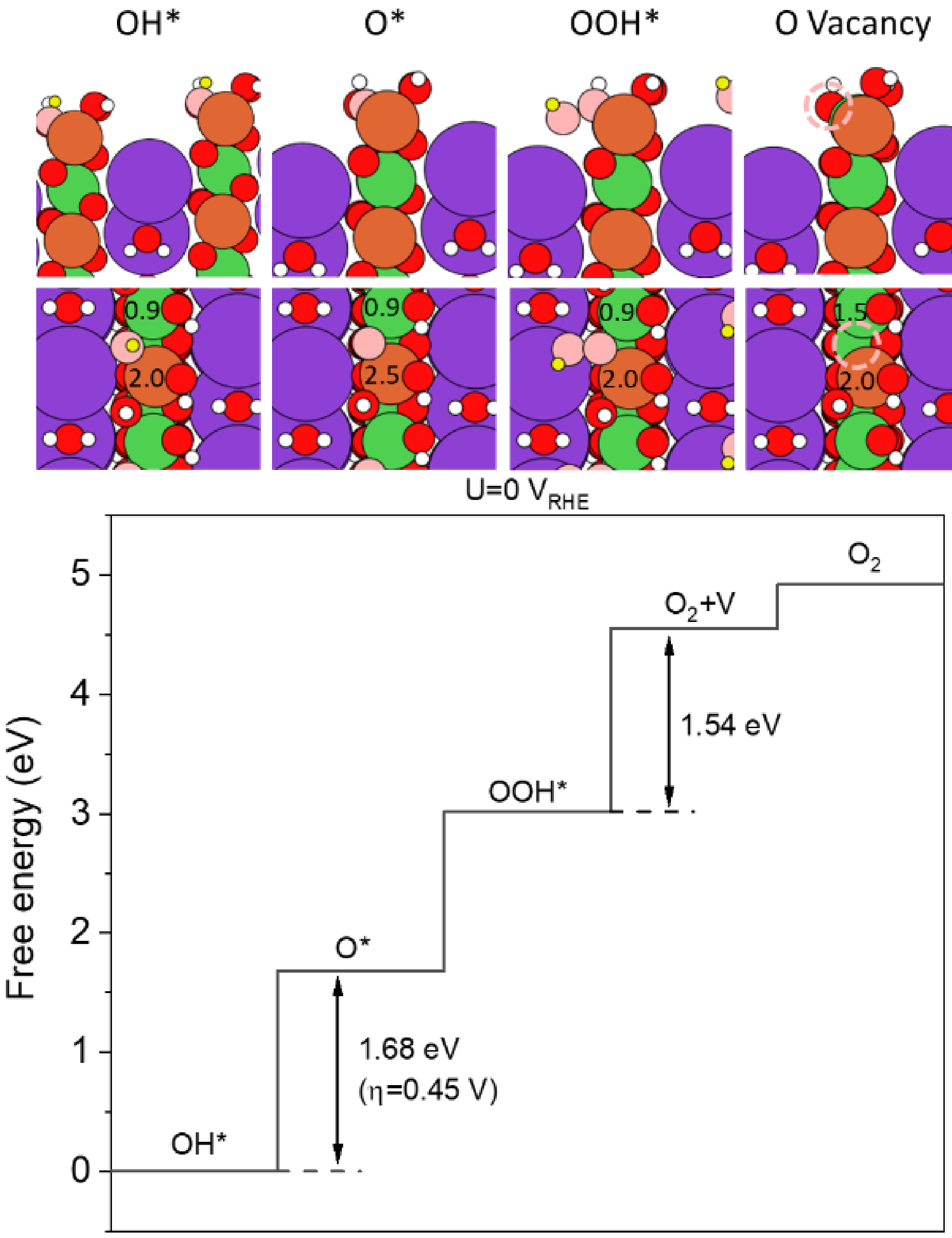

Supplementary Figure 33. OER Reaction free energies on bridge O site (Fe-O-Ni) of γ-NiFe LDH steady-state surface. The potential limiting steps and the overpotentials are given. The second highest free energy barrier is also given. Ni, Fe, K, O and H are represented by green, orange, purple, red and white balls, respectively. OER intermediates are differentiated by colors, yellow instead of white for H and rose instead of red for O, respectively. A dashed rose circle indicates the formation of a lattice vacancy on the surface.

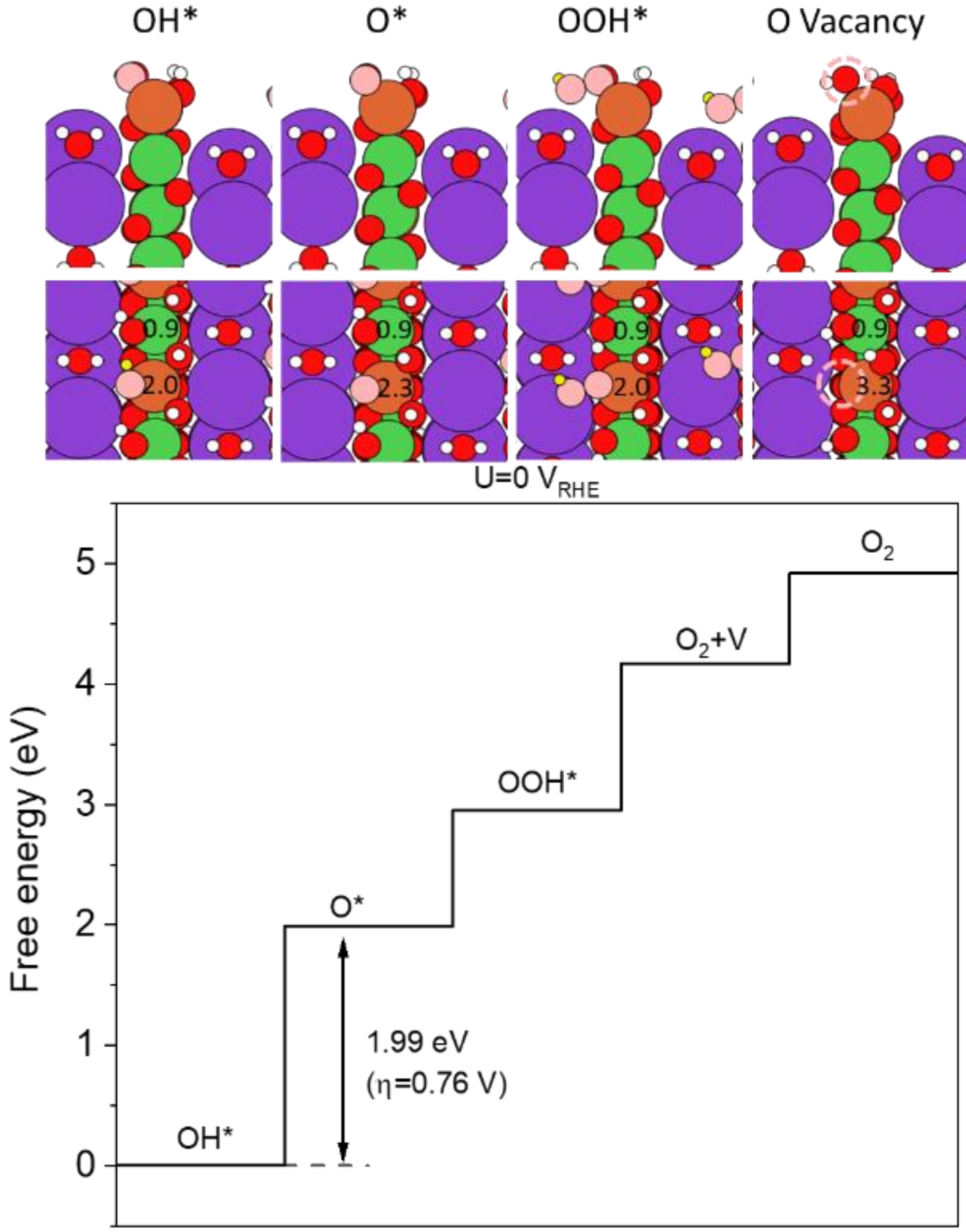

Supplementary Figure 34. OER Reaction free energies on Fe site of γ-NiFe LDH steady-state surface. The potential limiting steps and the overpotentials are given. Ni, Fe, K, O and H are represented by green, orange, purple, red and white balls, respectively. OER intermediates are differentiated by colors, yellow instead of white for H and rose instead of red for O, respectively. A dashed rose circle indicates the formation of a lattice vacancy on the surface.

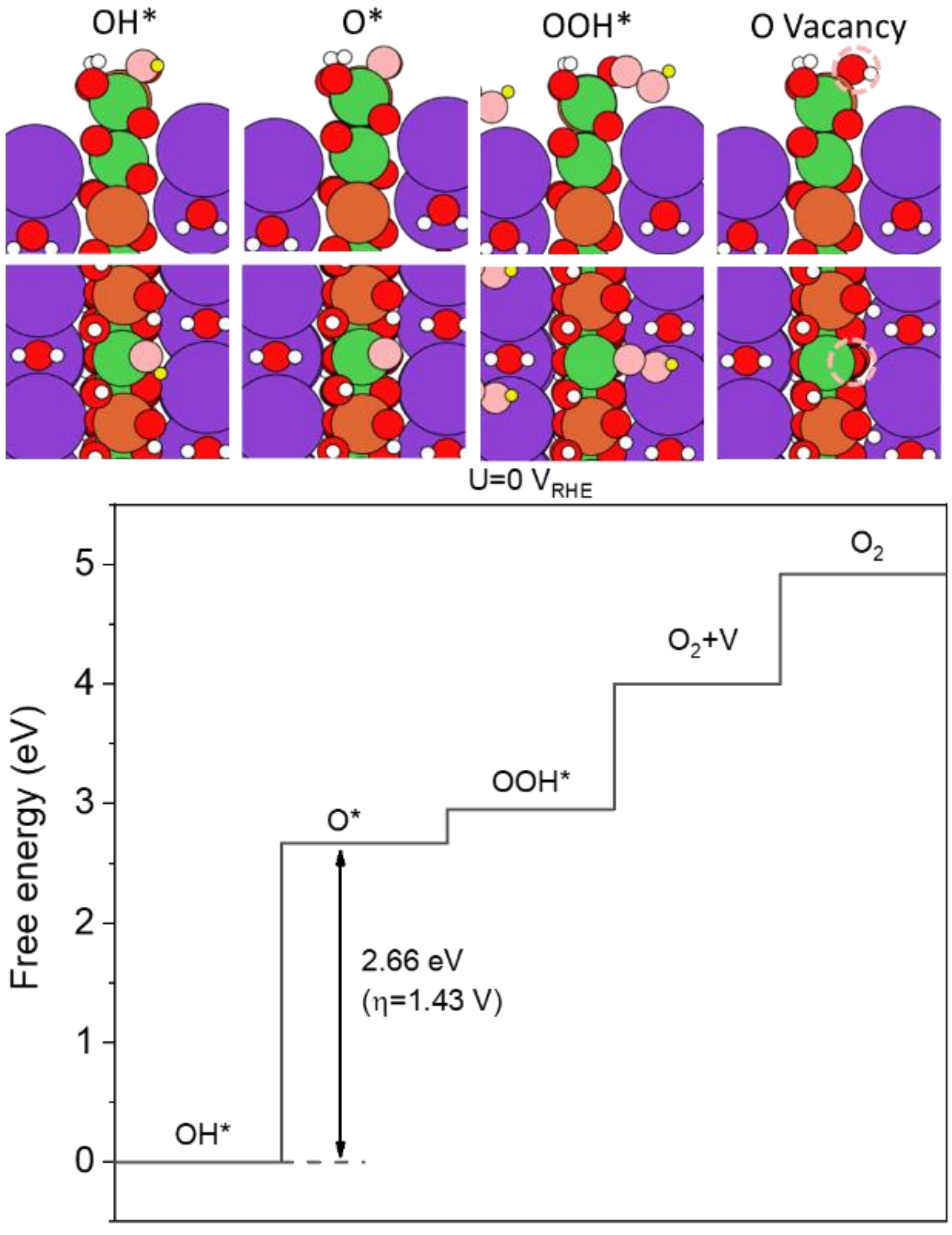

Supplementary Figure 35. OER Reaction free energies on Ni site of γ-NiFe LDH steady-state surface. The potential limiting steps and the overpotentials are given. Ni, Fe, K, O and H are represented by green, orange, purple, red and white balls, respectively. OER intermediates are highlighted by colors, yellow instead of white for H and rose instead of red for O, respectively. A dashed rose circle indicates the formation of a lattice vacancy on the surface.

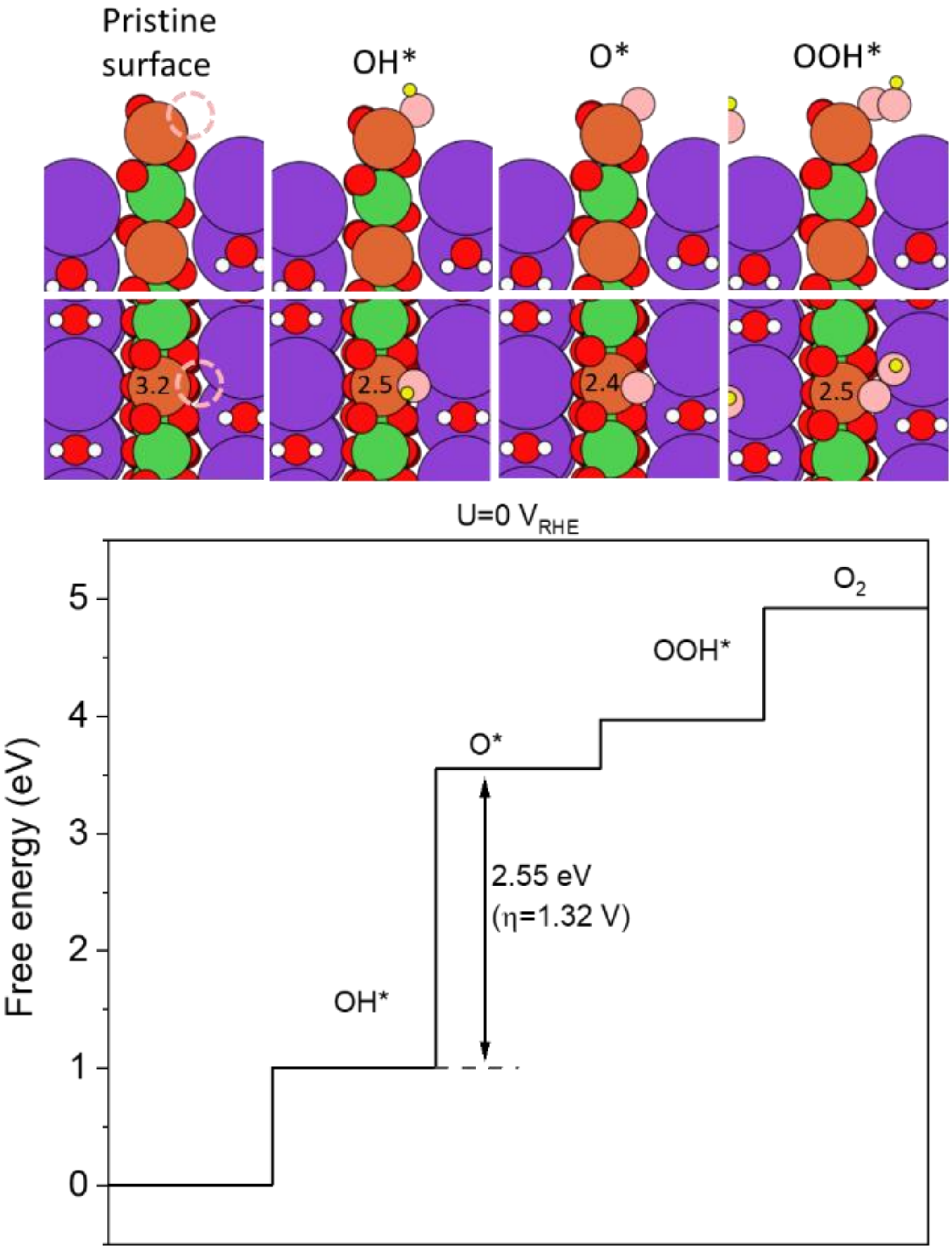

Supplementary Figure 36. OER Reaction free energies on the Fe site of γ-NiFe LDH pristine surface. The potential limiting steps and the overpotentials are given. Ni, Fe, K, O and H are represented by green, orange, purple, red and white balls, respectively. OER intermediates are differentiated by colors, yellow instead of white for H and rose instead of red for O, respectively. A dashed rose circle indicates the site for the adsorption of intermediates.

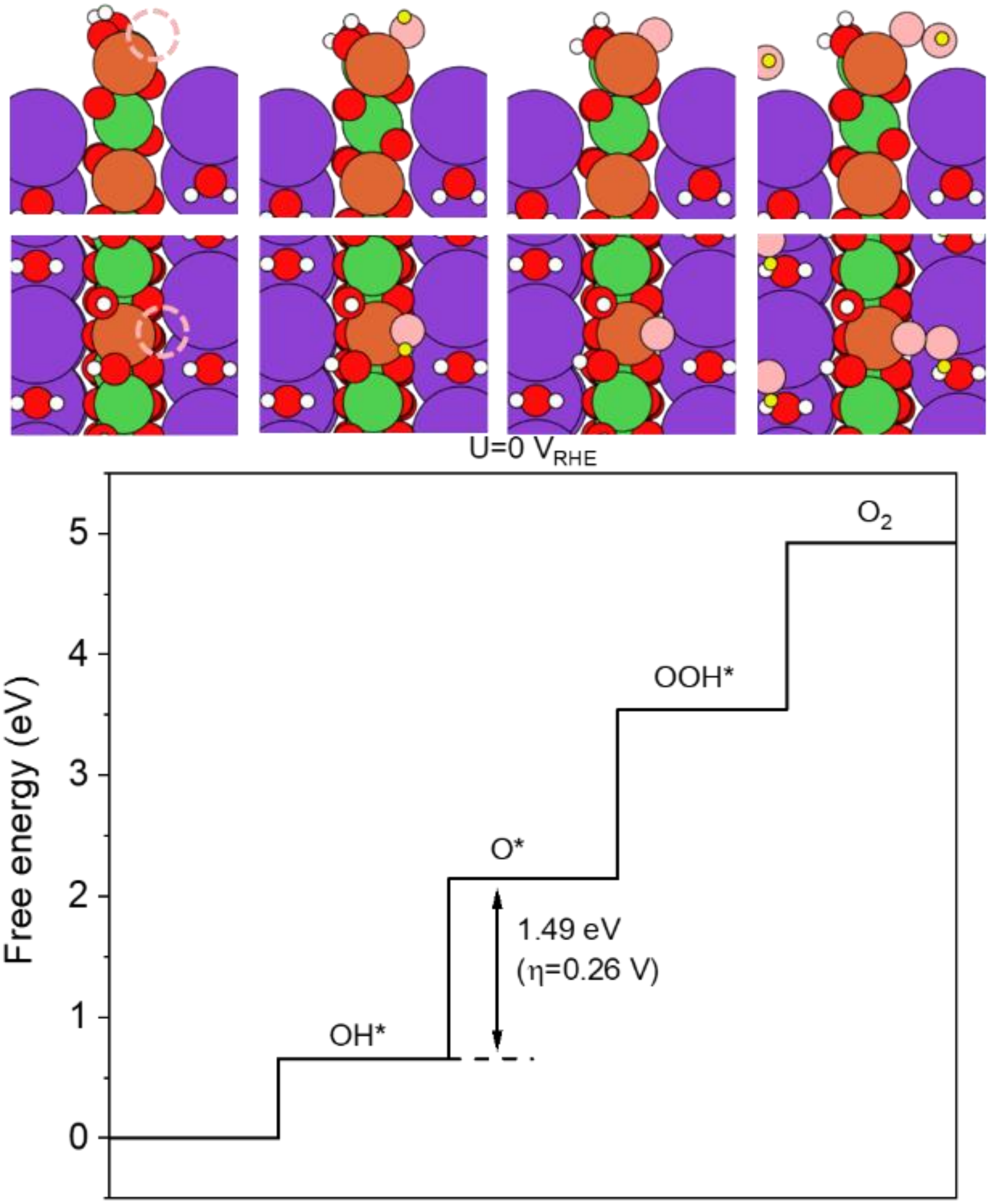

Supplementary Figure 37. OER Reaction free energies on the Fe site of γ-NiFe LDH steady-state surface at low potential (< 1 V). The potential limiting steps and the overpotentials are given. Ni, Fe, K, O and H are represented by green, orange, purple, red and white balls, respectively. OER intermediates are differentiated by colors, yellow instead of white for H and rose instead of red for O, respectively. A dashed rose circle indicates the site for the adsorption of intermediates.

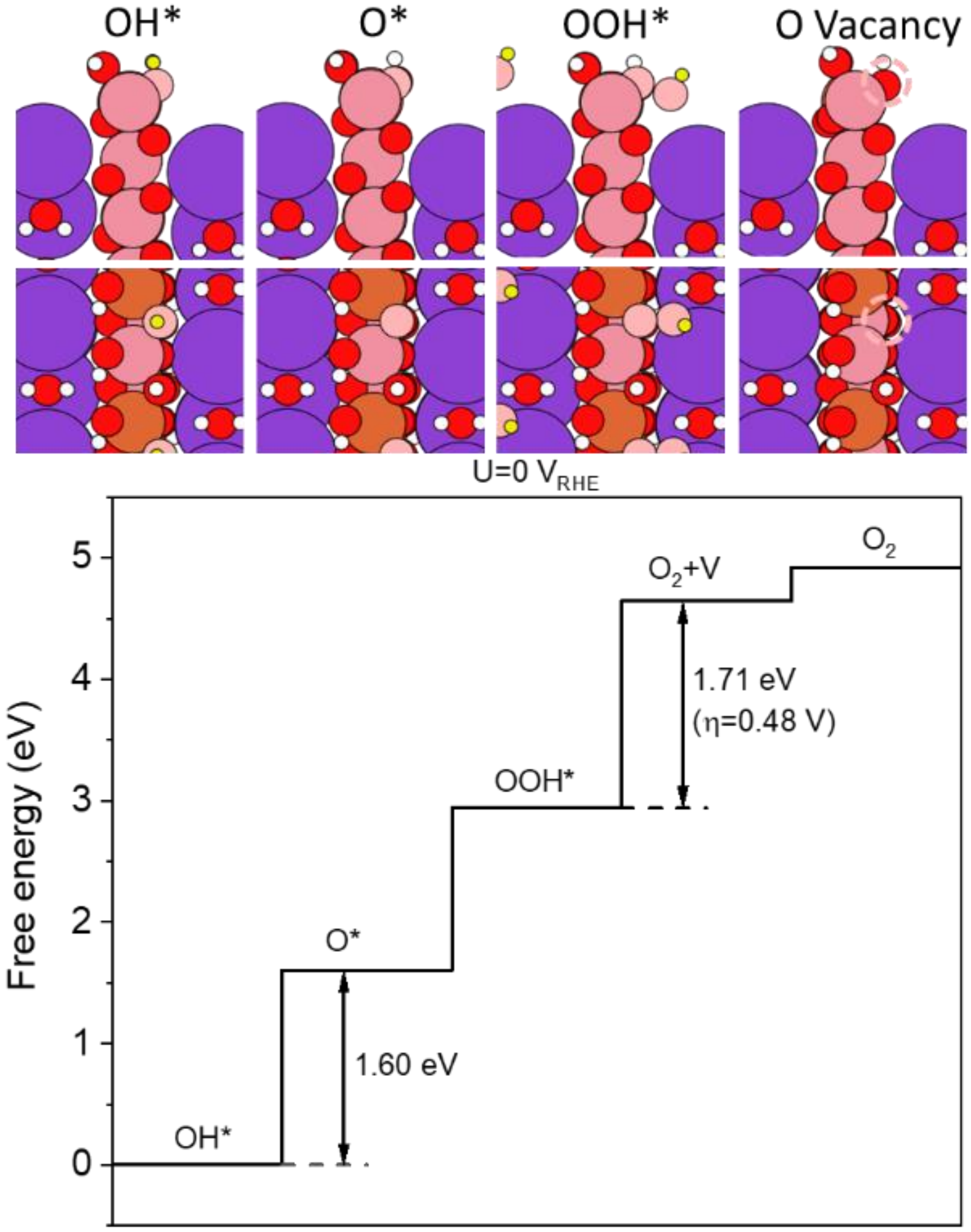

Supplementary Figure 38. OER Reaction free energies on bridge O site (Fe-O-Co) of γ-CoFe LDH steady-state surface. The potential limiting steps and the overpotentials are given. The second highest free energy barrier is also given. Co, Fe, K, O and H are represented by rose, orange, purple, red and white balls, respectively. OER intermediates are differentiated by colors, yellow instead of white for H and rose instead of red for O, respectively. A dashed rose circle indicates the formation of a lattice vacancy on the surface.

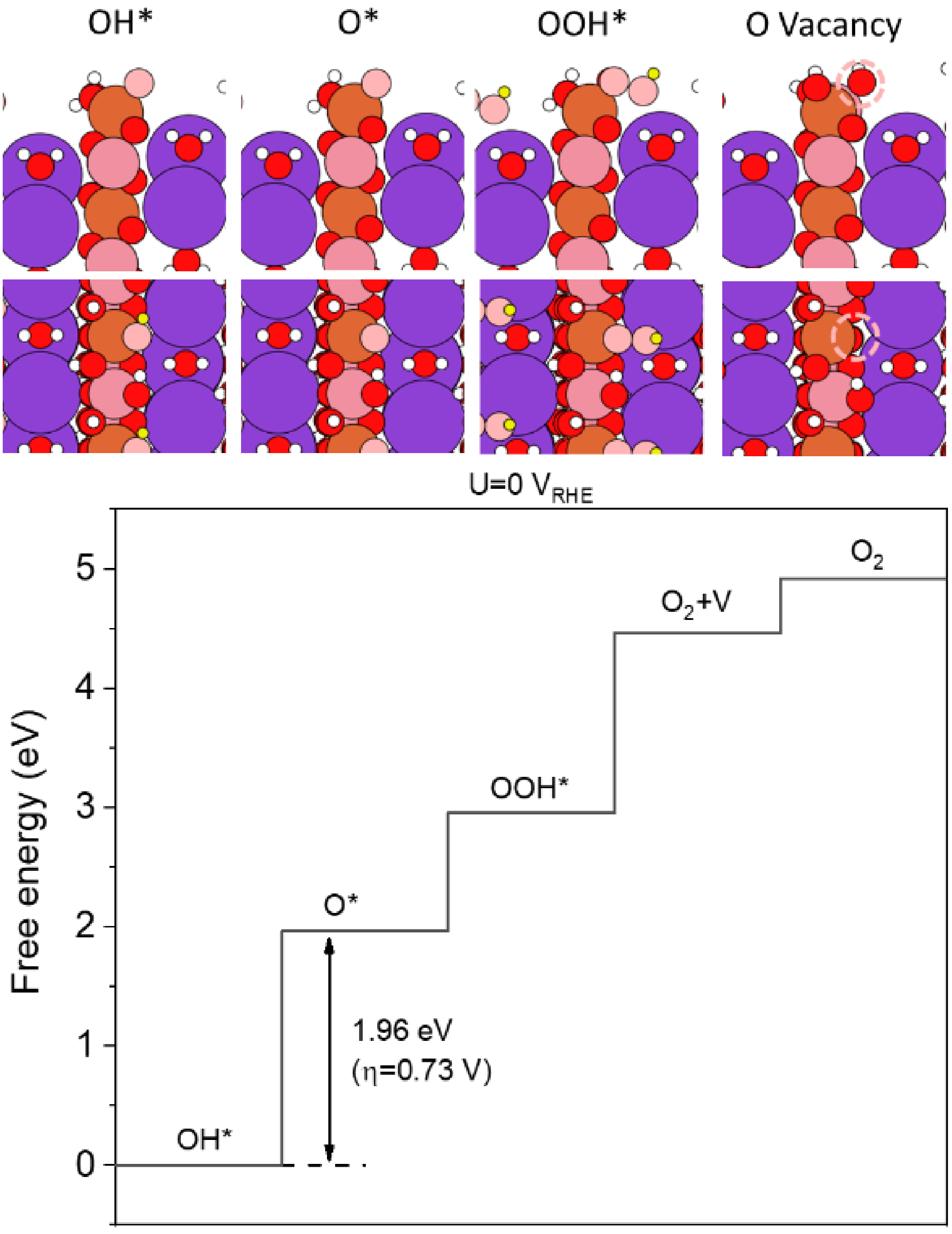

Supplementary Figure 39. OER Reaction free energies on Fe site of γ-CoFe LDH steady-state surface. The potential limiting steps and the overpotentials are given. Co, Fe, K, O and H are represented by rose, orange, purple, red and white balls, respectively. OER intermediates are differentiated by colors, yellow instead of white for H and rose instead of red for O, respectively. A dashed rose circle indicates the formation of a lattice vacancy on the surface.

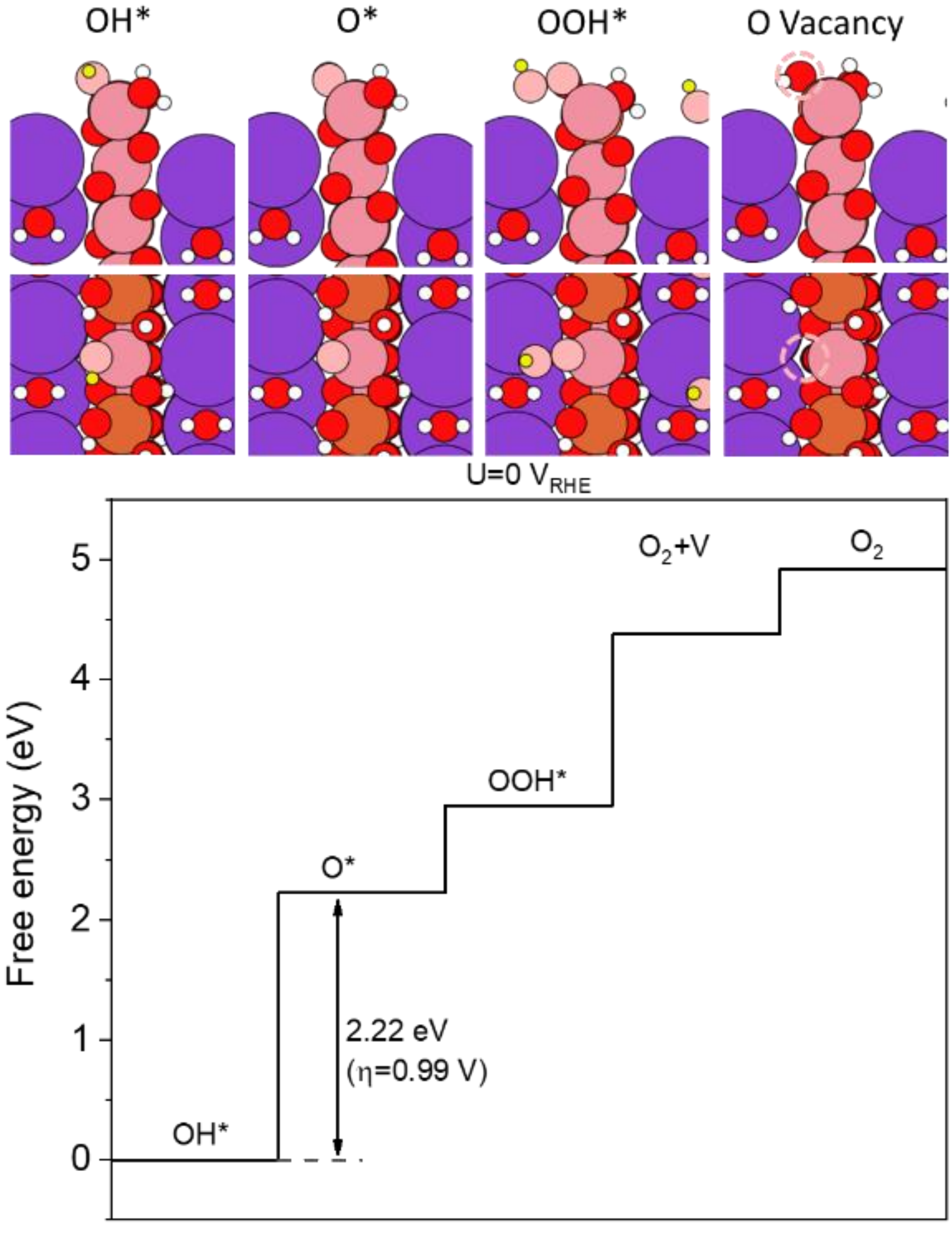

Supplementary Figure 40. OER Reaction free energies on Co site of γ-CoFe LDH steady-state surface. The potential limiting steps and the overpotentials are given. Co, Fe, K, O and H are represented by rose, orange, purple, red and white balls, respectively. OER intermediates are differentiated by colors, yellow instead of white for H and rose instead of red for O, respectively. A dashed rose circle indicates the formation of a lattice vacancy on the surface.

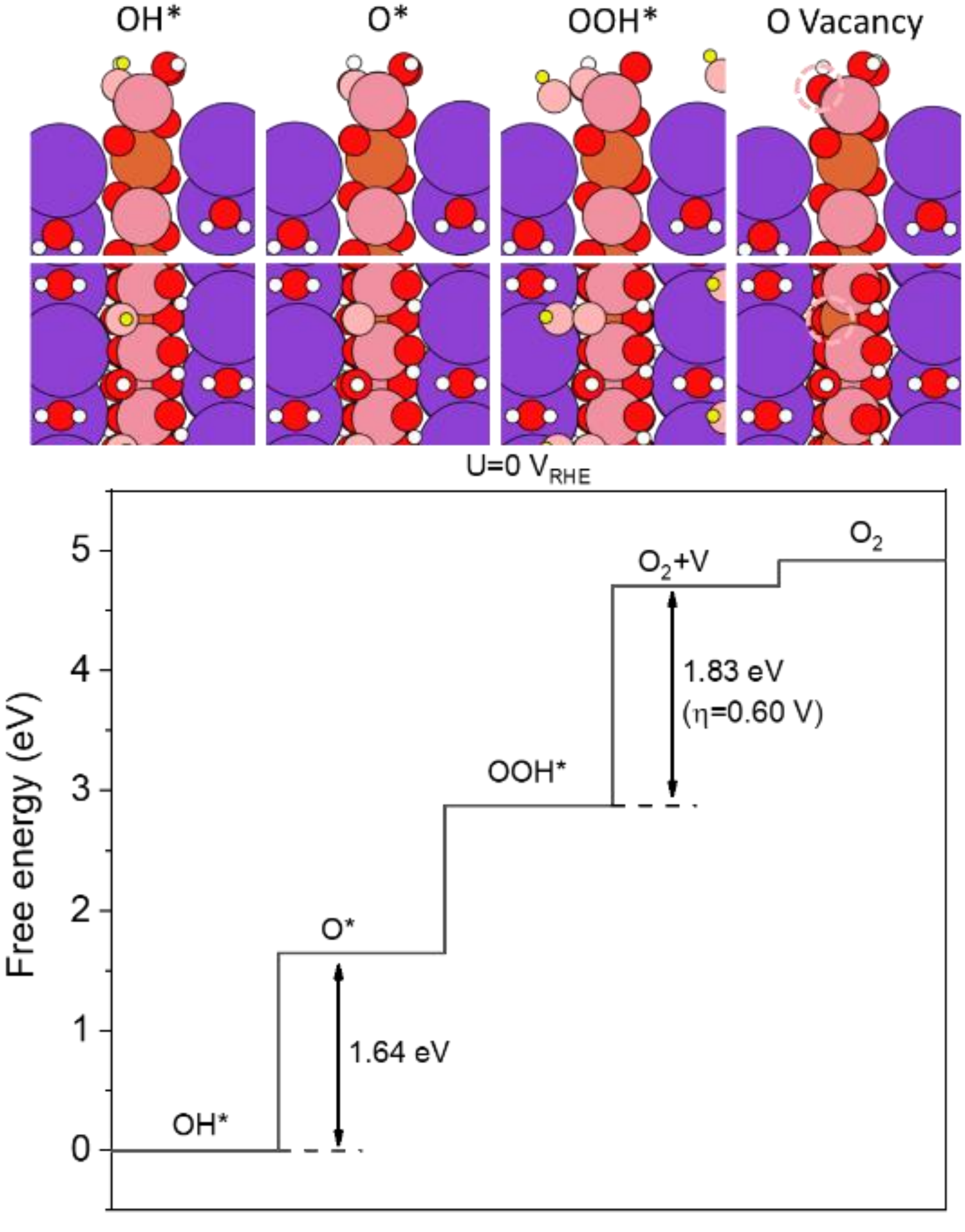

Supplementary Figure 41. OER Reaction free energies on bridge O site (Co-O-Co) of γ-CoFe LDH steady-state surface. The potential limiting steps and the overpotentials are given. The second highest free energy barrier is also given. Co, Fe, K, O and H are represented by rose, orange, purple, red and white balls, respectively. OER intermediates are differentiated by colors, yellow instead of white for H and rose instead of red for O, respectively. A dashed rose circle indicates the formation of a lattice vacancy on the surface.

Supplementary Tables

Supplementary Table 1. EXAFS Fit Results for the Ni K-edge in NiFe LDH.

	CN	r, Å	$\sigma^2 \cdot 10^3$	ΔE
Ni-O (As prepared)	5.8(3)	2.06(1)	6.1(9)	1.5(9)
Ni-M (As prepared)	5.6(8)	3.08(2)	8.6(8)	-1.1(2)
Ni-O (1.0 V_{RHE})	5.6(3)	2.05(1)	7.6(14)	3.4(8)
Ni-M (1.0 V_{RHE})	5.9(9)	3.09(1)	9.6(16)	4.5(7)
Ni-O (1.55 V_{RHE})	4.5(4)	2.05(1)	10.2(24)	4.3(9)
Ni-M (1.55 V_{RHE})	4.8(9)	3.11(1)	6.7±(7)	5.6(9)
Ni-O (1.7 V_{RHE})	4.2(5)	2.04(2)	12.1(26)	5.1(9)
Ni-M (1.7 V_{RHE})	4.3(7)	3.12(2)	4.7(9)	6.5(15)
Ni-O (1.0 V_{RHE} after OER)	5.4(3)	2.06(1)	6.9(9)	1.5(3)
Ni-M (1.0V_{RHE} after OER)	5.8(8)	3.09(1)	10.0±(9)	3.9(9)

Supplementary Table 2. EXAFS Fit Results for the Fe K-edge in NiFe LDH.

	CN	r, Å	$\sigma^2 \cdot 10^3$	ΔE
Fe-O (As prepared)	4.8(4)	2.01(1)	6.2(4)	4.7(4)
Fe-M (As prepared)	4.0(7)	3.13(2)	5.1(7)	6.1(5)
Fe-O (1.0 V_{RHE})	4.6(5)	1.99(2)	6.8(4)	2.1(2)
Fe-M (1.0 V_{RHE})	3.6(3)	3.12(3)	5.7(6)	6.9(8)
Fe-O (1.55 V_{RHE})	4.4(4)	1.98(2)	7.9(5)	2.5(2)
Fe-M (1.55 V_{RHE})	3.3(4)	3.14(3)	6.0(7)	8.7(9)
Fe-O (1.7 V_{RHE})	4.2(6)	1.98(2)	9.8(7)	2.5(2)
Fe-M (1.7 V_{RHE})	2.9(3)	3.17(3)	5.3(5)	11.9(10)
Fe-O (1.0 V_{RHE} back swing)	4.8(4)	1.99(1)	7.2(4)	2.2(1)
Fe-M (1.0 V_{RHE} back swing)	3.7(3)	3.12(3)	5.1(4)	6.9(6)

Supplementary Table 3. EXAFS fit results for the Co K-edge in CoFe LDH.

	CN	r, Å	$\sigma^2 \cdot 10^3$	ΔE
Co-O (As prepared)	4.4(5)	2.06(3)	14.9(7)	3.9(6)
Co-M (As prepared)	2.4(3)	3.12(5)	9.1(8)	4.5(7)
Co-O (1.0 V_{RHE})	3.2(6)	1.93(4)	3.9(6)	7.4(2)
Co-M (1.0 V_{RHE})	2.6(3)	2.86(4)	10.1(4)	3.1(2)
Co-O (1.3 V_{RHE})	4.5(5)	1.91(2)	4.0(4)	6.4(6)
Co-M (1.3 V_{RHE})	3.5(9)	2.84(2)	9.2(5)	2.4(2)
Co-O (1.6 V_{RHE})	4.7(4)	1.89(1)	5.1(6)	3.6(3)
Co-M (1.6 V_{RHE})	3.7(8)	2.84(1)	8.2(5)	2.4(2)
Co-O (1.75 V_{RHE})	4.8(4)	1.89(2)	5.1(5)	3.8(8)
Co-M (1.75 V_{RHE})	3.6(7)	2.84(1)	8.2(7)	2.2(3)

Supplementary Table 4. EXAFS fit results for the Fe K-edge in CoFe LDH.

	CN	r, Å	$\sigma^2 \cdot 10^3$	ΔE
Fe-O (As prepared)	4.0(6)	1.99(2)	5.1(4)	2.9(1)
Fe-M (As prepared)	3.5(2)	3.13(6)	7.9(3)	4.3(3)
Fe-Co (As prepared)	2.4(3)	3.7(4)	11.2(8)	5.6(4)
Fe-O (1.0 V_{RHE})	3.0(2)	1.96(1)	6.3(5)	3.8(8)
Fe-M (1.0 V_{RHE})	3.8(4)	2.93(4)	8.1(3)	-1.7(4)
Fe-Co (1.0 V_{RHE})	2.0(2)	3.6(4)	12.7(10)	5.1(5)
Fe-O (1.3 V_{RHE})	3.2(2)	1.92(2)	5.6(3)	1.4(2)
Fe-M (1.3 V_{RHE})	4.2(3)	2.76	7.9(3)	-2.3(2)
Fe-Co (1.3 V_{RHE})	1.7(2)	3.5(3)	12.6(11)	5.5(4)
Fe-O (1.6 V_{RHE})	3.1(2)	1.92(1)	7.1(5)	3.0(7)
Fe-M (1.6 V_{RHE})	4.2(3)	2.77(2)	6.9(5)	-2.4(2)
Fe-Co (1.6 V_{RHE})	1.2(1)	3.4(4)	10.2(7)	5.5(5)
Fe-O (1.75 V_{RHE})	3.2(2)	1.91(1)	6.8(3)	2.2(5)
Fe-M (1.75 V_{RHE})	4.4(2)	2.80(2)	6.8(6)	-2.3(3)
Fe-Co (1.75 V_{RHE})	1.7(2)	3.5(4)	10.5(8)	5.3(4)

Supplementary Table 5. Magnetic moment (and the number of atoms with that magnetic moment in the unit cell) and structural parameters and free energy of formation (ΔG_{form}) of Mg, Al, Fe, Co and Ni (hydroxyl)oxides and layered double hydroxides. ΔG_{form} of LDH, which is normalized by the total number of metal atoms in the layer, is calculated with respective to the anhydrous (hydroxyl)oxides in the table, water, and ions. For hydrotalcite and α-MFe LDHs, the reference ions is CO_3^{2-} that is in equilibrium with CO_2 in atmosphere (400 ppm). For γ-NiOOH and γ-MFe LDHs, the reference ion is K^+ which has the standard reduction potentials 2.4 V lower than K_2O. See computational method for more details.

	Magnetic moment (μ_B)			Lattice constant (Å)			ΔG_{form}
	Fe	Co	Ni	a	b	c	(eV/M)
$Mg(OH)_2$				3.18		4.77	
β-$Co(OH)_2$		2.74		3.23		4.66	
β-$Ni(OH)_2$			1.76	3.16		4.62	
γ-AlOOH				2.90	12.19	3.74	
α-FeOOH	4.06			4.65	10.08	3.04	
β-CoOOH		0.00		2.87		13.21	
β-NiOOH			1.05	2.95		4.62	
CoO_2		1.22		2.84		4.45	
NiO_2			0.00	2.80		4.51	
Hydrotalcite (MgAl LDH)				3.08		23.11	-0.08
α-NiFe LDH	4.21		1.76	3.10		23.01	-0.03
α-CoFe LDH	4.20	2.74		3.15		23.02	-0.01
γ-NiOOH			0.90 (2), 0.03 (6)	2.81		7.18	-0.04
γ-NiFe LDH	2.00 (2)		0.91 (2), 0.06 (4)	2.83		7.18	-0.03
γ-CoFe LDH	2.80 (2)	0.01 (4), 1.12 (2)		2.84		7.17	-0.08

Supplementary Table 6. Magnetic moment (and the number of atoms with that magnetic moment in the unit cell), structural parameters and free energy of formation (ΔG_{form}) of β-MFe LDHs. ΔG_{form} is calculated with respective to the anhydrous (hydroxyl)oxides in the Supplementary Table 5.

	Magnetic moment (μ_B)			Lattice constant (Å)		ΔG_{form}
	Fe	Co	Ni	a	c	(eV/M)
β-NiFeOOH	3.53, 3.40		1.70 (2), 1.05 (4)	2.89	4.61	0.03
β-NiFeOOH$_{0.25}$	2.10 (2)		0.02 (4), 0.96 (2)	2.84	4.70	0.10
β-NiFeO$_2$	2.10 (2)		0.03 (6)	2.82	4.76	
β-CoFeOOH	4.16 (2)	0.01 (6)		2.93	4.45	0.11
β-CoFeOOH$_{0.25}$	2.03, 2.79	0.02 (3), 1.14 (3)		2.85	4.56	0.06
β-CoFeO$_2$	2.87, 2.74	0.04 (2), 1.15 (4)		2.84	4.71	

Supplementary Table 7. Reaction (free) energy on the γ-NiOOH surface.

	dE (eV)	dG(eV)
Bridge OH		
OH*→O*	1.96	1.90
OH*→OOH*	3.06	2.98
OH*→V+O_2	4.90	4.31
OH*→OH*+O_2		4.92
Atop OH		
OH*→O*	2.68	2.62
OH*→OOH*	2.34	2.95
OH*→V+O_2	4.46	3.87
OH*→OH*+O_2		4.92

Supplementary Table 8. Reaction (free) energy on the γ-NiFe LDH surface.

	dE (eV)	dG (eV)
Bridge OH at Fe-Ni reaction center		
OH*→O*	1.74	1.68
OH*→OOH*	3.09	3.02
OH*→V+O_2	5.14	4.55
OH*→OH*+O_2		4.92
Atop OH on Fe site		
OH*→O*	2.05	1.99
OH*→OOH*	2.78	2.95
OH*→V+O_2	4.75	4.17
OH*→OH*+O_2		4.92
Atop OH on Ni site		
OH*→O*	2.78	2.72
OH*→OOH*	2.45	2.95
OH*→V+O_2	4.64	4.05
OH*→OH*+O_2		4.92
Fe site on pristine surface		
OH*	0.91	1.00
O*	3.53	3.55
OOH*	3.96	3.97
O_2		4.92
Fe site on hydrogenated surface at potential < 1V		
OH*	0.68	0.77
O*	2.23	2.25
OOH*	3.64	3.65
O_2		4.92

Supplementary Table 9. OER overpotentials for (01-10) surfaces of γ-NiFe LDH configurations that are within 50 meV in comparison with the most stable configuration (see Supplementary Figure 29. As these configurations, albeit with different arrangements of Ni and Fe atoms to reflect the different bulk configurations, could co-exist because of their closeness in energy, the comparison indicates that both the same mechanism (MvK) and the same active site (O-bridged NiFe center) will be favorable for all of them. Therefore a possible variation of the configuration will not alter the conclusion.

Configuration	Relative stability (meV)	Conventional mechanism (V)	MvK mechanism (V)	
		Fe site	Fe site	O bridged Ni-Fe center
1	10	1.08	0.67	0.44
2	46	1.05	0.68	0.46
3	25	0.93	0.68	0.47
4	0	1.32	0.76	0.45
5	24	1.07	0.78	0.39
7	31	0.98	0.66	0.48

Supplementary Table 10. Reaction (free) energy on the γ-CoFe LDH surface.

	dE (eV)	dG (eV)
Bridge OH at Fe-Co reaction center		
OH*→O*	1.64	1.58
OH*→OOH*	3.01	2.94
OH*→V+O_2	5.24	4.65
OH*→OH*+O_2		4.92
Atop OH on Fe site		
OH*→O*	2.02	1.96
OH*→OOH*	2.86	2.95
OH*→V+O_2	5.04	4.46
OH*→OH*+O_2		4.92
Atop OH on Co site		
OH*→O*	2.28	2.22
OH*→OOH*	3.34	2.95
OH*→V+O_2	4.96	4.37
OH*→OH*+O_2		4.92
Bridge OH at Co-Co reaction center		
OH*→O*	1.70	1.64
OH*→OOH*	2.95	2.87
OH*→V+O_2	5.29	4.70
OH*→OH*+O_2		4.92

Supplementary Table 11. Thermodynamic correction used in the free energy calculations. Zero-point energies are calculated with experimental vibrational data in Ref.[9,10] , the integrated heat capacity ($\delta H^{0\rightarrow 298K}$) and entropy at 298.15 K are obtained from Ref.[11]. For water, the entropy is calculated at 0.035 bar through $S = S_0 + k_B T ln(p/p^0)$ to derive the chemical potential of liquid water, because at this pressure gas-phase water is in equilibrium with liquid water at 298.15 K. The solvation energies ($E_{solvation}$) are evaluated from AIMD simulation by filling the vacuum with liquid water with a thickness that is equivalent to 5 water bilayers. It worth noting that, in comparison with our previous work,[12,13] the solvation energy of surface OH is about 0.3 eV smaller, because of high OH density in the current case which leads to a very limited amount of hydrogen bonds formed with interface water.

	ZPE (eV)	δH@298K (eV)	TS@298K (eV)	$E_{solvation}$(eV)
H_2O	0.56	0.10	0.68	
H_2	0.27	0.09	0.41	
OOH*	0.47	0.05	0.08	-0.4
O*	0.07	0.03	0.05	0
OH*	0.39	0.03	0.03	-0.3

Supplementary Methods

Synthesis of NiFe LDH

The synthesis of NiFe LDH (Ni:Fe=3.55:1) is described in ref.[14] with the difference that for the work presented here the carbon supporting step was omitted. Briefly: a solution of 16 ml DMF and 34 ml H_2O with Ni(II)$(OAc)_2$ (0.320 mmol) and Fe(III)$(NO_3)_3$ (0.107 mmol) was prepared in an autoclave glass liner and handed in a steel autoclave (Roth, 100 mL/100 bar Model I). The solution was heated during the hydrothermal treatment for 16 hours at 130 °C, followed by 2 hours at 170 °C. The reaction solution was let cool down naturally. Afterwards, the washing protocol was applied. First, the reaction solution was centrifuged at 8500 rpm for 15 min. The solution was poured away and the nanoparticles were redispersed in 40 ml of a water/ethanol mixture (3:1). The solution was centrifuged again and poured away. The precipitate was twice redispersed in 30 ml H_2O, centrifuged and poured away. Afterwards the particles were freeze-dried overnight. A yellowish powder was obtained.

Synthesis of CoFe LDH

For the synthesis of CoFe LDH (Co:Fe=3.33:1), Cobalt-II-acetate tetra hydrate (79.71 mg,) was dissolved in 43.8 mL of ultra-pure water. 320 µL of a 0.6 M Iron-III-nitrate was added to the solution. The solution was purged with Nitrogen for half an hour. Under continues stirring 5 mL of a 1 M potassium carbonate solution were add together to the $CoCl_2/Fe(NO_3)_3$ solution. A brown precipitation was formed. After an hour of aging the blackish brown solid product was recovered by centrifuge (8500 rpm, 15 min). The precipitation was washed two times with a water-ethanol mix (3:1) and two times with water. The product was freeze-dried overnight. The dry product was dispersed in 34 mL ultra-pure water and 16 mL N,N-Dimethylformamide (DMF) in an autoclave tube. The suspension was purged with Nitrogen for half an hour. The autoclave tube was inserted in the autoclave. The autoclave was purged with Nitrogen for an hour to drive out all Oxygen. For half an hour, the autoclave was heated at 80 °C with stirring to avoid boiling retardation. After that the stirring was stopped and the suspension was heated of 16 hours at 80 °C. Then the temperature was raised to 100 °C for two hours. At last the reaction was cool down without heating for two hours. The brown solid product was recovered by centrifuge (8500 rpm, 15 min). The precipitation was washed two times with a water-ethanol mix (3:1) and two times with water. The product was freeze-dried overnight.

Synthesis of β-$Ni(OH)_2$

$Ni(OH)_2$ was synthesized using a two-step synthesis consisting of a precipitation step and a hydrothermal treatment afterwards. First, 0.603 mmol of Ni(II)$(OAc)_2$ were dissolved in 50 ml H_2O in an autoclave-glass. To precipitate the $Ni(OH)_2$ nanoparticles, 1 ml 1 M KOH solution was added. The reaction solution was stirred for 10 min and then handed in a glass lined steel autoclave. The solution was heated during the hydrothermal treatment for 16 hours at 130 °C, followed by 2 hours at 170 °C. The reaction solution was let cool down naturally. Afterwards, the washing protocol was applied. First, the reaction solution was centrifuged at 8500 rpm for 15 min. The solution was poured away and the nanoparticles were redispersed in 40 ml of a water/ethanol mixture (3:1). The solution was centrifuged again and poured away. The precipitate was twice redispersed in 30 ml H_2O, centrifuged and poured away. Afterwards the particles were freeze-dried overnight. A light green powder was obtained.

Synthesis of β-$Co(OH)_2$

β-$Co(OH)_2$ was synthesized by a similar process described by Ma et al. [15] based on homogeneous precipitation. Cobalt-II-chloride and Hexamethylentetramine (HMT) were dissolved in a 500 mL three-neck-flask of deionized ultra-pure water to concentrations of 5 mM and 90 mM, respectively. To remove all Oxygen in the solution, this was bubbled for 30 minutes with Nitrogen. After that, the solution was heated at 110 °C under reflux, continuous stirring and an inert Nitrogen atmosphere. After 5 hours of heating a pink-colored solid product was recovered by centrifuge (8500 rpm, 15 min). The precipitation was washed two times with a water-ethanol mix (3:1) and two times with water. The product was freeze-dried overnight.

Bulk characterization

TEM images were acquired with a FEI TECNAI G2 20 S-TWIN transmission electron microscope with LaB6 cathode. The microscope operated at an accelerating voltage of 200 kV. Inductively- coupled plasma optical emission spectroscopy (ICP-OES) data were obtained by a 715-ES-ICP analysis system (Varian).

Ink preparation

For in situ WAXS a catalyst ink was prepared. 1.97 mg of catalyst was weighted in a glass vial. Then 200 µl of MilliQ water, 800 µl of isopropanol and 5 µl of Nafion solution (5 wt%) were added. The solution was ultrasonicated with a 1/8 in microtip sonifier for 30 min. An appropriate volume of ink were drop casted on a previously polished and cleaned glassy carbon (GC) electrode (5 mm of diameter) and dried in an oven at 60°C for ~7 min. Different targeted catalyst loadings were investigated for in situ WAXS measurements: 100 µg cm^{-2}, 33 µg cm^{-2} and 20 µg cm^{-2}. While for RDE measurements, the loading was 100 µg cm^{-2}.

The GC disks were polished manually with a 1.0 and 0.05 mm micropolish alumina suspension for ~3 min each before each catalyst coating. After polishing, the disks were cleaned three times by ultrasonication in water, acetone, and water and finally dried with a nitrogen flow.

RDE measurements

RDE electrochemical experiments were performed in a three-compartment glass cell with a rotating disk electrode (RDE, 5 mm in diameter of GC, Pine Instrument) and a potentiostat (Gamry) at room temperature. A Pt-mesh and a Hydroflex reversible hydrogen electrode (RHE, Gaskatel) were used as counter electrode and reference electrode, respectively. The counter electrode was placed in a compartment that was separated by a fine-porosity glass frit from the working electrode compartment and a Luggin capillary was used for the reference electrode. The electrolytes were prepared with KOH pellets (semiconductor grade, 99.99% trace metals basis, Aldrich), and MilliQ water. The electrolyte was purified by using sacrificial $Co(OH)_2$ for the Co based samples and $Ni(OH)_2$ for the Ni based samples, according to the procedure described by Boettcher and coworkers,[16,17] to remove Fe impurities which affect the activity of $Ni(OH)_2$ and $Co(OH)_2$. Briefly: First 2 g of $Ni(OAc)_2{\cdot}4H_2O$ were dissolved in 10 ml deionized water. By adding 20 ml 1 M KOH, $Ni(OH)_2$ precipitated. The solution was agitated and then centrifuged for 15 min at 8500 rpm. The particles were washed three times with a mixture of 20 ml H_2O and 2 ml 1 M KOH. Afterwards the solution was poured away and 50 ml 1 M KOH were added to the particles. The solution was agitated for at least 10 min before resting overnight. Then the mixture was centrifuged. The supernatant was decanted through a filter paper into a clean tube and stored until use. Similarly, $Co(OH)_2$ was precipitated from 1 g of $Co(NO_3)_3$ with 10 mL 0.1 M KOH and washed three times with 10 mL 1 M KOH, centrifugated and decantated. The so washed $Co(OH)_2$ was added to 50 mL 1 M KOH. The suspension was standing overnight in the fridge and centrifuged and decanted next day. For the measurements, the electrolyte was diluted to 0.1 M.

All electrochemical measurements were carried out in N_2-saturated and rotation rate of 1600 rpm and repeated at least 3 times. The current density values reported are normalized by the geometric area (0.196 cm^2). Internal resistance (iR) correction was applied after the measurements by using the value of resistance obtained during electrochemical impedance spectroscopy (EIS). All the potentials reported are iR-corrected, unless otherwise stated. Cyclic voltammetry (CV) was conducted as an activation treatment at the sweep rate of 50 mV s^{-1}. The CVs were performed by cycling 50 times the (not iR-corrected) between 1 and 1.9 V versus RHE for NiFe LDH and $Ni(OH)_2$ and between 0.6 and 1.9 V_{RHE} for CoFe LDH and $Co(OH)_2$. After the CV, linear sweep voltammetry (LSV) measurements were conducted by sweeping the potential (not iR corrected) from 1.2 to 1.9 V versus RHE at a scan rate of 1 mV s^{-1}.

In situ WAXS

A home-made grazing incident cell (GID, Supplementary Figure 4) based on thin-layer concept and previously described in ref._ENREF_5[18] was used. A PEEK foil (Thickness: 0.006 mm, Goodfellow GmbH) covers the electrolyte compartment and is used as X-Ray window. The cell is tightened by screwing an aluminum annular plate on top of the PEEK foil with an o-ring to provide sealing. The

sample coated GC was mounted on a central cylindrical holder which is part of the GID cell body, contacted by a Ti spring and kept in place by applying vacuum though a cavity in the cylinder. An o-ring was used to guarantee appropriate sealing and avoid electrolyte leakage to the vacuum pump. 0.1 M KOH electrolyte was flown in the cell from a glass reservoir bottle by a peristaltic pump. The electrolyte was prepared by KOH pellets (semiconductor grade, 99.99% trace metals basis, Aldrich) and MilliQ water (~80 ml freshly prepared for each sample). The Counter electrode, a platinum wire, was placed in the electrolyte outlet, while a Ag/AgCl reference electrode (3M KCl, World Precision Instruments) was placed in a compartment next to the inlet. The reference electrode was calibrated for each beamtime by using a reversible hydrogen electrode (RHE) which was fabricated by bubbling H_2 over a polycrystalline Pt disk electrode in 0.1 M KOH. The reported current density values are normalized by the geometric area (0.196 cm^2). Internal resistance (iR) correction was applied after the measurements by using the value of resistance obtained during electrochemical impedance spectroscopy (EIS). All the potentials reported are iR-corrected and refer to RHE.

Investigation of crystal structure under electrochemical reaction conditions have been conducted at the ID31 beamline of the European Synchrotron Radiation Facility (ESRF) in Grenoble (Fr). Diffraction patterns were recorded using a monochromatized X-ray beam in the range 60 - 78 KeV (slightly difference due to different beamtimes: 77 KeV for all the samples apart NiFe LDH with loading 0.1 mg cm^{-2}, where it was 60 KeV and NiFe LDH with “collapsed film technique” where it was 78.1 KeV) focused to 20 × 5 μm^2 using a large area Pilatus3X CdTe 2M detector. In the text, the radial integration of the WAXS 2D image is discussed and analyzed. Diffraction patterns were corrected by the background, by subtracting a WAXS pattern obtained in a point where no sample was present, typically at edges of the GC. The working distance was calibrated using a CeO_2 standard (NIST SRM 674b). In the case of NiFe LDH, in order to enhance the signal at high q the electrolyte reservoir was lowered during some measurements at relevant potential values (specified in the text) such that the PEEK film almost collapsed on top of the catalyst leaving only an ultra thin electrolyte film and minimizing the scattering from the electrolyte respect to the diffracted sample peaks. Electrochemical protocols were applied using a SP-200 Potenstiotat (BioLogic Instruments). First a dry WAXS measurement was performed for each sample to verify the initial as prepared sample condition. After inserting the electrolyte a WAXS measurement was performed at 1 V_{RHE} and indicated as “wet state”. Electrochemical impedance spectroscopy (EIS) was performed at this stage at 1 V_{RHE} in the frequency range 1 Hz to 200 kHz. Then the potential was cycled 50 times at 50 mV s^{-1} from 1 V_{RHE} till OER potentials typically 1.7-1.6 V_{RHE} (activation treatment). During the activation treatment, a slow progressive growing of the redox features was observed for NiFe LDH, while for CoFe LDH this growing was faster and a stable curve reached with few cycles. Interestingly, for CoFe LDH the first cycle show a big oxidation peak at large anodic potential (Supplementary Figure 10) which is not accompanied by a reduction peak of the same magnitude, indicating the occurrence of an irreversible oxidation. WAXS measurements were obtained at 1 V_{RHE} at the end of each of the first 10 cycles and then at the end of the 20th, 30th, 40th and 50th cycle. Finally a sequence of potential steps of ~ 10 minutes length were applied, from potential below the M(II) oxidation potential (with M = Ni and Co respectively for NiFe and CoFe LDH) to OER conditions and back to potential well below the potential for reduction of M to M(II). Linear sweep voltammetry at the scan rate of 360 mV min^{-1} was used to connect the various potentials in the step sequence. With the lower NiFe LDH loading the electrical contact was lost at 1.5 V_{RHE} and the sample was recontacted. For the “collapsed film” technique, the length of the potential steps was further extended to ~40 minutes and after that the reservoir bottle was lowered and WAXS measured. WAXS measurements were performed at the end of each potential step. In the case of CoFe LDH the potential was decrease till 0.5 V_{RHE}, where oxygen reduction reaction (ORR) current was observed, in order to verify if a very low potential would eventually revert (contract) the oxidized CoFe LDH to a state similar to the as prepared state. At high positive potential, formation of O_2 bubbles was observed. Some of them could become trapped between the sample and the PEEK window. If this happened excessively a loss in surface area could occur and less material could become available for redox processes. Therefore the permanence to O_2 condition and exposure to high anodic potentials was limited and in certain cases bubbles gently manually displaced by lowering of the PEEK foil before decreasing the potential. The 2D WAXS images recorded by the detector were then radially integrated using a pyFAI code [https://doi.org/10.1107/S1600576715004306]. The fitting of the (003) and (110) peaks was performed with the Origin software and using Pseudo-Voigt functions. A maximum

of two Pseudo-Voigt functions were necessary to fit some of the (003) peaks which clearly show asymmetric profile. To verify the above fitting, a more advanced fitting was then achieved by Rietveld refinement.[19]

Rietveld Refinement

Rietveld refinement was performed using the software Topas 4.2 by Bruker AXS Inc. For NiFe LDH measurements taken with the lowest loading (33 µg cm^{-2}) were analyzed in addition to the measurements taken on a similar sample where "collapsed film" technique was used. The hydrotalcite structure with space group R-3m was used as model for the NiFe LDH phases.[20] For high anodic potential two phases were necessary. The scattering at low q (small angle X-ray diffraction SAXS region) was subtracted by spline function. Despite subtraction of the WAXS pattern taken at background position (no sample), residual water and GC contributions remained. For low potentials, the GC contribution was fitted using three (hkl) phases with space group P63mc, while to take into account the water scattering three peaks were defined corresponding to the major water peaks. For high potentials, where multiple NiFe LDH phases were observed, it was necessary to further reduce the background by fitting with a spline function the broad water features and the scattering at low q and subtract a dry GC WAXS pattern to decrease the GC component. After this additional background subtraction, no water peaks were needed, but two (hkl) phases were still needed for residual GC contribution. For samples measured by "collapsed film" technique, a WAXS pattern of the electrolyte and a WAXS pattern of the dry GC were subtracted and the spline fitting was only necessary for the scattering at low q. Three (hkl) phases with space group P63mc were used to model residual GC contribution. For CoFe LDH, the LDH related signals were more intense and additional background subtraction was not performed. For the remaining background contributions 4 phases with space group P63mc were used for the GC, and 6 peaks for the water. Hydrotalcite structure with space group R-3m was used as model for the CoFe LDH phases, while a spinel phase, space group Fd3m, was added at potential ≥ 1.3 V_{RHE} to obtain a better fit. At these potentials the remaining α-phase is so small, that the lattice parameter *a* of this phase needed to be constrained. Due to the nanoplatelet morphology and the laminar structure of the LDH prone to possible exfoliation, it is highly luckily that the crystalline domains are not spherical. We have found that an anisotropic model for the coherent scattering domains was necessary to obtain a better fit. To take this into account we used AnisoCS script[21] in Topas, and a cylindrical shape was chosen as model of the LDH scattering crystallites, for both NiFe LDH and CoFe LDH.

DEMS

Differential electrochemical mass spectrometry (DEMS) measurements were performed using dual thin-layer electrochemical flow cell with design partly reported elsewhere[22]. The gas products are introduce into ion source throughout a differential pumping composed by two turbomolecular pumps (HiPace 80) operating under 10^{-3} and 10^{-6} mbar. The mass spectrometer consists of a Prisma™ quadrupole (QMS 200, Pfeiffer-Vacuum). The electrolyte was separated from the vacuum using a 150 µm thick hydrophobic PTFE membrane (Cat. No. PF-003HS for Cobetter® porous size 30 nm). Working electrodes were prepared by drop-casting 5 µL of a catalyst ink on mechanically polished glassy carbon disk (Ø = 10 mm, HTW GmbH) with exposed area of 0.283 cm^2. A Pt-mesh was used as counter electrode connected to tangential channel to main inlet channel and a leak-free Ag/AgCl reference electrode for potential control. Measurements were recorded on a Biologic SP-200 potentiostat in Nitrogen saturated electrolyte 0.1M KOH. The electrolyte flow speed was set at the out let flow cell with a needle valve with 5µL.s^{-1} and controlled during experiments with a liquid flow sensor Sensirion SLQ-QT500. Measurements were afterwards corrected for the uncompensated ohmic resistance determined by electrochemical impedance spectroscopy. The WE and Teflon gasket contact were purged with N_2 in order to avoid environment air into the reaction compartment or leakage into the vacuum. The cyclevoltammetric mass spectrometer (MSCV) curves were recorded during OER regime in selected potential windows at a scan-rate of 10 mV.s^{-1} while recording the ion current for O_2 (m/z = 32). To assure optimized detection of products during potential cycling as assessed with mass spectrometer, the ion source was calibrated using calibration protocol presented at Quadera software. The calibration mass spectrum and chamber baked before experiments allowing us achieve background stability and reproducibility. The molecules at ion source are bombarded by electrons having energies

of about 80 eV. The amplification of the secondary electron multiplier (SEM) was setup to be around 3 orders of magnitude compare to Faraday detector.

Ex situ sXAS

Ex situ sXAS were performed at the ISISS beamline at BESSY II synchrotron radiation facility of the Helmholtz Zentrum Berlin. NiFe LDH and CoFe LDH were deposited on the GC using standard procedure. As prepared samples were compared with samples that were subjected to electrochemical activation (50 CVs in 0.1 M KOH in the potential range 1-1.7 V_{RHE}). For the sXAS measurements, NiO, CoO, CoOOH and Fe_2O_3 have been used as reference samples. The spectra were collected in the total electron yield mode.

Operando XAS

Operando XAS measurements were performed at the BL22 CLAESS beamline at ALBA light source (Barcelona, Spain) in fluorescence mode using a silicon drift diode (SDD) detector. A home-made electrochemical cell was employed. A platinum mesh and leak free Ag/AgCl electrode were used as counter and reference electrode, respectively. The powder samples were deposited on graphite paper discs (Toray Carbon Paper TP-060, Quintech) by filtration from a slurry of the sample in ethanol containing Nafion (0.1 v/v %) as a binding agent. Carbon paper discs were mounted in the operando cell so that the unmodified side was facing out, while the side containing the catalyst layer was in contact with the electrolyte. By this, intensity losses were avoided in the incident X-ray beam as well as fluorescence radiation emitted from the sample while passing through the electrolyte layer. Each of LDH catalysts was measured two times, at NiK or CoK and FeK edges, using a fresh sample for each measurement. XAS spectra were recorded as-prepared, at 1.0 V_{RHE} after activation by cycling potential 50 times at 50 mV s^{-1} from 1.0 V_{RHE} to 1.7 V_{RHE} , and at chronoamperometric conditions at 1.55 and 1.7 V_{RHE} (NiFe LDH) and 1.3, 1.6, 1.75 V_{RHE} (CoFe LDH). Initial processing of the XAS data was performed using the program Athena,[23] EXAFS analysis was conducted in VIPER software[24] using the FEFF8 code[25].

Computational details of α-NiFe and α-CoFe layered double hydroxides

To identify the possible configurations of the as-prepared NiFe and CoFe layered double hydroxides (LDHs) with M:Fe=3:1, we start by studying the structure of MgAl carbonate hydrotalcite, which is a representative of natural LDH phases with Mg:Al=3:1. MgAl carbonate hydrotalcite is known for its stoichiometry (i.e. $Mg_6Al_2CO_3(OH)_{16}\cdot 4H_2O$) and structure (3-layer rhombohedral polytype).[26,27] However, the atomic-scale details of Mg-Al relative position, and the configurations of intercalated CO_3^{2-} ions and water molecules are still unknown. Our calculations show that -Al-Mg-Al-Mg- configuration is ~0.3 eV more favorable than -Mg-Al-Al-Mg- configuration (Al-Al as nearest neighbor). Thus, we use the former as the framework to construct 3-layer MgAl carbonate hydrotalcite with one CO_3^{2-} ions and four water molecules randomly introduced between $Mg_6Al_2(OH)_{16}$ layers, which results in an overall stoichiometry of $(Mg_6Al_2CO_3(OH)_{16}\cdot 4H_2O)_3$. We firstly relax the structure and cell to local minima, and then employ ab initio molecular dynamics (AIMD) simulation to search for the possible configurations of intercalated water molecules and ions between layers. AIMD simulation is initiated by heating the system from 0 K up to 400 K, at which the canonical ensemble (NVT) simulations are performed with a total simulation time of 10 ps unless otherwise stated. The simulations are quenched down to 0 K every 1 ps. Then, both the local geometry and the cell are further optimized to get the representative configuration at each 1ps (see Supplementary Figure 26). The stability of each configuration relative to the initial configuration is plotted in Supplementary Figure 27. In general, the configurations after AIMD simulation are more stable than the initial configuration with the one at the 4th ps has the lowest relative energy.

We use the configuration at the 4th ps as the starting point to identify the possible structure of the as-prepared NiFe and CoFe LDH. Initial geometries are obtained by changing Mg^{2+} to Ni^{2+} or Co^{2+} and changing Al^{3+} to Fe^{3+}, then relaxing both the local geometry and the cell. For NiFe LDH, AIMD simulations are performed with the same protocols as that described above but with a slightly longer simulation time (12 ps in total) to search for possible configurations with lower energy. We have found that the configurations after extended AIMD simulation are only 30 meV more stable than the initial

configuration (see Supplementary Figure 28). Thus, we use the initial configurations of NiFe and CoFe LDHs as their representative structures.

To evaluate stability of LDH with respective to the component (hydroxy)oxides, water, and CO_2 in the atmosphere (400 ppm), we calculate the relative free energy of formation by considering the following process:

$$6A(OH)_2 + 2BOOH + CO_2(g) + 5H_2O(l) \rightarrow A_6B_2CO_3(OH)_{16} \cdot 4H_2O \quad (1)$$

Then the relative free energy of formation can be calculated with

$$\Delta G_{form} = \mu(A_6B_2CO_3(OH)_{16}.4H_2O) - 6\mu(A(OH)_2) - 2\mu(BOOH) - \mu(CO_2) - 5\mu(H_2O) \quad (2)$$

Where $A(OH)_2$ and BOOH are component (hydroxy)oxides with A^{2+} and B^{3+} oxidation state. The calculated relative free energy of formation of MgAl LDH, α-NiFe LDH and α-CoFe LDH are -0.66 eV, -0.17 eV and -0.05 eV, respectively. The exothermic process indicates that the formation of LDHs from component (hydroxy)oxides are favorable in water under ambient condition.

For the 3R configuration, intercalated water molecules and CO_3 ions between each layer have no symmetry as that for the main layer. By constraining water and CO_3 with same symmetry as the main layers, the supercell of the 3R configuration can be reduced to 1R type unit cell (see Supplementary Figure 27b). Our calculations have shown that, although 3R configuration can maximize the configurations entropy, the energy difference between supercell and unit cell is a few meV in general. Thus, to speed up the screening of γ-NiOOH, γ-NiFe and γ-CoFe LDH below, we will use 1R type unit cell in the calculations.

Computational details of γ-NiFe and γ-CoFe layered double hydroxides

To determine in-situ structure of NiFe LDH and CoFe LDH under OER condition, i.e. γ-NiFe and γ-CoFe, we start from screening the possible configuration of γ-NiOOH. For γ-NiOOH, it is well known that water and ions are intercalated between layers resulting in an interlayer spacing of ~7 Å, which is larger than that of anhydrous β-NiOOH (~4.8 Å), but the atomic scale structure is still unknown. Therefore, we have considered a serious possible structures with various amounts of water and ions intercalated between the NiOOH or NiO_2 layers, including NiOOH-(2×4)-nH_2O, NiOOH-(2×4)-CO_3-nH_2O, NiO_2-(2×3)-2K-nH_2O, NiO_2-(2×4)-2K-nH_2O, NiO_2-(2×3)-1K-nH_2O, NiO_2-(2×4)-1K-nH_2O, NiO_2-(2×4)-nH_2O with Ni nominal oxidation 3+, 3.25+, 3.67+, 3.75+, 3.83+, 3.875+, 4+, respectively. For each Ni oxidation state, ions and water molecules (n is from 2 to 8 in general) are randomly intercalated before the initial structural optimization. Then, for each stoichiometry, AIMD simulations are performed to identify the most stable configuration with the protocol described above but generally with a shorter simulation time (5 ps, instead of 10 ps unless otherwise stated), as the AIMD simulations on MgAL LDH and α-NiFe LDH have shown that configurations with reasonable stability can be achieved within 5 ps. As an additional example, the relative stability of NiO_2-(2×4)-2K-4H_2O from AIMD simulation is plotted in Figure 3a. Again, structures that are among the most stable configurations are achieved within 5 ps. The free energy of formation of each stoichiometry from anhydrous phases (NiOOH and/or NiO_2), water, CO_2 in atmosphere and/or K^+ in 0.1 M KOH are plotted in Figure 3b.

For NiO_2-(2×4)-2K-4H_2O as an example, the free energy of formation is calculated from the reaction

$$2NiOOH+6NiO_2+2H_2O(l)+2K^++2OH^- \rightarrow NiO_2\text{-}(2\times4)\text{-}2K\text{-}4H_2O \quad (3)$$

then

$$\Delta G_{from}=\mu(NiO_2\text{-}(2\times4)\text{-}2K\text{-}4H_2O)-(2\mu(NiOOH)+6\mu(NiO_2)+2\mu(H_2O)+2\mu(K^+)+2\mu(OH^-)), \quad (4)$$

which can be further normalized by the number of the metal atoms in the layer, i.e. 8 Ni in this case.

Due to the challenge of description ions in standard DFT calculations, the chemical potential of K^+ and OH^- are replaced with

$2\mu(K^+)+2\mu(OH^-)=\mu(K_2O)+\mu(H_2O)-4.8$ eV (5)

where -4.8 eV is the reaction free energy of

$K_2O+H_2O \rightarrow 2K^++2OH^-$ (6)

We can see that NiO_2-(2×4)-2K-nH_2O is generally more stable than the other structure series, in which NiO_2-(2×4)-2K-4H_2O is the most stable stoichiometry and one of two stoichiometries that have exothermic free energies of formation. For NiO_2-(2×4)-4H_2O-2K, two Ni cations (1/4) are in 3+ oxidation state with characterized magnetic moment of 0.9 μ_B, and six Ni cations (3/4) are in 4+ oxidation state with characterized magnetic moment of 0 μ_B (see Supplementary Figure 29 and Supplementary Table 5).

We use NiO_2-(2×4)-2K-4H_2O as the basis to study the structure of γ-NiFe LDH, by randomly replacing two non-nearest-neighbor Ni with two Fe (configurations with Fe-Fe as the nearest neighbor are > 0.4 eV less unfavorable), it results in 8 possible geometric configurations. The geometric structure and electronic structure (magnetic moment) of these configurations are given in Supplementary Figure 29. For all of them, two Ni cations (1/3) are in 3+ oxidation state with characterized magnetic moment of 0.9 μ_B, and four Ni cations (2/3) are in 4+ oxidation state with characterized magnetic moment of 0 μ_B. Based on the charge balance, two Fe cations are in 4+ oxidation. For all these configurations, the free energy of formation is exothermic, which suggests the favorable formation of γ-NiFe LDH from the component hydroxides, water and K+ ions in 0.1 M KOH. Also, the relative stability of 7 out 8 configurations are within 10 meV/M (80 meV/Ni_6Fe_2), in comparison with the most stable configuration, which indicates that many possible geometric combinations are possible under experimental conditions. We have used the most stable geometric configuration as the representative to study their OER performance.

Using the most favorable configuration as the starting point, we also have performed AIMD simulation to search for the other possible configurations, including $Ni_6Fe_2O_{16}$-2K-4H_2O, $Ni_6Fe_2O_{16}$-nH_2O and $Ni_6Fe_2O_{16}H_8$-CO_3-nH_2O. We have found that $Ni_6Fe_2O_{16}$-2K-4H_2O configuration obtained from NiO_2-(2×4)-2K-4H_2O is among the most stable $Ni_6Fe_2O_{16}$-2K-4H_2O configurations during the AIMD simulation, and the stability of $Ni_6Fe_2O_{16}$-nH_2O and $Ni_6Fe_2O_{16}H_8$-CO_3-nH_2O are similar to NiO_2-(2×4)-nH_2O and NiOOH-(2×4)-CO_3-nH_2O, i.e. ~0.15 eV/M (or ~1.2 eV/Ni_6Fe_2) less favorable. Additional configurations are also considered by adding or removing 1H atoms from of $Ni_6Fe_2O_{16}$ and $Ni_6Fe_2O_{16}H_8$ layers, respectively. While it does not noticeably influence the stability of $Ni_6Fe_2O_{16}$-nH_2O and $Ni_6Fe_2O_{16}H_8$-CO_3-nH_2O, it destabilizes $Ni_6Fe_2O_{16}$-2K-4H_2O (~0.2 eV/H). Thus, it is reliable to study the γ-phase of NiFe LDH based on the most stable NiO_2-(2×4)-4H_2O-2K, and $Ni_6Fe_2O_{16}$-2K-4H_2O is the most stable stoichiometry. We also have used NiO_2-(2×4)-4H_2O-2K as the basis to study the structure γ-CoFe LDH. For γ-CoFe LDH, there are four Co in 3+ oxidation state with characteristic magnetic moment of 0 μ_B, and two Co in 4+ oxidation state with characteristic magnetic moment of 1.1 μ_B, instead of two Ni in 3+ oxidation and four Ni in 4+ oxidation state for γ-NiFe LDH (see Supplementary Figure 30). It is worth noting that, for all 8 geometric configurations, the free energy of formation is exothermic, which suggests the favorable formation of γ-CoFe LDH from the component hydroxides, water and K+ ions in the electrolyte. Also, the relative stability of 5 out 8 configurations are within 10 meV/M (80 meV/Ni_6Fe_2), in comparison with the most stable configuration, which indicates that many possible geometric combinations are possible under experimental conditions. We have used the most stable geometric configuration as the representative to study their OER performance.

The necessity of considering steady-state surface configuration and self-consistent reaction mechanism

To illustrate the importance of considering the self-consistent Mars van Krevelen mechanism, which is derived from the steady-state configuration under OER conditions from surface diagram analysis, we have calculated the reaction free energies of conventional mechanism on Fe site of two artificial surface models: the pristine (01-10) surface and the steady-state configuration that is not relevant to OER but at the potential < 1 V. The latter is equivalent to saturating surface O sites of the pristine (01-10) surface with hydrogen, which is accompanied by the reduction of surface Fe^{4+} to Fe^{3+}. Based on the intrinsic

magnetic moment, we deduced that the corresponding Fe sites are in +4 and +3 oxidation state, respectively for the two configurations, and the corresponding surface Ni sites are in +3 and +2, oxidation state. It is worth noting that, [a] based on previous Mössbauer spectroscopy and our calculations, Fe sites are in 4+ oxidation state under OER condition; [b] in addition to the reduction of Fe^{4+} to Fe^{3+}, hydrogen saturation of surface O sites also leads to the reduction of surface Ni^{3+} to Ni^{2+}, while it is well known that Ni cations are in mixed Ni^{3+} and Ni^{4+} oxidation states under OER condition. Thus, based on these considerations, in comparison with the hydrogen saturated pristine surface, the unmodified pristine surface seems to be a more reasonable model under OER conditions. Following the conventional mechanism, however, the calculated overpotential on the pristine surface (Fe^{4+} site) is 1.32 V (Supplementary Figure 36), which is about 1 V higher than experimental values at 10 mA cm^{-2} (~0.35 V in current work), while that from hydrogen saturated surface (Fe^{3+} site) is 0.26 V (Supplementary Figure 37), which is, on the other hand, semi-quantitatively in agreement with the experimental values. We derive two conclusions based on such a test: [I] The electronic structure (oxidation state) of the active site has a dramatic influence on the reaction free energy and overpotential; [II] A seemingly reasonable representative of the surface model, i.e. the unmodified pristine surface, may lead to unreasonably high overpotential, while it is possible to reproduce the experimental overpotential using an artificial model which appears in contrast with other experimental parameters. The counterintuitive results illustrate the importance of considering steady-state surface configuration through surface phase diagram analysis and self-consistent reaction mechanism as conducted in the current work.

Physicochemical characterization of as prepared MFe LDHs

NiFe and CoFe LDH catalysts can be obtained by co-precipitation, homogeneous precipitation and several other methods including electrodeposition and phase transformations.[17,28-33] We have chosen for the synthesis of NiFe LDH a well known method which produces crystalline nanoplates which were reported to be highly active for the OER.[14,34] In the case of CoFe LDH, a variation of the method reported for NiFe LDH was necessary in order to obtain the desired phase. Both the synthesized catalysts show the morphology of thin nanoplates (Supplementary Figure 3a and b) which is typical of LDH materials, with the difference that the nanoplates of NiFe LDH are larger and, excluding fragments, with a better defined hexagonal shape. In addition, small FeOxHy nanoparticles are observed in transmission electron microscopy (TEM) and are expected to be present, as previously reported,[7,14,34] due to the low pKa of Fe^{3+}. Compositional studies on NiFe oxyhydroxides have shown that the higher amount of FeO_xH_y domains at high Fe contents have a negative effect on the OER activity,[7] probably due to their low electrical conductivity, which makes them bad OER catalysts.[7,35] Therefore, they are not expected to be responsible for the high OER activity of our catalysts.

WAXS performed on the as prepared samples in the dried state (Supplementary Figure 3c) confirmed that both materials crystallized in the LDH phase, with their patterns matching the one of hydrotalcite. The well defined crystal structure makes these catalysts a good system for fundamental studies aiming at unrevealing structure activity relationships which might then be adapted and extended to more amorphous materials with similar composition and likely similar local structure.[36] This approach can be considered complementary to investigations using thin films, which can also provide fundamental insights i.e. thanks to the minimization of the electrical transport length through the film, but are generally X-ray amorphous and so some information are not easily extractable.[16,36-38] Investigation of well defined single crystal surfaces will also be beneficial for this goal, i.e. helping identifying the active sites, but such systems are not available at the moment. The (003) and (110) reflection in the WAXS pattern are particularly interesting since the first is associated to the interlayer distance (equal to one third of the lattice parameter c) and the second is associated to a distance which is half the intralayer distance of two metal atoms (equal to the lattice parameter a).

In addition to the LDH crystalline phase a specific composition has been targeted for the two materials. Inductively coupled plasma optical emission spectroscopy (ICP-OES) confirmed that both materials have similar metal atomic composition, with M/Fe ratio ~3.55 for NiFe LDH and ~3.33 for CoFe LDH (Fe at% of ~22 and ~23, respectively). These values were chosen since often reported in the range where the lowest OER overpotentials at fixed current densities are obtained.[16,17,33,38]

The results of the characterization confirmed that both synthesized materials show similar features in crystal structure, morphology and composition, and therefore were selected as candidate materials for

comparing their electrochemical behavior under applied anodic potential where OER occurs and investigate structure-activity relationship.

Operando XAS measurements: the consistency with the WAXS measurements

To confirm the incomplete phase transition of MFe LDH observed in our WAXS measurements, *operando* XAS measurements (Supplementary Figure 16-25) were carried out at the Ni K-, Co K- and Fe K-edges (8333, 7709, and 7112 eV, correspondingly). For NiFe LDH, Ni K-edge X-ray absorption near-edge spectra (XANES) show a well-defined pre-edge feature at 8330.6 eV, characteristic of Ni ions in octahedral coordination,[39] which persists through all reaction conditions (Supplementary Figure 16a, 17a and 18a). At the same time, the maximum of the first feature above the edge, so-called white line, shifts from 8348.4 to 8348.7 eV and diminishes under oxidation potential. The positive shift indicates the formation of higher oxidation states under oxygen evolution conditions. However, the shift is so small that the average oxidation state is lower than 3+, which is consistent with the limited oxidative phase transition of α-NiFe LDH (Ni^{2+}) in our WAXS measurement. As we discussed in the main text, the limited phase transition is likely because some nanoplates in the catalyst film are not electrochemically accessible, e.g. not in contact with the electrolyte or with the external electrical circuit. This explanation is supported by our loading-dependent *operando* WAXS study, which shows that the ratio of phase transition decreases with the increasing loading and so the presumably decreasing accessibility to the electrolyte (see Supplementary Figure 15). In addition, this argument is also consistent with a XAS study by Görlin et al, which showed that carbon-supported NiFe catalysts with high electrolyte accessibility have a higher ratio of phase transition than the unsupported samples that are likely less electrochemically accessible.[40] Therefore, in agreement with the DFT calculations, the oxidation state from XAS measurement is a weighted average of Ni^{2+}, Ni^{3+} and Ni^{4+}.

The limited oxidative phase transition is also evidenced in the extended X-ray absorption fine-structure spectrum (EXAFS) (Supplementary Figure 16b, Supplementary Table 1). As the potential increases, there are only small changes of main scattering shells. However, the oxidative phase transition is still evidenced by the contraction (though small) at the most sensitive first scattering shell (Ni-O bond distance) and the appearing of a minor second scattering shell with a shorter Ni-M bond distance than that in the main second scattering shell Ni-M bonds. On the other hand, the slight increase of the main Ni-M bond distances is likely an artifact in our one-phase FFT model due to the overlapping effect of multiple Ni containing phases. The limited phase transition of α-NiFe LDH is also evidenced from the corresponding Fe K-edge XAS spectra (Supplementary Figure 16b and the details of the EXAFS fitting are provided in Supplementary Table 2), i.e. there are only small changes of both the oxidation state and the bond length under reaction condition. Finally, it is worth mentioning that the local environment of both Ni and Fe returns in a reversible way to its initial state as soon as the resting potential is applied, which is also consistent with the reversibility observed in WAXS measurement. In summary, the *operando* XAS results for NiFe LDH are in general consistent with what is observed in our *operando* WAXS measurement.

The Co K-edge XANES (Supplementary Figure 16c and 19-20) spectrum of the as-prepared CoFe LDH shows a pre-edge feature at 7709.7 eV and a sharp intense white line at 7727.8 eV, clearly indicating a dominating contribution of Co^{2+} in octahedral coordination. Under reaction conditions the pre-edge feature as well as the white line shift to higher energy (7710.3 and 7730.4 eV, correspondingly) while the white line diminishes, resembling CoOOH[41] and our Co_3O_4 reference (Supplementary Figure 20), confirming larger ratio of phase transition of α-CoFe LDH than α-NiFe LDH as observed in our WAXS measurement. This is further evidenced by the corresponding EXAFS spectrum (Supplementary Figure 16d) which undergoes more drastic changes as compared with the NiFe LDH discussed above. Likewise, *operando* Fe K-edge XAS (Supplementary Figure 16c-d) data reveal significant changes in both the chemical state and the local environment of iron. The Fe K-edge XANES spectrum of the as-prepared CoFe LDH resembles the one of NiFe LDH, suggesting Fe^{3+} in octahedral coordination (Supplementary Figure 16 and 21). The spectra measured under reaction conditions however exhibit lower white line shifted to higher photon energy. Such behavior is similar to Fe K-edge XANES spectra earlier reported for the $La_{1-\delta}Sr_{\delta}FeO_4$.[42] The changes observed in CoFe LDH are however less pronounced, indicating only partial oxidation change of Fe^{3+} to higher oxidation states, which is consistent with the partial phase transition observed in our WAXS measurement (i.e. Supplementary Figure 6b, 7b, 8b and 9b). The

EXAFS spectrum indicates that the Fe-O distance changes from 1.99 Å in the as-prepared sample to 1.92 Å at higher potentials, while the metal-metal distance contracts to 2.80 Å under OER conditions (Supplementary Table 4). The results are well consistent with the calculated local structural parameters of the γ-CoFe LDH, which further concludes the occurrence of a larger fraction of oxidative phase transition for α-CoFe LDH than α-NiFe LDH, as observed in WAXS measurement. We note that, although the trends are the same, the quantitative ratio of the phase transition in *operando* XAS and WAXS experiment are not necessarily the same, due to the different electrochemical environment used in those measurements such as the cell configuration and support used. We further note that, the Fe K-edge EXAFS spectra of CoFe LDH show an additional shoulder on the Fe-M peak at ca. 3.29 Å (uncorrected) that was not observed for NiFe LDH. An extra Fe-Co path from cobalt ferrite with a spinel structure needed to be included to get the best fit of the EXAFS spectra. This is in agreement with the addition of a spinel phase in the Rietveld refinement of the WAXS patterns. We note that Co-based spinel phases are expected to be less active than oxyhydroxide phases. It was shown previously that a reversible transformation occurs on the surface of Co_3O_4 catalysts, where an amorphous oxyhydroxide phase evolves during OER.[43,44] _ENREF_32 The formation of an amorphous oxyhydroxide phase on the surface of binary Co-Fe spinel OER catalysts was also shown by Calvillo et al.[45] This demonstrates that the transformation is not only limited to the Fe-free Co spinel. Therefore, these works proved that the Co(Fe)-based oxyhydroxide is the real OER active phase, even in the presence of spinel phases.

Discussion on coordination number changes from operando XAS analysis

As prepared NiFe LDH showed coordination numbers (CN) for Ni-O and Ni-M distances close to six, as expected from the hydrotalcite–like structure. As the potential increases, the apparent Ni-O and Ni-M CN decreases reaching 4.2 and 4.3 at 1.7 V_{RHE}, respectively. The apparent changes in the coordination numbers indicate lower coordinated Ni ions leaving ideal octahedral coordination. XANES spectra of the Fe K-edge dominantly point towards Fe^{3+} in octahedral coordination as indicated by a low intensity pre-edge peak at 7112 eV, however the coordination numbers are 4.8 and 4.0 for the first two coordination shells, in contrast to 6-fold coordinated Fe in γ-FeOOH or in hydrotalcite-like structure. A possibility might involve contributions from small XRD-amorphous FeO_xH_y nanoparticles observed in TEM. Under reaction conditions, the oxidation state and local symmetry of iron sites seem to remain unchanged, as seen from XANES spectra, despite the fact that both Fe-O and Fe-M CN decrease to 4.2 and 2.9, correspondingly. The changes in CN for both Ni and Fe were reversible upon returning the potential to 1 V_{RHE}, the resting state. The fitted coordination numbers of the CoFe LDH EXAFS spectrum are as low as CN_{Co-O} = 4.4 and CN_{Co-M} = 2.4. Since the Debye-Waller factor of the Co-O path is considerably large, one can suggest the presence of an amorphous Co phase with lack of long-range order. There is hardly any significant change in the positions of the EXAFS peaks upon applying higher potentials, they grow however in intensity, reaching CN_{Co-O} = 4.8 and CN_{Co-M} = 3.6. Similarly, the Debye-Waller factor of the Co-O path reduces considerably, which could indicate ordering of the suggested amorphous phase. The Fe-O CN after treating the catalysts by cycling potential and applying resting potential decreases from 4.0 to 3.0 but remains almost unchanged at higher potential values. The coordination number of the Fe-M path in contrast increases stepwise and reaches a value of 4.4 under OER conditions.

Discussion on intermediate phases

For NiFe LDH, interestingly, not all the α-phase transforms into γ, but the remaining part slightly contract. We call this oxidized phase α'-NiFe LDH. Since the contribution of the γ phase grows for lower loading respect to α', it is possible that for thin and ultra thin films as in ref. [7], γ-NiFe LDH is the only phase present while for thick electrodes, for example the one used in the present work, both phases will be present under OER. We tentatively explain α'-NiFe LDH as a part of the material that cannot fully charge, and so as an intermediate phase between α and γ, strongly dependent of preparation methods. Indeed these two phases, as well as the $Ni(OH)_2$ phases in the Bode Diagram, are intended as limiting forms with a range of intermediate compositions possible, as discussed by Doyle et al.[46] This intermediate phase is not only evident in the analysis of the (003) peak but also in the (110). In addition to a new broad peak which appears at much shorter distances (~2.85 Å), the (110) slightly contracts as the potential is increased above Ni(II) oxidation (Figure 2 and Supplementary Figure 13), contraction which can be associated to the emergence of the α'-NiFe LDH phase.

Size of coherently scattering domains by Rietveld refinement

An estimation of the size of the various phases, or better of their coherently scattering domains, can be obtained from the r_x, r_y, r_z radii used in the anisotropic model implemented in the AnisoCS script in Topas.[21] In the cylindrical model used the diameter of the circular base is given by twice the radius r_{xy} ($r_{xy}=r_x=r_y$), while the cylinder height is equal to twice r_z. The z axis in the model is taken parallel to the (001). No particular trend was observed as a function of the applied potential within the experimental error. Therefore we will provide the averaged diameter x height ($2*r_{xy}$ x $2*r_z$) values obtained over several potentials. For NiFe LDH the dimensions are ~20 ± 7 x 6 ± 1 nm for the α and ~2 ± 1 x 4 ± 1 nm for the γ phase. For CoFe LDH, 13 ± 4 x 8 ± 3 nm for the α and 2 ± 1 x 7 ± 1 nm for the γ phase. For $Co_{3-x}Fe_xO_4$, a sphere was used as a model and the obtained diameter is ~7 ± 2 nm. In both cases the dimensions of the domains of the α phases in the xy plane are much smaller than the nanoplatelet size that is observed in TEM, ~ 400 nm for NiFe LDH and a broad distribution around ~100 nm for CoFe LDH. This confirms that the nanoplatelets are polycrystalline. The γ phases of the LDHs are in general smaller than the α phases. In addition, the height of the cylindrical domains is smaller than their diameter for the α phases of both materials, while the opposite is observed for the γ phases. The height of the γ phases is almost as large as the height of the α phases, but the diameter of the γ phases is much smaller than the diameter of the α phases. A possible explanation for this is that domains at the edge of the nanoplatelets are in general smaller, since grown later in the synthesis, and these are the first to change into γ when the potential is increased, in agreement with calculations that suggest the edges as most reactive sites.[7,47,48] The domain size in our solvothermally synthesized α-NiFe LDH samples are roughly ~4 times bigger compared to the values obtained by pair distribution function (PDF) analysis of ex-situ data measured on electrodeposited NiFe (oxy)hydroxide which is typically referred to as amorphous.[36]

Ex situ sXAS

The irreversible transformations occurring in CoFe LDH in contrast to the more reversible behavior of NiFe LDH, has been also verified by ex situ soft XAS (sXAS) at the Ni, Co, Fe L-edge before and after electrochemical cycling (Supplementary Figure 11). By comparing the spectra with the ones of reference samples, average oxidation states of the metals can be deduced. As prepared NiFe LDH is composed of Ni in 2+ oxidation state and Fe in high spin 3+. No big changes are evident after activation, only a very small increase in intensity of the higher energy contribution for both metals, which might indicate a small portion of metal centers in higher oxidation state.[49] However, we emphasize that these changes are very small, and the interpretation complicated (i.e. defects should be taken into account). As prepared CoFe LDH has mostly octahedral Co^{2+} sites with some smaller Co^{3+} contribution, and Fe is in high spin 3+. While the as prepared phase of CoFe LDH is similar in terms of metal oxidation states to NiFe LDH, the Co L-edge of oxidized CoFe LDH compares well with the one of CoOOH, that is composed of Co atoms in octahedral 3+ state. After activation, the Fe is still in high spin 3+ but developed a shoulder at high photon energies that might indicate a small amount of Fe^{4+}. We conclude that after activation, almost all the metal centers of NiFe LDH show the same oxidation state of the as synthesized, while Co centers in CoFe LDH change significantly from 2 to 3 and Fe remains for the majority of 3+. Small shoulders at the high energy side of Fe L-edge grow after activation, and, despite different interpretations are possible, might indicate that some Fe centers are oxidized to higher oxidation states. Fe(IV) at the surface will most likely be reduced back by moisture to Fe(III), therefore if Fe(IV) is responsible for this shoulder, it is somewhere inside the bulk and most likely these centers are not the one involved in the catalysis. Nonetheless we notice that Fe(IV) centers have been observed in in situ Mössbauer spectroscopy[50-53] and XAS[8,49] in NiFe oxyhidroxides and it is under debate[33] _ENREF_40 if it is the active site or part of the active catalytic cluster unit for these catalysts. The ex situ O k-edge was also investigated for the same catalyst conditions used in the metal L-edge experiments as complementary tool to support the metal sXAS data (Supplementary Figure 12). The range centered at 530 eV and extending till ~535 eV is particularly interesting as attributed to the transition from O1s to unoccupied metal 3d orbitals hybridized with O2p.[54] Considering the multicomponent systems and presence of multiple phases the interpretation of the O k-edge is particularly complicated, therefore we will limit the analysis to general observations. For NiFe LDH, two peaks are observed at 531.6 and 534 eV, and a very weak bump at ~529 eV. The intensity of the two peaks switches after activation, with the low energy peak growing respect to the latter. By comparison of the spectrum with the one reported for chemically prepared γ-NiOOH,[37] which shows an intense peak around ~529 eV, we exclude a consistent

contribution from high valent Ni(III or IV) in our material in the as prepared and after activated states. The interpretation of the peak doublet and its switching is particularly difficult. O k-edge spectra of FeOOH phases measured by electron energy loss show a main peak centered at energy loss ~530 eV with a low energy shoulder,[55] while in O k-edge sXAS of $Ni(OH)_2$ a peak is observed at ~534 eV and a weaker peak at energy slightly below 530 eV.[37,49] While we can tentatively assign the peak at 534 eV observed in our material mainly to Ni 3d contribution, we do not have explanation for the switching and for the peak at 531.6 eV and DFT calculations are necessary. For CoFe LDH, a big change is observed. The onset of the O k-edge shift to lower energy and the new peak at ~ 530.5 eV is much more intense than the feature of the as prepared sample. The new peak is also clearly composed of several contributions, notably a shoulder at low energy at ~ 529 eV. The increase in intensity is in agreement with increased availability of empty d states and with metal center oxidation.[37,54] In comparison with the Co L-edge, we attribute this peak mostly to formation of Co(III).

Determination of turnover frequency and specific activity

The required overpotential to obtain 10 mA cm^{-2} is used as activity metric to show the activity trend among the catalysts. However, changes in active site density and surface area might affect the activity trend and therefore intrinsic activity should be estimated as comparison. The turnover frequency (TOF) is a widely accepted metrics for intrinsic activity in catalysis community. However, it relies on accurate determination of the nature and number of the active sites, including the distinction between surface and bulk sites and between active and non-active sites. This is typically considered hard to impossible to achieve for the family of MFe oxyhydroxide catalysts. This is why, in the open literature, a conservative TOF value is often cited, obtained by normalization with the total number of bulk metal sites (see work by Gong et al.,[34] Burke et al.,[17] Görlin et al.,[1] ...). This metric is conservative and is not prone to erroneous estimation of the number of metal centers at the oxide surface by assuming that all metal centers are active, which is probably not the case. For this reason, we decided to provide the TOF of total metal sites (TOF_{TOTM}) obtained by ICP-OES. Another important intrinsic activity metrics is the specific activity obtained by normalization of the current by the electrochemically active surface area (ECSA). In this case, the determination of the ECSA is particularly controversial and under debate for metal (oxy)hydroxide catalysts. The method estimating double layer capacitance (C_{DL}) by cyclic voltammetry which was proposed for metal oxides[56] has been shown to not be trustable for these materials,[57] due to the insulating character in potential region free of Faradaic currents. Therefore methods based on electrochemical impedance spectroscopy (EIS) at potentials above the M(II) oxidation seems more suitable. In this case, the impedance data needs to be fitted with an equivalent circuit. Batchellor et al. used a simplified equivalent circuit (Randles circuit) to extract the C_{DL} while suggesting that more complicated circuits might be more appropriate.[57] We based our equivalent circuit (see Supplementary Figure 1) on previous models proposed by Watzele and Bandarenka[58] and by Lyons and Brandon.[59] In these and our model, the capacitance considered is the one associated to adsorbed OER intermediates, instead of C_{DL}. For both C_{DL} and C_a capacitances we used constant phase elements (CPEs). The ECSA is calculated from the CPE_a after dividing by the specific area capacitance (C_s). For the latter we used the value of 0.3 mF cm^{-2} obtained by Watzele et al. for $Ni(OH)_2$,[58] since values for each catalysts were not available. We note that McCrory et al. also used a single value for their benchmarking study for similar issue.[56] Our EIS was performed at 1.6 V_{RHE}, for the electrical conductivity reason specified above. For both the TOF and specific activity the current at the overpotential of 350 mV is used for the calculation, as often reported in the literature. The comparison of the activity metrics is shown in Supplementary Figure 2.

Supplementary References

1 Gorlin, M. *et al.* Oxygen evolution reaction dynamics, faradaic charge efficiency, and the active metal redox states of Ni-Fe oxide water oxidation electrocatalysts. *J Am Chem Soc* (2016).

2 Gorlin, M. *et al.* Tracking Catalyst Redox States and Reaction Dynamics in Ni-Fe Oxyhydroxide Oxygen Evolution Reaction Electrocatalysts: The Role of Catalyst Support and Electrolyte pH. *J Am Chem Soc* **139**, 2070-2082 (2017).

3 Dresp, S. *et al.* Direct Electrolytic Splitting of Seawater: Activity, Selectivity, Degradation, and Recovery Studied from the Molecular Catalyst Structure to the Electrolyzer Cell Level. *Advanced Energy Materials* **8** (2018).

4 Gorlin, M. *et al.* Formation of unexpectedly active Ni-Fe oxygen evolution electrocatalysts by physically mixing Ni and Fe oxyhydroxides. *Chem Commun* **55**, 818-821 (2019).

5 Smith, R. D. L. *et al.* Geometric distortions in nickel (oxy)hydroxide electrocatalysts by redox inactive iron ions. *Energ Environ Sci* **11**, 2476-2485 (2018).

6 Gonzalez-Flores, D. *et al.* Nickel-iron catalysts for electrochemical water oxidation - redox synergism investigated by in situ X-ray spectroscopy with millisecond time resolution. *Sustain Energ Fuels* **2**, 1986-1994 (2018).

7 Friebel, D. *et al.* Identification of Highly Active Fe Sites in (Ni,Fe)OOH for Electrocatalytic Water Splitting. *J Am Chem Soc* **137**, 1305-1313 (2015).

8 Balasubramanian, M., Melendres, C. A. & Mini, S. X-ray absorption spectroscopy studies of the local atomic and electronic structure of iron incorporated into electrodeposited hydrous nickel oxide films. *J Phys Chem B* **104**, 4300-4306 (2000).

9 Huber, K. P. H., G. (Van Nostrand Reinhold Co, 1979).

10 Shimanouchi, T. (NSRDS-NBS 39, 1972).

11 Chase, M. W. *NIST-JANAF Thermochemical Tables*. 4th edn, (American Chemical Society, 1998).

12 Zeng, Z. & Greeley, J. Characterization of oxygenated species at water/Pt(111) interfaces from DFT energetics and XPS simulations. *Nano Energy* **29**, 369-377 (2016).

13 Wang, L. *et al.* Core–Shell Nanostructured Cobalt–Platinum Electrocatalysts with Enhanced Durability. *ACS Catal.*, 35-42 (2018).

14 Dionigi, F., Reier, T., Pawolek, Z., Gliech, M. & Strasser, P. Design Criteria, Operating Conditions, and Nickel-Iron Hydroxide Catalyst Materials for Selective Seawater Electrolysis. *Chemsuschem* **9**, 962-972 (2016).

15 Ma, R. *et al.* Topochemical synthesis of monometallic (Co2+-Co3+) layered double hydroxide and its exfoliation into positively charged Co(OH)2 nanosheets. *Angew Chem Int Ed Engl* **47**, 86-89 (2008).

16 Trotochaud, L., Young, S. L., Ranney, J. K. & Boettcher, S. W. Nickel-Iron Oxyhydroxide Oxygen-Evolution Electrocatalysts: The Role of Intentional and Incidental Iron Incorporation. *J Am Chem Soc* **136**, 6744-6753 (2014).

17 Burke, M. S., Kast, M. G., Trotochaud, L., Smith, A. M. & Boettcher, S. W. Cobalt-Iron (Oxy)hydroxide Oxygen Evolution Electrocatalysts: The Role of Structure and Composition on Activity, Stability, and Mechanism. *J Am Chem Soc* **137**, 3638-3648 (2015).

18 Bergmann, A. *et al.* Reversible amorphization and the catalytically active state of crystalline Co3O4 during oxygen evolution. *Nature Communications* **6**, 8625 (2015).

19 Rietveld, H. M. A profile refinement method for nuclear and magnetic structures. *J. Appl. Crystallogr.* **2**, 65-71 (1969).

20 Allmann, R. & Joepsen, H. P. Die struktur des hydrotalkits. *Neues Jahrbuch fur Mineralogie, Monatshefte* **1969**, 544 - 551 (1969).

21 Ectors, D., Goetz-Neunhoeffer, F. & Neubauer, J. A generalized geometric approach to anisotropic peak broadening due to domain morphology. *Journal of Applied Crystallography* **48**, 189-194 (2015).

22 Jusys, Z., Massong, H. & Baltruschat, H. A new approach for simultaneous DEMS and EQCM: Electro-oxidation of adsorbed CO on Pt and Pt-Ru. *J. Electrochem. Soc.* **146**, 1093-1098 (1999).
23 Ravel, B. & Newville, M. ATHENA, ARTEMIS, HEPHAESTUS: data analysis for X-ray absorption spectroscopy using IFEFFIT. *J Synchrotron Radiat* **12**, 537-541 (2005).
24 Klementev, K. V. Extraction of the fine structure from x-ray absorption spectra. *J Phys D Appl Phys* **34**, 209-217 (2001).
25 Ankudinov, A. L., Ravel, B., Rehr, J. J. & Conradson, S. D. Real-space multiple-scattering calculation and interpretation of x-ray-absorption near-edge structure. *Phys Rev B* **58**, 7565-7576 (1998).
26 MILLS, S. J., CHRISTY, A. G., GENIN, J.-M. R., KAMEDA, T. & COLOMBO, F. Nomenclature of the hydrotalcite supergroup: natural layered double hydroxides. *Mineralogical Magazine* **76**, 1289-1336 (2012).
27 De Roy, A. Lamellar Double Hydroxides. *Molecular Crystals and Liquid Crystals Science and Technology. Section A. Molecular Crystals and Liquid Crystals* **311**, 173-193 (1998).
28 del Arco, M., Trujillano, R. & Rives, V. Cobalt-iron hydroxycarbonates and their evolution to mixed oxides with spinel structure. *J Mater Chem* **8**, 761-767 (1998).
29 Ma, R. Z. *et al.* Synthesis and exfoliation of Co2+-Fe3+ layered double hydroxides: An innovative topochemical approach. *J Am Chem Soc* **129**, 5257-5263 (2007).
30 Ge, X., Gu, C. D., Wang, X. L. & Tu, J. P. Ionothermal synthesis of cobalt iron layered double hydroxides (LDHs) with expanded interlayer spacing as advanced electrochemical materials. *J Mater Chem A* **2**, 17066-17076 (2014).
31 Hadi, J., Grangeon, S., Warmont, F., Seron, A. & Greneche, J. M. A novel and easy chemical-clock synthesis of nanocrystalline iron-cobalt bearing layered double hydroxides. *J Colloid Interf Sci* **434**, 130-140 (2014).
32 Zhang, B. *et al.* Homogeneously dispersed multimetal oxygen-evolving catalysts. *Science* **352**, 333-337 (2016).
33 Dionigi, F. & Strasser, P. NiFe-Based (Oxy)hydroxide Catalysts for Oxygen Evolution Reaction in Non-Acidic Electrolytes. *Adv. Energy Mater.* **6**, 1600621 (2016).
34 Gong, M. *et al.* An Advanced Ni-Fe Layered Double Hydroxide Electrocatalyst for Water Oxidation. *J. Am. Chem. Soc.* **135**, 8452-8455 (2013).
35 Burke, M. S. *et al.* Revised Oxygen Evolution Reaction Activity Trends for First-Row Transition-Metal (Oxy)hydroxides in Alkaline Media. *J Phys Chem Lett* **6**, 3737-3742 (2015).
36 Batchellor, A. S., Kwon, G., Laskowski, F. A. L., Tiede, D. M. & Boettcher, S. W. Domain Structures of Ni and NiFe (Oxy)Hydroxide Oxygen-Evolution Catalysts from X-ray Pair Distribution Function Analysis. *J Phys Chem C* **121**, 25421-25429 (2017).
37 Li, N. *et al.* Influence of iron doping on tetravalent nickel content in catalytic oxygen evolving films. *P Natl Acad Sci USA* **114**, 1486-1491 (2017).
38 Louie, M. W. & Bell, A. T. An Investigation of Thin-Film Ni-Fe Oxide Catalysts for the Electrochemical Evolution of Oxygen. *J Am Chem Soc* **135**, 12329-12337 (2013).
39 Mansour, A. N., Melendres, C. A., Pankuch, M. & Brizzolara, R. A. X-Ray-Absorption Fine-Structure Spectra and the Oxidation-State of Nickel in Some of Its Oxycompounds. *J Electrochem Soc* **141**, L69-L71 (1994).
40 Görlin, M. *et al.* Tracking Catalyst Redox States and Reaction Dynamics in Ni–Fe Oxyhydroxide Oxygen Evolution Reaction Electrocatalysts: The Role of Catalyst Support and Electrolyte pH. *J. Am. Chem. Soc.* **139**, 2070-2082 (2017).
41 Seo, B. *et al.* Size-Dependent Activity Trends Combined with in Situ X-ray Absorption Spectroscopy Reveal Insights into Cobalt Oxide/Carbon Nanotube-Catalyzed Bifunctional Oxygen Electrocatalysis. *Acs Catal* **6**, 4347-4355 (2016).
42 Haas, O. *et al.* The Fe K-edge X-ray absorption characteristics of La1-xSrxFeO3-delta prepared by solid state reaction. *Mater Res Bull* **44**, 1397-1404 (2009).
43 Bergmann, A. *et al.* Reversible amorphization and the catalytically active state of crystalline Co3O4 during oxygen evolution. *Nat Commun* **6** (2015).

44 Tung, C. W. *et al.* Reversible adapting layer produces robust single-crystal electrocatalyst for oxygen evolution. *Nat Commun* **6** (2015).
45 Calvillo, L. *et al.* Insights into the durability of Co-Fe spinel oxygen evolution electrocatalysts via operando studies of the catalyst structure. *Journal of Materials Chemistry A* **6**, 7034-7041 (2018).
46 Doyle, R. L., Godwin, I. J., Brandon, M. P. & Lyons, M. E. G. Redox and electrochemical water splitting catalytic properties of hydrated metal oxide modified electrodes. *Phys Chem Chem Phys* **15**, 13737-13783 (2013).
47 Bajdich, M., Garcia-Mota, M., Vojvodic, A., Norskov, J. K. & Bell, A. T. Theoretical Investigation of the Activity of Cobalt Oxides for the Electrochemical Oxidation of Water. *J Am Chem Soc* **135**, 13521-13530 (2013).
48 Li, Y. F. & Selloni, A. Mechanism and Activity of Water Oxidation on Selected Surfaces of Pure and Fe-Doped NiOx. *Acs Catal* **4**, 1148-1153 (2014).
49 Wang, D. N. *et al.* In Situ X-ray Absorption Near-Edge Structure Study of Advanced NiFe(OH)(x) Electrocatalyst on Carbon Paper for Water Oxidation. *J Phys Chem C* **119**, 19573-19583 (2015).
50 Demourguesguerlou, L., Fournes, L. & Delmas, C. On the Iron Oxidation-State in the Iron-Substituted Gamma-Nickel Oxyhydroxides. *J Solid State Chem* **114**, 6-14 (1995).
51 Corrigan, D. A., Conell, R. S., Fierro, C. A. & Scherson, D. A. Insitu Mossbauer Study of Redox Processes in a Composite Hydroxide of Iron and Nickel. *J Phys Chem-Us* **91**, 5009-5011 (1987).
52 Chen, J. Y. C. *et al.* Operando Analysis of NiFe and Fe Oxyhydroxide Electrocatalysts for Water Oxidation: Detection of Fe4+ by Mossbauer Spectroscopy. *J Am Chem Soc* **137**, 15090-15093 (2015).
53 Axmann, P. & Glemser, O. Nickel hydroxide as a matrix for unusual valencies: The electrochemical behaviour of metal(III)-ion-substituted nickel hydroxides of the pyroaurite type. *J Alloy Compd* **246**, 232-241 (1997).
54 Degroot, F. M. F. *et al.* Oxygen 1s X-Ray-Absorption Edges of Transition-Metal Oxides. *Phys Rev B* **40**, 5715-5723 (1989).
55 Chen, S. Y. *et al.* Electron energy loss spectroscopy and ab initio investigation of iron oxide nanomaterials grown by a hydrothermal process. *Phys Rev B* **79** (2009).
56 McCrory, C. C. L., Jung, S. H., Peters, J. C. & Jaramillo, T. F. Benchmarking Heterogeneous Electrocatalysts for the Oxygen Evolution Reaction. *J Am Chem Soc* **135**, 16977-16987 (2013).
57 Batchellor, A. S. & Boettcher, S. W. Pulse-Electrodeposited Ni-Fe (Oxy)hydroxide Oxygen Evolution Electrocatalysts with High Geometric and Intrinsic Activities at Large Mass Loadings. *Acs Catal* **5**, 6680-6689 (2015).
58 Watzele, S. & Bandarenka, A. S. Quick Determination of Electroactive Surface Area of Some Oxide Electrode Materials. *Electroanal* **28**, 2394-2399 (2016).
59 Lyons, M. E. G. & Brandon, M. P. The significance of electrochemical impedance spectra recorded during active oxygen evolution for oxide covered Ni, Co and Fe electrodes in alkaline solution. *J Electroanal Chem* **631**, 62-70 (2009).
